# Supplementary material for: Essential childhood immunization in 43 low- and middle-income countries: Analysis of spatial trends and socioeconomic inequalities in vaccine coverage
Source: PLoS Med. 2023 Jan 17;20(1):e1004166. doi: 10.1371/journal.pmed.1004166 (PMC9888726; doi:10.1371/journal.pmed.1004166)
Supplement: S1 Table — (PDF) [file pmed.1004166.s001.pdf]

Table S1. STROBE Statement—Checklist of items that should be included in reports of cross-sectional studies

	Item No	Recommendation	Location in manuscript where items are reported
**Title and abstract**	1	(*a*) Indicate the study's design with a commonly used term in the title or the abstract	Abstract: Methods and Findings
		(*b*) Provide in the abstract an informative and balanced summary of what was done and what was found	Abstract: Methods and Findings
**Introduction**			
Background/rationale	2	Explain the scientific background and rationale for the investigation being reported	Introduction: P1 & P2
Objectives	3	State specific objectives, including any prespecified hypotheses	Introduction: P3
**Methods**			
Study design	4	Present key elements of study design early in the paper	Data and methods: P1-P3
Setting	5	Describe the setting, locations, and relevant dates, including periods of recruitment, exposure, follow-up, and data collection	Data and methods: P1 & P3 & S2 Table & S3 Table
Participants	6	(*a*) Give the eligibility criteria, and the sources and methods of selection of participants	Data and methods: P1 & P3
Variables	7	Clearly define all outcomes, exposures, predictors, potential confounders, and effect modifiers. Give diagnostic criteria, if applicable	Data and methods: P4 & P5
Data sources/ measurement	8	For each variable of interest, give sources of data and details of methods of assessment (measurement). Describe comparability of assessment methods if there is more than one group	Data and methods: P1 & P4-P6
Bias	9	Describe any efforts to address potential sources of bias	Data and methods: P4
Study size	10	Explain how the study size was arrived at	Data and methods: P5
Quantitative variables	11	Explain how quantitative variables were handled in the analyses. If applicable, describe which groupings were chosen and why	Data and methods: P4-P6
Statistical methods	12	(*a*) Describe all statistical methods, including those used to control for confounding	Data and methods: P7-P12
		(*b*) Describe any methods used to examine subgroups and interactions	NA
		(*c*) Explain how missing data were addressed	Data and methods: P5
		(*d*) If applicable, describe analytical methods taking account of sampling strategy	Data and methods: P1, P8 & P10
		(*e*) Describe any sensitivity analyses	Data and methods: P4
**Results**			
Participants	13*	(a) Report numbers of individuals at each stage of study—eg numbers potentially eligible, examined for eligibility, confirmed eligible, included in the study, completing follow-up, and analysed	Data and methods: P5 & S2 Table
		(b) Give reasons for non-participation at each stage	
		(c) Consider use of a flow diagram	

Descriptive data	14	(a) Give characteristics of study participants (eg demographic, clinical, social) and information on exposures and potential confounders	NA
		(b) Indicate number of participants with missing data for each variable of interest	
Outcome data	15	Report numbers of outcome events or summary measures	NA
Main results	16	(*a*) Give unadjusted estimates and, if applicable, confounder-adjusted estimates and their precision (eg, 95% confidence interval). Make clear which confounders were adjusted for and why they were included	Results: Table 1 & S3 Table
		(*b*) Report category boundaries when continuous variables were categorized	NA
		(*c*) If relevant, consider translating estimates of relative risk into absolute risk for a meaningful time period	NA
Other analyses	17	Report other analyses done—eg analyses of subgroups and interactions, and sensitivity analyses	Results: P11 and S3 Table and S4 Table
**Discussion**			
Key results	18	Summarise key results with reference to study objectives	Discussion: P2-P5
Limitations	19	Discuss limitations of the study, taking into account sources of potential bias or imprecision. Discuss both direction and magnitude of any potential bias	Discussion: P6
Interpretation	20	Give a cautious overall interpretation of results considering objectives, limitations, multiplicity of analyses, results from similar studies, and other relevant evidence	Discussion: P4, P5 & P8
Generalisability	21	Discuss the generalisability (external validity) of the study results	Discussion: P4, P5 & P8
**Other information**			
Funding	22	Give the source of funding and the role of the funders for the present study and, if applicable, for the original study on which the present article is based	Funding Statement

The STROBE checklist was retrieved from https://www.strobe-statement.org/checklists/ (accessed: Nov 9 2022). Information on the STROBE Initiative is available at www.strobe-statement.org.
