# Supplementary material for: Essential childhood immunization in 43 low- and middle-income countries: Analysis of spatial trends and socioeconomic inequalities in vaccine coverage
Source: PLoS Med. 2023 Jan 17;20(1):e1004166. doi: 10.1371/journal.pmed.1004166 (PMC9888726; doi:10.1371/journal.pmed.1004166)
Supplement: S2 Table — The subnational units are states, regions, or districts, depending on the survey. The sample includes children between 15 and 35 months of age. (PDF) [file pmed.1004166.s002.pdf]

Table S2. Overview of DHS surveys included in the analysis. The subnational units are states, regions, or districts, depending on the survey. The sample includes children between 15 and 35 months of age.

Country	Country code	Survey year	Sample size	Administrative divisions (n)
Afghanistan	AF	2015	10597	34
Albania	AL	2017-18	865	12
Angola	AO	2015-16	4797	18
Armenia	AM	2015-16	595	11
Bangladesh	BD	2017-18	2883	8
Benin	BJ	2017-18	4283	12
Burundi	BU	2016-17	4319	18
Cambodia	KH	2014	2487	19
Cameroon	CM	2018-19	3090	12
Chad	TD	2014-15	5172	21
Egypt	EG	2014	5569	25
Ethiopia	ET	2019	1801	11
Ghana	GH	2014	1987	10
Guatemala	GU	2014-15	4221	22
Guinea	GN	2018	2135	8
Haiti	HT	2016-17	2090	11
India	IA	2019-21	76079	707
Indonesia	ID	2017	6043	34
Jordan	JO	2017-18	3558	12
Kenya	KE	2014	6940	47
Lesotho	LS	2014	1029	10
Liberia	LB	2019-20	1771	15
Madagascar	MD	2021	4006	23
Malawi	MW	2015-16	5623	28
Maldives	MV	2016-17	1019	6
Mali	ML	2018	3224	9
Mauritania	MR	2019-21	3745	14
Myanmar	MM	2015-16	1556	15
Nepal	NP	2016	1710	7
Nigeria	NG	2018	10212	37
Pakistan	PK	2017-18	4075	8
Philippines	PH	2017	3523	17
Rwanda	RW	2019-20	2729	5
Senegal	SN	2019	2103	4
Sierra Leone	SL	2019	3030	16
South Africa	ZA	2016	1176	9
Tajikistan	TJ	2017	2190	5
Tanzania	TZ	2015-16	3484	30
The Gambia	GM	2019-20	2649	8
Timor-Leste	TL	2016	2416	13
Uganda	UG	2016	5127	15
Zambia	ZM	2018-19	3323	10
Zimbabwe	ZW	2015	2009	10
