# Supplementary material for: Essential childhood immunization in 43 low- and middle-income countries: Analysis of spatial trends and socioeconomic inequalities in vaccine coverage
Source: PLoS Med. 2023 Jan 17;20(1):e1004166. doi: 10.1371/journal.pmed.1004166 (PMC9888726; doi:10.1371/journal.pmed.1004166)
Supplement: S3 Table — Sampling weights were applied in all calculations. (PDF) [file pmed.1004166.s003.pdf]

Table S3. National estimates of vaccine coverage for Bacille Calmette-Guerin (BCG), diphtheria–tetanus–pertussis (DTP), oral polio vaccine (OPV), and measles-containing vaccine (MCV) among children in indicated age groups. Sampling weights were applied in all calculations.

	Age 15 to 35 months				Age 24 to 35 months			
Country	BCG	MCV	DTP	OPV	BCG	MCV	DTP	OPV
Afghanistan	0.693	0.613	0.521	0.633	0.662	0.596	0.481	0.621
Albania	0.913	0.911	0.916	0.916	0.905	0.906	0.908	0.908
Angola	0.713	0.580	0.371	0.393	0.706	0.583	0.354	0.373
Armenia	0.992	0.922	0.935	0.952	0.993	0.931	0.929	0.956
Bangladesh	0.986	0.926	0.965	0.952	0.988	0.928	0.964	0.950
Benin	0.872	0.683	0.726	0.636	0.857	0.672	0.713	0.618
Burundi	0.978	0.948	0.967	0.896	0.980	0.957	0.967	0.878
Cambodia	0.957	0.846	0.874	0.871	0.956	0.891	0.896	0.900
Cameroon	0.852	0.688	0.713	0.645	0.845	0.716	0.716	0.635
Chad	0.605	0.611	0.329	0.537	0.600	0.627	0.319	0.544
Egypt	0.991	0.451	0.971	0.976	0.993	0.489	0.971	0.976
Ethiopia	0.668	0.567	0.548	0.542	0.634	0.558	0.512	0.508
Ghana	0.968	0.896	0.874	0.824	0.966	0.900	0.858	0.798
Guatemala	0.981	0.863	0.917	0.915	0.981	0.950	0.951	0.949
Guinea	0.728	0.441	0.415	0.393	0.714	0.460	0.416	0.387
Haiti	0.820	0.659	0.561	0.540	0.814	0.689	0.559	0.537
India	0.947	0.742	0.873	0.801	0.945	0.753	0.875	0.796
Indonesia	0.911	0.829	0.781	0.742	0.910	0.848	0.793	0.755
Jordan	0.918	0.871	0.892	0.849	0.913	0.895	0.888	0.851
Kenya	0.965	0.890	0.896	0.810	0.965	0.895	0.891	0.813
Lesotho	0.973	0.939	0.862	0.744	0.966	0.957	0.875	0.743
Liberia	0.913	0.746	0.663	0.583	0.912	0.751	0.631	0.551
Madagascar	0.781	0.667	0.679	0.562	0.772	0.672	0.683	0.553
Malawi	0.971	0.926	0.919	0.771	0.969	0.933	0.914	0.750
Maldives	0.912	0.898	0.831	0.819	0.921	0.916	0.842	0.828
Mali	0.831	0.706	0.698	0.495	0.824	0.699	0.691	0.458
Mauritania	0.890	0.765	0.715	0.443	0.886	0.777	0.708	0.424
Myanmar	0.890	0.825	0.675	0.722	0.886	0.841	0.685	0.734
Nepal	0.966	0.931	0.851	0.892	0.957	0.943	0.842	0.902
Nigeria	0.662	0.570	0.494	0.454	0.644	0.576	0.476	0.439
Pakistan	0.877	0.757	0.765	0.875	0.869	0.772	0.767	0.885
Philippines	0.873	0.805	0.747	0.772	0.855	0.806	0.721	0.760
Rwanda	0.992	0.980	0.988	0.967	0.993	0.983	0.988	0.962
Senegal	0.945	0.880	0.922	0.802	0.948	0.897	0.924	0.766
Sierra Leone	0.958	0.795	0.764	0.663	0.951	0.822	0.734	0.613
South Africa	0.924	0.852	0.643	0.726	0.916	0.849	0.641	0.707
Tajikistan	0.960	0.875	0.878	0.877	0.962	0.875	0.880	0.876
Tanzania	0.958	0.884	0.883	0.799	0.955	0.894	0.880	0.780
The Gambia	0.983	0.907	0.918	0.886	0.981	0.913	0.910	0.869
Timor-Leste	0.785	0.700	0.585	0.512	0.771	0.703	0.556	0.486
Uganda	0.958	0.838	0.776	0.629	0.954	0.853	0.765	0.605
Zambia	0.975	0.920	0.915	0.789	0.974	0.935	0.912	0.777
Zimbabwe	0.881	0.822	0.818	0.814	0.866	0.821	0.808	0.805
