# Supplementary material for: Essential childhood immunization in 43 low- and middle-income countries: Analysis of spatial trends and socioeconomic inequalities in vaccine coverage
Source: PLoS Med. 2023 Jan 17;20(1):e1004166. doi: 10.1371/journal.pmed.1004166 (PMC9888726; doi:10.1371/journal.pmed.1004166)
Supplement: S4 Table — The lower and upper bounds refer to the 95% confidence intervals of W and E. Countries are ranked from the worst performing (i.e., lowest vaccination rate or highest magnitude of inequality) to the best performing. Sampling weights were applied in all calculations. (PDF) [file pmed.1004166.s004.pdf]

**Table S4.** National estimates of full immunization coverage (FIC), Wagstaff's index of inequality (W) and Erreygers' index of inequality (E) among children between 24 and 35 months of age. The lower and upper bounds refer to the 95% confidence intervals of W and E. Countries are ranked from the worst performing (i.e., lowest vaccination rate or highest magnitude of inequality) to the best performing. Sampling weights were applied in all calculations.

| Country | Sample size | FIC | Rank FIC | W | Lower bound | Upper bound | Rank W | E | Lower bound | Upper bound | Rank E |
|---|---|---|---|---|---|---|---|---|---|---|---|
| Afghanistan | 6672 | 0.384 | 7 | 0.119 | 0.091 | 0.147 | 21 | 0.112 | 0.086 | 0.139 | 16 |
| Albania | 515 | 0.900 | 40 | -0.064 | -0.231 | 0.103 | 31 | -0.023 | -0.083 | 0.037 | 38 |
| Angola | 2692 | 0.262 | 3 | 0.387 | 0.339 | 0.434 | 1 | 0.300 | 0.263 | 0.336 | 4 |
| Armenia | 332 | 0.893 | 39 | -0.173 | -0.373 | 0.028 | 15 | -0.066 | -0.143 | 0.011 | 25 |
| Bangladesh | 1659 | 0.906 | 41 | 0.143 | 0.048 | 0.238 | 17 | 0.049 | 0.016 | 0.081 | 32 |
| Benin | 2365 | 0.534 | 17 | 0.203 | 0.157 | 0.249 | 10 | 0.202 | 0.156 | 0.248 | 9 |
| Burundi | 2377 | 0.838 | 37 | -0.058 | -0.121 | 0.005 | 34 | -0.031 | -0.065 | 0.003 | 34 |
| Cambodia | 1386 | 0.850 | 38 | 0.327 | 0.243 | 0.410 | 4 | 0.166 | 0.124 | 0.209 | 11 |
| Cameroon | 1765 | 0.525 | 15 | 0.239 | 0.186 | 0.292 | 8 | 0.238 | 0.186 | 0.291 | 7 |
| Chad | 3203 | 0.254 | 2 | 0.087 | 0.041 | 0.133 | 24 | 0.066 | 0.031 | 0.101 | 26 |
| Egypt | 3088 | 0.453 | 12 | 0.077 | 0.036 | 0.118 | 27 | 0.076 | 0.036 | 0.117 | 21 |
| Ethiopia | 1110 | 0.403 | 8 | 0.315 | 0.249 | 0.382 | 5 | 0.303 | 0.239 | 0.368 | 2 |
| Ghana | 1134 | 0.729 | 29 | -0.029 | -0.105 | 0.047 | 40 | -0.023 | -0.083 | 0.037 | 39 |
| Guatemala | 2448 | 0.911 | 42 | -0.010 | -0.091 | 0.070 | 43 | -0.003 | -0.029 | 0.023 | 43 |
| Guinea | 1266 | 0.242 | 1 | 0.190 | 0.117 | 0.264 | 11 | 0.140 | 0.086 | 0.194 | 14 |
| Haiti | 1233 | 0.404 | 9 | 0.256 | 0.192 | 0.320 | 7 | 0.247 | 0.185 | 0.308 | 6 |
| India | 44238 | 0.637 | 20 | 0.075 | 0.064 | 0.086 | 28 | 0.069 | 0.059 | 0.079 | 22 |
| Indonesia | 3394 | 0.694 | 24 | 0.121 | 0.079 | 0.163 | 19 | 0.102 | 0.067 | 0.138 | 18 |
| Jordan | 2065 | 0.830 | 36 | 0.117 | 0.051 | 0.183 | 22 | 0.066 | 0.029 | 0.103 | 27 |
| Kenya | 3999 | 0.718 | 26 | 0.128 | 0.089 | 0.168 | 18 | 0.104 | 0.072 | 0.136 | 17 |
| Lesotho | 573 | 0.690 | 23 | 0.061 | -0.041 | 0.163 | 32 | 0.052 | -0.035 | 0.140 | 29 |
| Liberia | 970 | 0.447 | 11 | 0.067 | -0.006 | 0.140 | 30 | 0.067 | -0.006 | 0.139 | 24 |
| Madagascar | 2313 | 0.479 | 13 | 0.220 | 0.174 | 0.266 | 9 | 0.219 | 0.173 | 0.266 | 8 |
| Malawi | 3278 | 0.709 | 25 | 0.078 | 0.035 | 0.122 | 26 | 0.065 | 0.029 | 0.101 | 28 |
| Maldives | 591 | 0.787 | 32 | 0.074 | -0.040 | 0.187 | 29 | 0.049 | -0.027 | 0.126 | 31 |
| Mali | 1751 | 0.358 | 6 | 0.032 | -0.024 | 0.088 | 39 | 0.029 | -0.022 | 0.081 | 35 |
| Mauritania | 2216 | 0.353 | 5 | -0.106 | -0.156 | -0.056 | 23 | -0.097 | -0.142 | -0.051 | 19 |
| Myanmar | 896 | 0.637 | 19 | 0.279 | 0.202 | 0.356 | 6 | 0.258 | 0.187 | 0.329 | 5 |
| Nepal | 948 | 0.798 | 33 | -0.019 | -0.110 | 0.073 | 42 | -0.012 | -0.071 | 0.047 | 42 |
| Nigeria | 5869 | 0.277 | 4 | 0.378 | 0.347 | 0.410 | 2 | 0.303 | 0.278 | 0.328 | 3 |
| Pakistan | 2383 | 0.684 | 22 | 0.356 | 0.308 | 0.403 | 3 | 0.308 | 0.266 | 0.349 | 1 |
| Philippines | 2015 | 0.663 | 21 | 0.184 | 0.132 | 0.237 | 12 | 0.165 | 0.118 | 0.212 | 12 |
| Rwanda | 1597 | 0.946 | 43 | 0.059 | -0.066 | 0.185 | 33 | 0.012 | -0.014 | 0.038 | 41 |
| Senegal | 1219 | 0.722 | 27 | 0.163 | 0.091 | 0.235 | 16 | 0.131 | 0.073 | 0.189 | 15 |
| Sierra Leone | 1742 | 0.505 | 14 | -0.051 | -0.105 | 0.003 | 35 | -0.051 | -0.105 | 0.003 | 30 |
| South Africa | 678 | 0.584 | 18 | -0.023 | -0.111 | 0.065 | 41 | -0.022 | -0.108 | 0.063 | 40 |
| Tajikistan | 1253 | 0.826 | 35 | -0.046 | -0.129 | 0.036 | 36 | -0.027 | -0.074 | 0.021 | 37 |
| Tanzania | 1880 | 0.729 | 28 | 0.181 | 0.123 | 0.239 | 13 | 0.143 | 0.097 | 0.189 | 13 |
| The Gambia | 1500 | 0.818 | 34 | -0.046 | -0.121 | 0.030 | 37 | -0.027 | -0.072 | 0.018 | 36 |
| Timor-Leste | 1333 | 0.426 | 10 | 0.178 | 0.116 | 0.240 | 14 | 0.174 | 0.114 | 0.235 | 10 |
| Uganda | 2938 | 0.527 | 16 | -0.032 | -0.074 | 0.010 | 38 | -0.032 | -0.074 | 0.010 | 33 |
| Zambia | 1908 | 0.731 | 30 | 0.087 | 0.029 | 0.145 | 25 | 0.068 | 0.023 | 0.114 | 23 |
| Zimbabwe | 1173 | 0.757 | 31 | 0.119 | 0.042 | 0.196 | 20 | 0.088 | 0.031 | 0.144 | 20 |
