# Supplementary material for: Essential childhood immunization in 43 low- and middle-income countries: Analysis of spatial trends and socioeconomic inequalities in vaccine coverage
Source: PLoS Med. 2023 Jan 17;20(1):e1004166. doi: 10.1371/journal.pmed.1004166 (PMC9888726; doi:10.1371/journal.pmed.1004166)
Supplement: S5 Table — The lower and upper bounds refer to the 95% confidence intervals of W and E. Sampling weights were applied in all calculations. (PDF) [file pmed.1004166.s005.pdf]

Table S5. Subnational estimates of full immunization coverage (FIC), Wagstaff's (W) and Erreygers' (E) indices of inequality. The lower and upper bounds refer to the 95% confidence intervals of W and E. Sampling weights were applied in all calculations.

Country	Region	Sample size	FIC	W	Lower bound	Upper bound	E	Lower bound	Upper bound
Afghanistan	Badakhshan	279	0.654	-0.024	-0.168	0.119	-0.022	-0.152	0.108
Afghanistan	Badghis	372	0.571	0.086	-0.033	0.204	0.084	-0.032	0.200
Afghanistan	Baghlan	253	0.370	0.171	0.024	0.318	0.159	0.023	0.296
Afghanistan	Balkh	348	0.354	0.375	0.254	0.496	0.343	0.233	0.454
Afghanistan	Bamyan	240	0.641	0.108	-0.045	0.260	0.099	-0.041	0.240
Afghanistan	Daykundi	206	0.320	0.015	-0.155	0.185	0.013	-0.135	0.161
Afghanistan	Farah	428	0.253	0.510	0.393	0.626	0.385	0.297	0.473
Afghanistan	Faryab	280	0.602	0.213	0.077	0.350	0.204	0.074	0.335
Afghanistan	Ghazni	234	0.351	-0.211	-0.364	-0.057	-0.192	-0.332	-0.052
Afghanistan	Ghor	360	0.277	0.186	0.054	0.318	0.149	0.043	0.255
Afghanistan	Helmand	300	0.176	-0.021	-0.194	0.151	-0.012	-0.112	0.088
Afghanistan	Herat	300	0.521	0.270	0.142	0.398	0.270	0.142	0.398
Afghanistan	Jawzjan	313	0.305	0.142	0.004	0.281	0.121	0.003	0.238
Afghanistan	Kabul	230	0.502	0.062	-0.088	0.213	0.062	-0.088	0.213
Afghanistan	Kandahar	324	0.155	0.483	0.316	0.650	0.254	0.166	0.341
Afghanistan	Kapisa	297	0.606	0.161	0.027	0.295	0.154	0.026	0.282
Afghanistan	Khost	613	0.297	0.052	-0.049	0.152	0.043	-0.041	0.127
Afghanistan	Kunarha	308	0.340	0.194	0.058	0.330	0.174	0.052	0.296
Afghanistan	Kunduz	285	0.365	0.134	-0.005	0.273	0.124	-0.004	0.253
Afghanistan	Laghman	345	0.627	0.099	-0.027	0.225	0.092	-0.026	0.210
Afghanistan	Logar	243	0.401	0.127	-0.021	0.276	0.122	-0.020	0.265
Afghanistan	Nangarhar	408	0.641	0.237	0.122	0.353	0.219	0.113	0.325
Afghanistan	Nimroz	242	0.553	0.268	0.125	0.411	0.265	0.123	0.407
Afghanistan	Nooristan	646	0.002	-0.150	-1.085	0.784	-0.001	-0.010	0.007
Afghanistan	Paktika	325	0.592	0.050	-0.078	0.178	0.048	-0.075	0.172
Afghanistan	Paktya	456	0.236	0.117	-0.008	0.242	0.085	-0.005	0.175
Afghanistan	Panjsher	194	0.491	0.146	-0.016	0.308	0.146	-0.016	0.308
Afghanistan	Parwan	289	0.626	0.101	-0.038	0.239	0.094	-0.035	0.224
Afghanistan	Samangan	246	0.201	0.328	0.151	0.505	0.211	0.097	0.324
Afghanistan	Sar-E-Pul	265	0.358	0.275	0.133	0.416	0.252	0.122	0.383
Afghanistan	Takhar	311	0.518	-0.190	-0.317	-0.062	-0.189	-0.317	-0.062
Afghanistan	Urozgan	432	0.022	0.233	-0.143	0.609	0.020	-0.012	0.051
Afghanistan	Wardak	215	0.637	0.357	0.202	0.511	0.330	0.187	0.473
Afghanistan	Zabul	64	0.188	0.179	-0.189	0.548	0.109	-0.115	0.334
Albania	Berat	78	0.947	0.210	-0.383	0.803	0.042	-0.077	0.161
Albania	Dibër	118	0.854	-0.124	-0.426	0.178	-0.062	-0.212	0.089
Albania	Durrës	80	0.923	0.285	-0.225	0.796	0.081	-0.064	0.226
Albania	Elbasan	80	0.853	-0.073	-0.463	0.317	-0.036	-0.232	0.159
Albania	Fier	76	0.929	0.418	-0.121	0.957	0.110	-0.032	0.251
Albania	Gjirokastër	67	0.899	0.486	-0.010	0.981	0.176	-0.003	0.355
Albania	Korçë	85	1.000	0.000	0.000	0.000	0.000	0.000	0.000
Albania	Kukes	104	0.907	-0.080	-0.483	0.324	-0.027	-0.163	0.109
Albania	Lezhë	80	0.847	-0.341	-0.702	0.021	-0.177	-0.365	0.011
Albania	Shkodër	65	0.978	-0.641	-1.698	0.417	-0.056	-0.148	0.036
Albania	Tirana	79	0.878	-0.514	-0.930	-0.098	-0.221	-0.400	-0.042
Albania	Vlorë	57	0.911	-0.553	-1.099	-0.006	-0.179	-0.355	-0.002
Angola	Bengo	196	0.186	0.392	0.191	0.594	0.238	0.116	0.360
Angola	Benguela	286	0.277	0.450	0.309	0.591	0.360	0.248	0.473
Angola	Bié	284	0.114	0.585	0.384	0.786	0.236	0.155	0.317
Angola	Cabinda	202	0.284	0.354	0.183	0.524	0.288	0.149	0.426
Angola	Cuando Cubango	222	0.036	0.492	0.085	0.898	0.068	0.012	0.123
Angola	Cuanza Norte	219	0.234	0.134	-0.047	0.314	0.096	-0.034	0.225
Angola	Cuanza Sul	256	0.184	0.297	0.118	0.477	0.178	0.071	0.286
Angola	Cunene	275	0.397	-0.042	-0.182	0.098	-0.041	-0.174	0.093
Angola	Huambo	308	0.245	0.253	0.105	0.400	0.187	0.078	0.296
Angola	Huíla	285	0.220	0.515	0.364	0.666	0.353	0.249	0.457

Angola	Luanda	409	0.432	0.354	0.246	0.462	0.348	0.242	0.453
Angola	Lunda Norte	283	0.188	0.377	0.209	0.544	0.230	0.128	0.332
Angola	Lunda Sul	291	0.214	0.289	0.130	0.447	0.194	0.087	0.301
Angola	Malanje	275	0.333	0.272	0.131	0.414	0.242	0.116	0.368
Angola	Moxico	208	0.090	0.407	0.137	0.676	0.133	0.045	0.222
Angola	Namibe	266	0.271	0.331	0.180	0.483	0.262	0.142	0.381
Angola	Uíge	274	0.145	0.399	0.210	0.589	0.198	0.104	0.291
Angola	Zaire	258	0.382	0.060	-0.085	0.205	0.057	-0.081	0.194
Armenia	Aragatsotn	23	0.804	-0.683	-1.230	-0.136	-0.431	-0.776	-0.086
Armenia	Ararat	79	0.896	-0.462	-0.872	-0.051	-0.171	-0.324	-0.019
Armenia	Armavir	71	0.934	0.282	-0.264	0.827	0.069	-0.065	0.203
Armenia	Gegharkunik	22	0.788	-0.279	-0.900	0.342	-0.187	-0.603	0.229
Armenia	Kotayk	77	0.962	0.560	-0.114	1.234	0.081	-0.017	0.179
Armenia	Lori	27	0.778	0.453	-0.061	0.967	0.313	-0.042	0.668
Armenia	Shirak	59	0.930	-0.096	-0.683	0.490	-0.025	-0.178	0.128
Armenia	Syunik	32	0.795	-0.366	-0.861	0.129	-0.238	-0.560	0.084
Armenia	Tavush	60	0.893	-0.063	-0.543	0.416	-0.024	-0.208	0.160
Armenia	Vayots Dzor	47	0.963	-0.655	-1.531	0.221	-0.093	-0.217	0.031
Armenia	Yerevan	99	0.863	-0.341	-0.669	-0.014	-0.161	-0.316	-0.007
Bangladesh	Barisal	272	0.877	0.199	-0.010	0.407	0.085	-0.004	0.175
Bangladesh	Chittagong	506	0.903	0.193	0.023	0.363	0.067	0.008	0.127
Bangladesh	Dhaka	439	0.908	0.147	-0.040	0.334	0.049	-0.013	0.111
Bangladesh	Khulna	287	0.925	0.194	-0.059	0.448	0.054	-0.016	0.124
Bangladesh	Mymensingh	345	0.892	-0.087	-0.284	0.110	-0.034	-0.109	0.042
Bangladesh	Rajshahi	307	0.922	0.245	0.005	0.485	0.070	0.001	0.139
Bangladesh	Rangpur	314	0.943	0.276	0.002	0.550	0.060	0.000	0.119
Bangladesh	Sylhet	415	0.861	0.099	-0.062	0.260	0.047	-0.030	0.124
Benin	Alibori	478	0.441	0.260	0.158	0.362	0.256	0.156	0.357
Benin	Atacora	415	0.670	0.133	0.015	0.250	0.117	0.013	0.222
Benin	Atlantic	416	0.586	0.051	-0.062	0.163	0.049	-0.060	0.159
Benin	Borgou	562	0.398	0.372	0.280	0.465	0.357	0.268	0.446
Benin	Collines	326	0.662	0.172	0.041	0.304	0.154	0.036	0.272
Benin	Couffo	277	0.356	0.163	0.021	0.304	0.149	0.020	0.278
Benin	Donga	319	0.582	0.277	0.151	0.402	0.269	0.147	0.391
Benin	Littoral	303	0.651	0.136	0.000	0.272	0.123	0.000	0.247
Benin	Mono	214	0.712	0.009	-0.163	0.180	0.007	-0.133	0.148
Benin	Oueme	317	0.684	0.028	-0.109	0.166	0.025	-0.094	0.143
Benin	Plateau	256	0.369	-0.065	-0.212	0.082	-0.061	-0.198	0.076
Benin	Zou	400	0.671	0.007	-0.114	0.127	0.006	-0.101	0.112
Burundi	Bubanza	267	0.783	-0.266	-0.431	-0.100	-0.181	-0.293	-0.068
Burundi	Bujumbura Mairie	185	0.721	0.189	0.004	0.373	0.152	0.003	0.300
Burundi	Bujumbura Rural	238	0.836	0.254	0.057	0.450	0.139	0.031	0.247
Burundi	Bururi	181	0.862	0.103	-0.142	0.347	0.049	-0.068	0.166
Burundi	Cankuzo	230	0.880	0.260	0.032	0.488	0.110	0.014	0.207
Burundi	Cibitoke	261	0.629	0.034	-0.111	0.180	0.032	-0.104	0.168
Burundi	Gitega	252	0.898	0.216	-0.019	0.451	0.079	-0.007	0.165
Burundi	Karusi	251	0.891	0.117	-0.113	0.347	0.045	-0.044	0.134
Burundi	Kayanza	237	0.880	-0.306	-0.530	-0.082	-0.129	-0.223	-0.035
Burundi	Kirundo	284	0.858	0.309	0.119	0.499	0.150	0.058	0.243
Burundi	Makamba	247	0.926	0.238	-0.037	0.514	0.065	-0.010	0.140
Burundi	Muramvya	211	0.893	0.063	-0.189	0.316	0.024	-0.073	0.121
Burundi	Muyinga	290	0.910	-0.007	-0.240	0.226	-0.002	-0.079	0.074
Burundi	Mwaro	190	0.841	-0.235	-0.458	-0.012	-0.126	-0.245	-0.006
Burundi	Ngozi	248	0.797	0.002	-0.177	0.181	0.001	-0.115	0.117
Burundi	Rumonge	255	0.814	0.089	-0.093	0.272	0.054	-0.057	0.165
Burundi	Rutana	225	0.902	-0.024	-0.279	0.231	-0.008	-0.099	0.082
Burundi	Ruyigi	267	0.825	0.088	-0.094	0.271	0.051	-0.054	0.157
Cambodia	Banteay Meanchey	115	0.927	0.195	-0.214	0.603	0.052	-0.058	0.163
Cambodia	Battambang/Pailin	106	0.906	0.431	0.061	0.802	0.147	0.021	0.274
Cambodia	Kampong Cham	130	0.694	0.244	0.031	0.457	0.207	0.026	0.388
Cambodia	Kampong Chhnang	99	0.879	0.138	-0.213	0.489	0.059	-0.091	0.208
Cambodia	Kampong Speu	143	0.779	0.320	0.097	0.544	0.221	0.067	0.374
Cambodia	Kampong Thom	132	0.874	0.115	-0.184	0.414	0.050	-0.081	0.182

Cambodia	Kampot/Kep	102	0.712	0.445	0.211	0.680	0.365	0.173	0.557
Cambodia	Kandal	108	0.779	0.550	0.307	0.794	0.379	0.211	0.546
Cambodia	Kratie	159	0.734	0.364	0.168	0.561	0.284	0.131	0.438
Cambodia	Mondul Kiri/Ratanak Kiri	171	0.560	0.492	0.333	0.651	0.485	0.329	0.642
Cambodia	Otdar Meanchey	137	0.796	0.072	-0.170	0.313	0.047	-0.110	0.203
Cambodia	Phnom Penh	170	0.928	0.383	0.050	0.717	0.102	0.013	0.191
Cambodia	Preah Sihanouk/Koh Kong	136	0.874	0.032	-0.262	0.327	0.014	-0.115	0.144
Cambodia	Preah Vihear/Stung Treng	186	0.700	0.263	0.085	0.441	0.221	0.071	0.370
Cambodia	Prey Veng	106	0.713	0.307	0.069	0.545	0.251	0.056	0.446
Cambodia	Pursat	143	0.856	0.500	0.242	0.759	0.247	0.119	0.374
Cambodia	Siem Reap	133	0.798	-0.067	-0.313	0.180	-0.043	-0.201	0.116
Cambodia	Svay Rieng	108	0.824	0.084	-0.204	0.372	0.049	-0.119	0.216
Cambodia	Takeo	103	0.865	0.058	-0.272	0.388	0.027	-0.126	0.181
Cameroon	Adamawa	213	0.327	0.116	-0.049	0.281	0.102	-0.044	0.248
Cameroon	Centre	361	0.630	0.060	-0.063	0.184	0.056	-0.059	0.171
Cameroon	Douala	185	0.645	0.151	-0.022	0.324	0.138	-0.021	0.297
Cameroon	East	311	0.581	0.310	0.184	0.435	0.301	0.179	0.424
Cameroon	Far North	399	0.437	0.239	0.127	0.351	0.235	0.125	0.345
Cameroon	Littoral	169	0.547	0.114	-0.061	0.289	0.113	-0.061	0.286
Cameroon	North	385	0.323	0.118	-0.005	0.241	0.103	-0.005	0.211
Cameroon	Northwest	162	0.617	0.237	0.056	0.417	0.224	0.053	0.394
Cameroon	South	296	0.633	0.215	0.080	0.349	0.200	0.074	0.325
Cameroon	Southwest	46	0.656	0.004	-0.355	0.363	0.004	-0.320	0.328
Cameroon	West	313	0.602	0.249	0.121	0.378	0.239	0.116	0.362
Cameroon	Yaounde	250	0.567	0.195	0.052	0.338	0.192	0.051	0.332
Chad	Barh El Gazal	232	0.125	0.353	0.132	0.574	0.155	0.058	0.252
Chad	Batha	203	0.037	0.607	0.188	1.025	0.086	0.027	0.145
Chad	Borkou/Tibesti	156	0.121	0.014	-0.269	0.296	0.006	-0.114	0.126
Chad	Chari Baguirmi	257	0.043	0.108	-0.249	0.466	0.018	-0.041	0.077
Chad	Ennedi Est/Ennedi Ouest	195	0.111	-0.238	-0.499	0.023	-0.094	-0.197	0.009
Chad	Guéra	284	0.176	0.449	0.277	0.620	0.260	0.161	0.360
Chad	Hadjer-Lamis	256	0.240	0.182	0.016	0.347	0.132	0.012	0.253
Chad	Kanem	270	0.111	-0.019	-0.241	0.202	-0.008	-0.095	0.080
Chad	Lac	218	0.145	0.157	-0.062	0.377	0.078	-0.031	0.187
Chad	Logone Occidental	235	0.308	0.146	-0.017	0.308	0.124	-0.014	0.263
Chad	Logone Oriental	266	0.302	0.120	-0.037	0.278	0.101	-0.032	0.234
Chad	Mandoul	286	0.544	0.036	-0.102	0.173	0.035	-0.101	0.172
Chad	Mayo Kebbi Est	317	0.321	0.201	0.065	0.337	0.175	0.056	0.294
Chad	Mayo Kebbi Ouest	352	0.566	-0.024	-0.147	0.099	-0.024	-0.144	0.097
Chad	Moyen Chari	227	0.319	0.112	-0.054	0.278	0.097	-0.047	0.242
Chad	N'djaména	357	0.287	0.103	-0.035	0.241	0.084	-0.028	0.197
Chad	Ouaddaï	222	0.069	0.211	-0.093	0.514	0.054	-0.024	0.131
Chad	Salamat	308	0.119	0.146	-0.054	0.345	0.061	-0.023	0.145
Chad	Sila	215	0.110	0.292	0.043	0.541	0.115	0.017	0.213
Chad	Tandjilé	248	0.329	-0.120	-0.277	0.037	-0.106	-0.245	0.032
Chad	Wadi Fira	233	0.061	0.139	-0.173	0.451	0.032	-0.040	0.104
Egypt	Alexandria	155	0.401	-0.081	-0.267	0.105	-0.078	-0.257	0.101
Egypt	Assuit	296	0.351	-0.019	-0.157	0.119	-0.017	-0.143	0.109
Egypt	Aswan	227	0.655	0.105	-0.053	0.263	0.095	-0.048	0.238
Egypt	Behera	283	0.455	0.108	-0.027	0.243	0.107	-0.027	0.241
Egypt	Beni Suef	244	0.371	0.086	-0.066	0.237	0.080	-0.062	0.222
Egypt	Cairo	246	0.504	-0.023	-0.169	0.122	-0.023	-0.169	0.122
Egypt	Dakahlia	205	0.379	0.170	0.007	0.333	0.160	0.007	0.314
Egypt	Damietta	215	0.471	0.028	-0.132	0.188	0.028	-0.132	0.187
Egypt	Fayoum	269	0.266	0.113	-0.043	0.270	0.088	-0.034	0.211
Egypt	Gharbia	209	0.483	0.005	-0.152	0.163	0.005	-0.152	0.162
Egypt	Giza	245	0.556	0.011	-0.138	0.159	0.010	-0.137	0.157
Egypt	Ismailia	233	0.414	0.280	0.133	0.427	0.272	0.129	0.414
Egypt	Kafr El-Sheikh	212	0.506	0.017	-0.139	0.173	0.017	-0.139	0.173
Egypt	Kalyubia	207	0.385	-0.210	-0.372	-0.049	-0.199	-0.352	-0.046
Egypt	Luxor	224	0.589	-0.195	-0.348	-0.043	-0.189	-0.337	-0.041
Egypt	Matroh	172	0.607	0.111	-0.066	0.288	0.106	-0.063	0.275
Egypt	Menoufia	213	0.376	0.143	-0.017	0.302	0.134	-0.016	0.284

Egypt	Menya	235	0.388	-0.063	-0.218	0.091	-0.060	-0.207	0.086
Egypt	New Valley	120	0.518	-0.091	-0.299	0.117	-0.091	-0.298	0.117
Egypt	Port Said	175	0.420	-0.114	-0.288	0.060	-0.111	-0.281	0.058
Egypt	Qena	322	0.318	0.064	-0.072	0.201	0.056	-0.062	0.174
Egypt	Red Sea	111	0.378	0.145	-0.082	0.372	0.136	-0.077	0.350
Egypt	Sharkia	263	0.280	0.080	-0.076	0.236	0.065	-0.061	0.190
Egypt	Souhag	300	0.400	0.184	0.050	0.318	0.177	0.048	0.305
Egypt	Suez	256	0.468	-0.028	-0.171	0.115	-0.028	-0.171	0.115
Ethiopia	Addis Ababa	109	0.834	0.017	-0.277	0.311	0.009	-0.154	0.172
Ethiopia	Afar	214	0.125	0.245	0.012	0.477	0.107	0.005	0.209
Ethiopia	Amhara	170	0.570	0.388	0.221	0.554	0.380	0.217	0.543
Ethiopia	Benishangul-Gumuz	160	0.600	0.171	-0.011	0.353	0.164	-0.010	0.339
Ethiopia	Dire Dawa	125	0.589	0.019	-0.188	0.227	0.019	-0.182	0.219
Ethiopia	Gambela	139	0.474	0.568	0.399	0.737	0.566	0.398	0.735
Ethiopia	Harari	150	0.361	0.540	0.367	0.713	0.498	0.339	0.658
Ethiopia	Oromia	233	0.314	0.153	-0.006	0.312	0.132	-0.005	0.269
Ethiopia	Snnpr	196	0.305	0.191	0.016	0.365	0.162	0.014	0.309
Ethiopia	Somali	163	0.155	0.355	0.115	0.595	0.186	0.060	0.312
Ethiopia	Tigray	142	0.712	0.245	0.037	0.452	0.201	0.031	0.370
Ghana	Ashanti	197	0.780	-0.175	-0.369	0.020	-0.120	-0.253	0.013
Ghana	Brong Ahafo	234	0.821	-0.035	-0.228	0.159	-0.020	-0.134	0.094
Ghana	Central	211	0.650	-0.024	-0.188	0.140	-0.022	-0.171	0.127
Ghana	Eastern	189	0.751	0.165	-0.025	0.355	0.123	-0.019	0.266
Ghana	Greater Accra	160	0.795	-0.015	-0.238	0.208	-0.010	-0.155	0.135
Ghana	Northern	285	0.663	0.223	0.083	0.363	0.199	0.074	0.324
Ghana	Upper East	180	0.824	-0.235	-0.455	-0.015	-0.136	-0.264	-0.009
Ghana	Upper West	158	0.901	-0.239	-0.540	0.062	-0.085	-0.192	0.022
Ghana	Volta	181	0.800	-0.176	-0.386	0.033	-0.113	-0.247	0.021
Ghana	Western	193	0.686	-0.280	-0.452	-0.108	-0.241	-0.389	-0.093
Guatemala	Alta Verapaz	223	0.722	0.077	-0.092	0.247	0.062	-0.074	0.198
Guatemala	Baja Verapaz	181	0.891	0.229	-0.041	0.498	0.089	-0.016	0.193
Guatemala	Chimaltenango	222	0.878	-0.112	-0.345	0.121	-0.048	-0.148	0.052
Guatemala	Chiquimula	202	0.876	-0.116	-0.358	0.126	-0.050	-0.156	0.055
Guatemala	El Progreso	148	0.914	0.323	-0.008	0.654	0.101	-0.002	0.205
Guatemala	Escuintla	144	0.851	0.026	-0.241	0.292	0.013	-0.122	0.149
Guatemala	Guatemala	338	0.837	0.131	-0.035	0.298	0.072	-0.019	0.163
Guatemala	Huehuetenango	217	0.755	0.134	-0.045	0.312	0.099	-0.033	0.231
Guatemala	Izabal	158	0.714	0.169	-0.030	0.367	0.138	-0.025	0.300
Guatemala	Jalapa	221	0.871	0.164	-0.063	0.392	0.074	-0.028	0.176
Guatemala	Jutiapa	182	0.881	0.215	-0.043	0.473	0.090	-0.018	0.199
Guatemala	Petén	189	0.909	-0.030	-0.319	0.258	-0.010	-0.105	0.085
Guatemala	Quetzaltenango	166	0.793	-0.052	-0.270	0.166	-0.034	-0.177	0.109
Guatemala	Quiché	276	0.876	-0.181	-0.387	0.025	-0.079	-0.168	0.011
Guatemala	Retalhuleu	166	0.840	-0.290	-0.527	-0.053	-0.156	-0.284	-0.029
Guatemala	Sacatepéquez	141	0.908	-0.168	-0.498	0.162	-0.056	-0.167	0.054
Guatemala	San Marcos	206	0.732	0.118	-0.061	0.296	0.092	-0.048	0.232
Guatemala	Santa Rosa	150	0.859	-0.024	-0.291	0.243	-0.012	-0.141	0.118
Guatemala	Sololá	176	0.784	0.213	0.007	0.419	0.144	0.005	0.284
Guatemala	Suchitepéquez	177	0.816	-0.068	-0.288	0.153	-0.041	-0.173	0.092
Guatemala	Totonicapán	196	0.845	0.013	-0.211	0.238	0.007	-0.111	0.125
Guatemala	Zacapa	142	0.888	0.188	-0.114	0.490	0.075	-0.045	0.195
Guinea	Boké	275	0.163	0.471	0.294	0.648	0.256	0.160	0.353
Guinea	Conakry	244	0.293	0.206	0.048	0.364	0.171	0.040	0.301
Guinea	Faranah	283	0.224	0.153	-0.008	0.314	0.106	-0.006	0.218
Guinea	Kankan	331	0.421	-0.067	-0.193	0.059	-0.065	-0.188	0.058
Guinea	Kindia	281	0.146	0.390	0.204	0.577	0.195	0.102	0.288
Guinea	Labé	263	0.089	0.303	0.060	0.546	0.099	0.020	0.177
Guinea	Mamou	189	0.137	0.472	0.241	0.703	0.224	0.114	0.333
Guinea	N'zérékoré	269	0.333	0.241	0.097	0.385	0.214	0.086	0.342
Haiti	Artibonite	244	0.307	0.373	0.223	0.524	0.318	0.190	0.446
Haiti	Central	224	0.423	0.128	-0.024	0.281	0.125	-0.024	0.275
Haiti	Grande-Anse	179	0.413	0.177	0.006	0.347	0.171	0.006	0.337
Haiti	Metropolitan Area	197	0.441	0.344	0.188	0.500	0.339	0.185	0.493

Haiti	Nippes	108	0.500	0.278	0.065	0.492	0.278	0.065	0.492
Haiti	North	231	0.572	0.261	0.114	0.409	0.256	0.112	0.400
Haiti	Northeast	164	0.546	-0.123	-0.300	0.055	-0.122	-0.298	0.054
Haiti	Northwest	215	0.423	0.118	-0.038	0.274	0.115	-0.037	0.268
Haiti	South	153	0.492	0.475	0.307	0.643	0.475	0.307	0.643
Haiti	Southeast	155	0.309	0.375	0.186	0.564	0.320	0.159	0.481
Haiti	West	220	0.310	0.292	0.131	0.453	0.250	0.112	0.388
India	Andaman & Nicobar Islands-Nicobars	59	0.586	0.182	-0.119	0.482	0.176	-0.115	0.468
India	Andaman & Nicobar Islands-North & Middle Andaman	33	0.848	0.487	-0.052	1.026	0.251	-0.027	0.529
India	Andaman & Nicobar Islands-South Andaman	68	0.662	-0.049	-0.343	0.245	-0.044	-0.307	0.220
India	Andhra Pradesh-Anantapur	68	0.713	-0.038	-0.345	0.270	-0.031	-0.283	0.221
India	Andhra Pradesh-Chittoor	77	0.654	0.110	-0.164	0.383	0.099	-0.148	0.347
India	Andhra Pradesh-East Godavari	65	0.551	0.036	-0.250	0.323	0.036	-0.248	0.319
India	Andhra Pradesh-Guntur	72	0.677	-0.010	-0.299	0.280	-0.008	-0.261	0.245
India	Andhra Pradesh-Krishna	49	0.826	0.079	-0.356	0.514	0.045	-0.204	0.295
India	Andhra Pradesh-Kurnool	98	0.559	0.233	0.005	0.461	0.230	0.005	0.454
India	Andhra Pradesh-Prakasam	56	0.597	0.220	-0.088	0.528	0.212	-0.085	0.509
India	Andhra Pradesh-Sri Potti Sriramulu Nellore	81	0.606	-0.091	-0.351	0.168	-0.087	-0.335	0.161
India	Andhra Pradesh-Srikakulam	59	0.735	0.013	-0.327	0.352	0.010	-0.255	0.275
India	Andhra Pradesh-Visakhapatnam	69	0.525	-0.019	-0.296	0.258	-0.019	-0.295	0.257
India	Andhra Pradesh-Vizianagaram	64	0.543	-0.045	-0.333	0.244	-0.044	-0.330	0.242
India	Andhra Pradesh-West Godavari	61	0.671	0.095	-0.218	0.407	0.083	-0.193	0.359
India	Andhra Pradesh-Y.s.r.	97	0.652	0.146	-0.096	0.388	0.132	-0.087	0.352
India	Arunachal Pradesh-Anjaw	74	0.741	0.037	-0.267	0.341	0.028	-0.205	0.262
India	Arunachal Pradesh-Changlang	103	0.504	0.216	-0.005	0.437	0.216	-0.005	0.437
India	Arunachal Pradesh-Dibang Valley	61	0.800	0.196	-0.169	0.561	0.126	-0.108	0.359
India	Arunachal Pradesh-East Kameng	125	0.460	-0.008	-0.213	0.196	-0.008	-0.212	0.195
India	Arunachal Pradesh-East Siang	72	0.375	0.093	-0.185	0.372	0.087	-0.174	0.349
India	Arunachal Pradesh-Kra Daadi	57	0.435	0.053	-0.254	0.360	0.052	-0.250	0.354
India	Arunachal Pradesh-Kurung Kumey	78	0.651	-0.152	-0.422	0.118	-0.138	-0.383	0.108
India	Arunachal Pradesh-Lohit	109	0.479	0.274	0.061	0.487	0.273	0.061	0.486
India	Arunachal Pradesh-Longding	91	0.611	0.365	0.131	0.599	0.347	0.124	0.569
India	Arunachal Pradesh-Lower Dibang Valley	90	0.806	-0.024	-0.329	0.281	-0.015	-0.206	0.176
India	Arunachal Pradesh-Lower Subansiri	81	0.547	0.056	-0.199	0.312	0.056	-0.198	0.309
India	Arunachal Pradesh-Namsai	124	0.500	0.255	0.056	0.455	0.255	0.056	0.455
India	Arunachal Pradesh-Papum Pare	91	0.502	0.118	-0.121	0.356	0.118	-0.121	0.356
India	Arunachal Pradesh-Siang	71	0.483	0.113	-0.159	0.384	0.112	-0.159	0.383
India	Arunachal Pradesh-Tawang	97	0.463	-0.111	-0.343	0.120	-0.111	-0.341	0.120
India	Arunachal Pradesh-Tirap	99	0.559	-0.035	-0.266	0.196	-0.034	-0.262	0.194
India	Arunachal Pradesh-Upper Siang	72	0.397	0.277	0.008	0.546	0.265	0.008	0.523
India	Arunachal Pradesh-Upper Subansiri	144	0.662	0.308	0.113	0.502	0.275	0.102	0.449
India	Arunachal Pradesh-West Kameng	67	0.269	0.036	-0.281	0.352	0.028	-0.221	0.277
India	Arunachal Pradesh-West Siang	91	0.358	0.050	-0.200	0.300	0.046	-0.184	0.276
India	Assam-Baksa	120	0.483	-0.067	-0.275	0.141	-0.067	-0.275	0.141
India	Assam-Barpeta	137	0.386	0.272	0.077	0.466	0.258	0.073	0.442
India	Assam-Biswanath	95	0.568	0.148	-0.087	0.383	0.145	-0.086	0.376
India	Assam-Bongaigaon	113	0.385	-0.096	-0.316	0.124	-0.091	-0.299	0.117
India	Assam-Cachar	125	0.395	-0.161	-0.367	0.046	-0.154	-0.351	0.044
India	Assam-Charaideo	88	0.486	0.022	-0.222	0.266	0.022	-0.222	0.266
India	Assam-Chirang	117	0.428	-0.054	-0.267	0.159	-0.053	-0.261	0.156
India	Assam-Darrang	128	0.424	0.099	-0.104	0.302	0.097	-0.102	0.295
India	Assam-Dhemaji	94	0.336	0.194	-0.052	0.441	0.173	-0.047	0.393
India	Assam-Dhubri	146	0.522	0.199	0.013	0.385	0.199	0.013	0.384
India	Assam-Dibrugarh	93	0.548	-0.239	-0.472	-0.006	-0.237	-0.468	-0.006
India	Assam-Dima Hasao	86	0.381	-0.076	-0.330	0.177	-0.072	-0.311	0.167
India	Assam-Goalpara	118	0.468	0.083	-0.127	0.293	0.082	-0.127	0.292
India	Assam-Golaghat	75	0.549	0.288	0.030	0.546	0.285	0.030	0.540
India	Assam-Hailakandi	122	0.415	-0.024	-0.234	0.185	-0.024	-0.227	0.180
India	Assam-Hojai	117	0.430	0.124	-0.088	0.336	0.122	-0.086	0.330
India	Assam-Jorhat	91	0.486	-0.279	-0.512	-0.046	-0.279	-0.512	-0.046
India	Assam-Kamrup	96	0.350	0.035	-0.210	0.279	0.031	-0.191	0.254
India	Assam-Kamrup Metropolitan	69	0.312	0.112	-0.186	0.409	0.096	-0.159	0.351

India	Assam-Karbi Anglong	102	0.313	-0.008	-0.252	0.236	-0.007	-0.217	0.203
India	Assam-Karimganj	144	0.404	0.172	-0.019	0.364	0.166	-0.018	0.350
India	Assam-Kokrajhar	103	0.312	-0.176	-0.417	0.065	-0.151	-0.357	0.056
India	Assam-Lakhimpur	90	0.545	-0.065	-0.307	0.177	-0.064	-0.304	0.175
India	Assam-Majuli	93	0.356	0.214	-0.030	0.458	0.196	-0.028	0.420
India	Assam-Morigaon	128	0.523	0.118	-0.082	0.319	0.118	-0.082	0.318
India	Assam-Nagaon	143	0.417	0.050	-0.143	0.243	0.049	-0.139	0.236
India	Assam-Nalbari	77	0.430	0.094	-0.169	0.357	0.093	-0.165	0.351
India	Assam-Sivasagar	88	0.547	0.069	-0.175	0.314	0.069	-0.174	0.311
India	Assam-Sonitpur	93	0.572	0.082	-0.157	0.322	0.081	-0.153	0.315
India	Assam-South Salmara Mancachar	160	0.352	0.090	-0.098	0.278	0.082	-0.089	0.254
India	Assam-Tinsukia	90	0.454	-0.002	-0.244	0.240	-0.002	-0.242	0.238
India	Assam-Udalguri	90	0.371	0.148	-0.100	0.396	0.138	-0.093	0.370
India	Assam-West Karbi Anglong	115	0.355	-0.052	-0.275	0.170	-0.048	-0.251	0.156
India	Bihar-Araria	267	0.451	0.009	-0.130	0.149	0.009	-0.129	0.148
India	Bihar-Arwal	196	0.771	0.155	-0.037	0.347	0.109	-0.026	0.245
India	Bihar-Aurangabad	152	0.812	-0.007	-0.244	0.229	-0.004	-0.149	0.140
India	Bihar-Banka	166	0.788	0.123	-0.093	0.339	0.082	-0.062	0.226
India	Bihar-Begusarai	177	0.519	0.097	-0.073	0.268	0.097	-0.073	0.267
India	Bihar-Bhagalpur	171	0.627	-0.049	-0.229	0.131	-0.046	-0.214	0.122
India	Bihar-Bhojpur	188	0.500	0.065	-0.100	0.231	0.065	-0.100	0.231
India	Bihar-Buxer	172	0.761	0.227	0.026	0.427	0.165	0.019	0.311
India	Bihar-Darbhanga	179	0.604	0.033	-0.140	0.207	0.032	-0.134	0.198
India	Bihar-Gaya	167	0.698	-0.034	-0.225	0.158	-0.028	-0.190	0.133
India	Bihar-Gopalganj	165	0.719	0.189	-0.006	0.384	0.153	-0.005	0.311
India	Bihar-Jamui	187	0.576	0.210	0.044	0.376	0.205	0.043	0.367
India	Bihar-Jehanabad	149	0.646	0.209	0.017	0.402	0.191	0.016	0.367
India	Bihar-Kaimur (Bhabua)	169	0.895	-0.187	-0.471	0.097	-0.071	-0.178	0.036
India	Bihar-Katihar	192	0.511	-0.044	-0.208	0.120	-0.044	-0.208	0.120
India	Bihar-Khagaria	244	0.568	0.195	0.050	0.340	0.191	0.049	0.333
India	Bihar-Kishanganj	193	0.439	-0.010	-0.175	0.155	-0.010	-0.173	0.152
India	Bihar-Lakhisarai	187	0.629	0.007	-0.166	0.179	0.006	-0.155	0.167
India	Bihar-Madhepura	207	0.617	-0.004	-0.167	0.158	-0.004	-0.158	0.150
India	Bihar-Madhubani	167	0.578	-0.006	-0.184	0.173	-0.006	-0.180	0.168
India	Bihar-Munger	173	0.655	-0.073	-0.255	0.109	-0.066	-0.230	0.098
India	Bihar-Muzaffarpur	180	0.690	-0.028	-0.211	0.156	-0.024	-0.180	0.133
India	Bihar-Nalanda	160	0.607	-0.038	-0.222	0.146	-0.036	-0.212	0.139
India	Bihar-Nawada	183	0.684	0.051	-0.130	0.232	0.044	-0.112	0.200
India	Bihar-Pashchim Champaran	157	0.591	-0.141	-0.325	0.042	-0.137	-0.314	0.041
India	Bihar-Patna	138	0.326	-0.043	-0.250	0.164	-0.038	-0.220	0.144
India	Bihar-Purba Champaran	174	0.614	0.183	0.008	0.359	0.174	0.008	0.340
India	Bihar-Purnia	197	0.347	0.276	0.110	0.442	0.250	0.100	0.400
India	Bihar-Rohtas	145	0.862	0.317	0.047	0.586	0.150	0.022	0.278
India	Bihar-Saharsa	202	0.568	-0.066	-0.228	0.095	-0.065	-0.224	0.093
India	Bihar-Samastipur	168	0.568	0.046	-0.131	0.223	0.045	-0.129	0.219
India	Bihar-Saran	180	0.441	0.088	-0.082	0.259	0.087	-0.081	0.255
India	Bihar-Sheikhpura	159	0.729	0.002	-0.201	0.205	0.001	-0.159	0.162
India	Bihar-Sheohar	139	0.610	0.010	-0.188	0.208	0.010	-0.179	0.198
India	Bihar-Sitamarhi	195	0.632	0.272	0.107	0.436	0.253	0.100	0.406
India	Bihar-Siwan	140	0.735	0.006	-0.213	0.224	0.004	-0.166	0.175
India	Bihar-Supaul	219	0.654	-0.068	-0.229	0.093	-0.062	-0.207	0.084
India	Bihar-Vaishali	178	0.215	0.037	-0.170	0.245	0.025	-0.115	0.165
India	Chandigarh-Chandigarh	54	0.723	0.160	-0.188	0.507	0.128	-0.151	0.407
India	Chhattisgarh-Balod	78	0.750	-0.203	-0.499	0.093	-0.152	-0.374	0.070
India	Chhattisgarh-Baloda Bazar	107	0.516	0.161	-0.058	0.380	0.161	-0.058	0.379
India	Chhattisgarh-Balrampur	127	0.581	0.049	-0.156	0.254	0.048	-0.152	0.248
India	Chhattisgarh-Bastar	116	0.394	0.411	0.208	0.615	0.393	0.199	0.587
India	Chhattisgarh-Bemetara	116	0.684	-0.099	-0.326	0.129	-0.085	-0.282	0.111
India	Chhattisgarh-Bijapur	131	0.734	0.296	0.077	0.516	0.232	0.060	0.403
India	Chhattisgarh-Bilaspur	96	0.394	0.078	-0.160	0.316	0.074	-0.153	0.302
India	Chhattisgarh-Dantewada	103	0.608	0.269	0.045	0.494	0.257	0.043	0.471
India	Chhattisgarh-Dhamtari	83	0.798	0.067	-0.246	0.380	0.043	-0.158	0.245
India	Chhattisgarh-Durg	99	0.584	0.007	-0.226	0.240	0.007	-0.220	0.233

India	Chhattisgarh-Gariyaband	100	0.726	0.125	-0.130	0.380	0.099	-0.104	0.302
India	Chhattisgarh-Janjgir-Champa	92	0.710	-0.076	-0.339	0.186	-0.063	-0.279	0.153
India	Chhattisgarh-Jashpur	121	0.755	0.285	0.049	0.521	0.211	0.036	0.385
India	Chhattisgarh-Kabeerdham	96	0.735	0.178	-0.084	0.440	0.139	-0.065	0.343
India	Chhattisgarh-Kodagaon	100	0.506	0.157	-0.070	0.383	0.157	-0.070	0.383
India	Chhattisgarh-Korba	95	0.476	0.366	0.143	0.589	0.365	0.143	0.588
India	Chhattisgarh-Koriya	106	0.715	0.065	-0.181	0.310	0.053	-0.147	0.253
India	Chhattisgarh-Mahasamund	93	0.690	0.137	-0.118	0.392	0.117	-0.101	0.335
India	Chhattisgarh-Mungeli	96	0.669	0.043	-0.205	0.291	0.038	-0.181	0.258
India	Chhattisgarh-Narayanpur	108	0.763	0.106	-0.152	0.363	0.076	-0.110	0.263
India	Chhattisgarh-Raigarh	85	0.676	0.010	-0.256	0.275	0.009	-0.224	0.241
India	Chhattisgarh-Raipur	94	0.766	-0.013	-0.292	0.266	-0.009	-0.209	0.190
India	Chhattisgarh-Rajnandgaon	104	0.784	-0.369	-0.631	-0.106	-0.250	-0.428	-0.072
India	Chhattisgarh-Sukma	105	0.722	-0.009	-0.258	0.240	-0.007	-0.207	0.193
India	Chhattisgarh-Surajpur	126	0.708	-0.179	-0.400	0.043	-0.148	-0.331	0.035
India	Chhattisgarh-Surguja	127	0.709	0.016	-0.207	0.238	0.013	-0.171	0.197
India	Chhattisgarh-Uttar Bastar Kanker	80	0.742	-0.138	-0.430	0.153	-0.106	-0.329	0.117
India	Dadra & Nagar Haveli & Daman & Diu-Dadra & Nagar Haveli	121	0.899	-0.283	-0.624	0.057	-0.103	-0.226	0.021
India	Dadra & Nagar Haveli & Daman & Diu-Daman	68	0.618	0.265	-0.015	0.544	0.250	-0.014	0.514
India	Dadra & Nagar Haveli & Daman & Diu-Diu	85	0.825	0.294	-0.027	0.615	0.170	-0.015	0.355
India	Goa-North Goa	63	0.431	0.118	-0.173	0.409	0.116	-0.170	0.401
India	Goa-South Goa	50	0.381	-0.132	-0.466	0.202	-0.124	-0.440	0.191
India	Gujarat-Ahmadabad	79	0.601	-0.267	-0.523	-0.010	-0.256	-0.502	-0.010
India	Gujarat-Amreli	74	0.531	0.078	-0.189	0.344	0.078	-0.188	0.343
India	Gujarat-Anand	89	0.583	0.162	-0.082	0.405	0.157	-0.080	0.394
India	Gujarat-Aravali	121	0.551	0.090	-0.118	0.298	0.089	-0.116	0.295
India	Gujarat-Banas Kantha	121	0.319	0.164	-0.057	0.385	0.142	-0.049	0.334
India	Gujarat-Bharuch	98	0.477	0.132	-0.098	0.362	0.132	-0.097	0.361
India	Gujarat-Bhavnagar	94	0.354	-0.103	-0.349	0.143	-0.094	-0.319	0.131
India	Gujarat-Botad	100	0.314	0.179	-0.065	0.423	0.154	-0.056	0.364
India	Gujarat-Chhota Udaipur	98	0.769	-0.177	-0.449	0.095	-0.126	-0.319	0.067
India	Gujarat-Devbhumi Dwarka	112	0.556	-0.207	-0.421	0.006	-0.205	-0.416	0.006
India	Gujarat-Dohad	158	0.510	-0.213	-0.391	-0.035	-0.213	-0.391	-0.035
India	Gujarat-Gandhinagar	82	0.650	0.245	-0.014	0.505	0.223	-0.013	0.460
India	Gujarat-Gir Somnath	106	0.334	0.052	-0.183	0.287	0.046	-0.163	0.255
India	Gujarat-Jamnagar	76	0.777	0.222	-0.090	0.534	0.154	-0.062	0.370
India	Gujarat-Junagadh	89	0.733	-0.064	-0.338	0.210	-0.050	-0.265	0.164
India	Gujarat-Kachchh	112	0.605	0.117	-0.102	0.337	0.112	-0.098	0.322
India	Gujarat-Kheda	101	0.509	0.273	0.052	0.494	0.273	0.052	0.494
India	Gujarat-Mahesana	76	0.471	-0.085	-0.348	0.177	-0.085	-0.347	0.177
India	Gujarat-Mahisagar	107	0.743	-0.090	-0.342	0.162	-0.069	-0.262	0.123
India	Gujarat-Morbi	111	0.637	0.094	-0.130	0.319	0.087	-0.120	0.295
India	Gujarat-Narmada	107	0.746	0.306	0.060	0.553	0.232	0.045	0.419
India	Gujarat-Navsari	73	0.874	-0.128	-0.531	0.275	-0.057	-0.235	0.121
India	Gujarat-Panch Mahals	122	0.846	0.206	-0.078	0.490	0.107	-0.040	0.255
India	Gujarat-Patan	110	0.707	0.142	-0.096	0.379	0.117	-0.080	0.314
India	Gujarat-Porbandar	76	0.781	-0.239	-0.552	0.074	-0.164	-0.378	0.051
India	Gujarat-Rajkot	81	0.465	0.266	0.018	0.515	0.265	0.018	0.512
India	Gujarat-Sabar Kantha	101	0.790	-0.030	-0.309	0.249	-0.020	-0.205	0.165
India	Gujarat-Surat	93	0.827	-0.106	-0.419	0.207	-0.061	-0.240	0.118
India	Gujarat-Surendranagar	100	0.584	0.122	-0.109	0.352	0.118	-0.106	0.342
India	Gujarat-Tapi	64	0.841	0.239	-0.149	0.627	0.128	-0.080	0.336
India	Gujarat-The Dangs	106	0.866	0.252	-0.071	0.574	0.117	-0.033	0.266
India	Gujarat-Vadodara	79	0.513	0.145	-0.110	0.401	0.145	-0.110	0.401
India	Gujarat-Valsad	109	0.830	-0.154	-0.444	0.136	-0.087	-0.250	0.077
India	Haryana-Ambala	91	0.525	-0.018	-0.258	0.222	-0.018	-0.257	0.222
India	Haryana-Bhiwani	118	0.542	0.004	-0.207	0.215	0.004	-0.205	0.214
India	Haryana-Charkhi Dadri	91	0.644	0.011	-0.239	0.262	0.010	-0.219	0.240
India	Haryana-Faridabad	96	0.557	0.131	-0.102	0.365	0.130	-0.101	0.360
India	Haryana-Fatehabad	109	0.757	0.004	-0.251	0.259	0.003	-0.185	0.191
India	Haryana-Gurgaon	77	0.487	0.199	-0.058	0.457	0.199	-0.058	0.457

India	Haryana-Hisar	113	0.655	-0.069	-0.295	0.156	-0.062	-0.266	0.141
India	Haryana-Jhajjar	86	0.681	0.180	-0.082	0.442	0.156	-0.071	0.384
India	Haryana-Jind	96	0.691	0.044	-0.208	0.297	0.038	-0.178	0.254
India	Haryana-Kaithal	88	0.737	-0.030	-0.307	0.247	-0.023	-0.238	0.192
India	Haryana-Karnal	124	0.820	0.049	-0.218	0.315	0.029	-0.129	0.186
India	Haryana-Kurukshetra	121	0.661	-0.042	-0.261	0.177	-0.038	-0.234	0.159
India	Haryana-Mahendragarh	87	0.540	0.146	-0.099	0.390	0.145	-0.098	0.387
India	Haryana-Mewat	218	0.391	0.136	-0.020	0.293	0.130	-0.019	0.279
India	Haryana-Palwal	161	0.487	0.147	-0.031	0.326	0.147	-0.031	0.325
India	Haryana-Panchkula	54	0.775	-0.086	-0.461	0.289	-0.060	-0.321	0.202
India	Haryana-Panipat	108	0.621	0.152	-0.073	0.377	0.143	-0.069	0.354
India	Haryana-Rewari	87	0.618	0.058	-0.194	0.311	0.055	-0.183	0.293
India	Haryana-Rohtak	97	0.612	-0.022	-0.260	0.217	-0.021	-0.247	0.206
India	Haryana-Sirsa	40	0.740	0.086	-0.332	0.503	0.066	-0.255	0.387
India	Haryana-Sonipat	97	0.577	0.172	-0.060	0.405	0.168	-0.059	0.395
India	Haryana-Yamunanagar	130	0.585	-0.021	-0.224	0.182	-0.020	-0.218	0.177
India	Himachal Pradesh-Bilaspur	69	0.811	0.139	-0.212	0.491	0.085	-0.130	0.301
India	Himachal Pradesh-Chamba	80	0.757	0.214	-0.082	0.509	0.157	-0.060	0.374
India	Himachal Pradesh-Hamirpur	51	0.580	0.166	-0.158	0.490	0.162	-0.154	0.478
India	Himachal Pradesh-Kangra	68	0.503	-0.034	-0.313	0.244	-0.034	-0.313	0.244
India	Himachal Pradesh-Kinnaur	52	0.602	0.068	-0.258	0.395	0.066	-0.247	0.378
India	Himachal Pradesh-Kullu	64	0.559	0.124	-0.163	0.412	0.123	-0.161	0.406
India	Himachal Pradesh-Lahul & Spiti	73	0.545	0.202	-0.063	0.468	0.201	-0.063	0.464
India	Himachal Pradesh-Mandi	75	0.952	0.046	-0.576	0.668	0.008	-0.104	0.121
India	Himachal Pradesh-Shimla	65	0.734	0.019	-0.303	0.342	0.015	-0.237	0.267
India	Himachal Pradesh-Sirmaur	98	0.782	-0.048	-0.327	0.232	-0.032	-0.223	0.158
India	Himachal Pradesh-Solan	71	0.856	0.353	-0.026	0.731	0.174	-0.013	0.361
India	Himachal Pradesh-Una	84	0.566	0.273	0.028	0.518	0.269	0.028	0.509
India	Jammu & Kashmir-Anantnag	74	0.665	0.057	-0.225	0.339	0.051	-0.201	0.302
India	Jammu & Kashmir-Badgam	98	0.805	-0.193	-0.482	0.096	-0.121	-0.303	0.060
India	Jammu & Kashmir-Bandipore	88	0.699	0.122	-0.143	0.387	0.102	-0.120	0.325
India	Jammu & Kashmir-Baramula	113	0.607	-0.067	-0.286	0.153	-0.064	-0.273	0.146
India	Jammu & Kashmir-Doda	104	0.648	0.167	-0.066	0.399	0.152	-0.060	0.364
India	Jammu & Kashmir-Ganderbal	126	0.695	-0.014	-0.234	0.207	-0.012	-0.199	0.176
India	Jammu & Kashmir-Jammu	82	0.721	0.272	-0.004	0.548	0.219	-0.003	0.441
India	Jammu & Kashmir-Kathua	58	0.704	-0.296	-0.618	0.026	-0.246	-0.515	0.022
India	Jammu & Kashmir-Kishtwar	75	0.708	0.323	0.041	0.605	0.267	0.034	0.500
India	Jammu & Kashmir-Kulgam	106	0.876	0.330	-0.001	0.661	0.143	0.000	0.287
India	Jammu & Kashmir-Kupwara	102	0.872	0.160	-0.177	0.497	0.071	-0.079	0.222
India	Jammu & Kashmir-Pulwama	80	0.891	0.212	-0.197	0.622	0.082	-0.076	0.241
India	Jammu & Kashmir-Punch	112	0.789	-0.133	-0.397	0.130	-0.089	-0.264	0.087
India	Jammu & Kashmir-Rajouri	113	0.861	0.033	-0.278	0.343	0.016	-0.133	0.164
India	Jammu & Kashmir-Ramban	123	0.630	0.154	-0.057	0.365	0.144	-0.053	0.341
India	Jammu & Kashmir-Reasi	110	0.730	0.867	0.684	1.050	0.683	0.539	0.827
India	Jammu & Kashmir-Samba	100	0.805	0.091	-0.197	0.379	0.057	-0.124	0.238
India	Jammu & Kashmir-Shupiyan	90	0.905	0.315	-0.092	0.722	0.108	-0.031	0.247
India	Jammu & Kashmir-Srinagar	60	0.864	0.175	-0.256	0.606	0.082	-0.120	0.285
India	Jammu & Kashmir-Udhampur	104	0.746	-0.003	-0.261	0.254	-0.002	-0.197	0.193
India	Jharkhand-Bokaro	110	0.692	-0.019	-0.255	0.216	-0.017	-0.218	0.185
India	Jharkhand-Chatra	162	0.673	0.157	-0.032	0.346	0.138	-0.028	0.305
India	Jharkhand-Deoghar	156	0.466	-0.024	-0.207	0.159	-0.024	-0.206	0.158
India	Jharkhand-Dhanbad	116	0.691	0.084	-0.145	0.313	0.072	-0.124	0.267
India	Jharkhand-Dumka	127	0.738	0.048	-0.182	0.278	0.037	-0.141	0.215
India	Jharkhand-Garhwa	154	0.669	0.165	-0.028	0.359	0.146	-0.025	0.318
India	Jharkhand-Giridih	157	0.540	0.217	0.038	0.397	0.216	0.038	0.394
India	Jharkhand-Godda	128	0.379	0.175	-0.030	0.381	0.165	-0.028	0.359
India	Jharkhand-Gumla	134	0.729	-0.090	-0.311	0.131	-0.071	-0.246	0.103
India	Jharkhand-Hazaribagh	171	0.614	0.094	-0.085	0.272	0.089	-0.080	0.258
India	Jharkhand-Jamtara	111	0.688	-0.080	-0.313	0.154	-0.069	-0.269	0.132
India	Jharkhand-Khunti	106	0.579	0.262	0.043	0.481	0.256	0.042	0.469
India	Jharkhand-Kodarma	176	0.593	-0.023	-0.197	0.152	-0.022	-0.191	0.147
India	Jharkhand-Latehar	155	0.703	0.107	-0.093	0.307	0.089	-0.077	0.256
India	Jharkhand-Lohardaga	110	0.843	0.442	0.155	0.730	0.234	0.082	0.386

India	Jharkhand-Pakur	155	0.626	0.109	-0.079	0.297	0.102	-0.074	0.279
India	Jharkhand-Palamu	163	0.712	0.153	-0.042	0.349	0.126	-0.035	0.286
India	Jharkhand-Pashchimi Singhbhum	157	0.722	0.092	-0.111	0.294	0.074	-0.089	0.236
India	Jharkhand-Purbi Singhbhum	104	0.795	0.022	-0.255	0.300	0.015	-0.166	0.195
India	Jharkhand-Ramgarh	120	0.740	0.024	-0.213	0.261	0.019	-0.164	0.201
India	Jharkhand-Ranchi	103	0.659	-0.232	-0.465	0.002	-0.208	-0.418	0.001
India	Jharkhand-Sahibganj	186	0.608	-0.048	-0.218	0.123	-0.045	-0.208	0.117
India	Jharkhand-Saraikela-Kharsawan	101	0.789	-0.143	-0.420	0.134	-0.095	-0.280	0.090
India	Jharkhand-Simdega	123	0.655	0.244	0.032	0.456	0.221	0.029	0.412
India	Karnataka-Bagalkot	108	0.608	0.063	-0.161	0.288	0.060	-0.154	0.275
India	Karnataka-Bangalore	61	0.513	0.044	-0.251	0.338	0.044	-0.250	0.338
India	Karnataka-Bangalore Rural	91	0.841	-0.075	-0.403	0.252	-0.040	-0.215	0.135
India	Karnataka-Belgaum	118	0.636	0.048	-0.170	0.266	0.045	-0.157	0.247
India	Karnataka-Bellary	86	0.571	0.146	-0.102	0.393	0.143	-0.100	0.385
India	Karnataka-Bidar	117	0.502	-0.054	-0.265	0.157	-0.054	-0.265	0.157
India	Karnataka-Bijapur	118	0.577	0.000	-0.213	0.213	0.000	-0.208	0.208
India	Karnataka-Chamarajanagar	69	0.827	-0.044	-0.410	0.321	-0.025	-0.234	0.184
India	Karnataka-Chikkaballapura	61	0.750	0.225	-0.111	0.560	0.169	-0.083	0.420
India	Karnataka-Chikmagalur	75	0.723	-0.010	-0.306	0.286	-0.008	-0.245	0.229
India	Karnataka-Chitradurga	78	0.852	0.418	0.064	0.771	0.211	0.032	0.389
India	Karnataka-Dakshina Kannada	71	0.817	-0.186	-0.536	0.164	-0.111	-0.320	0.098
India	Karnataka-Davanagere	88	0.610	0.017	-0.233	0.267	0.017	-0.222	0.255
India	Karnataka-Dharwad	86	0.743	-0.061	-0.344	0.221	-0.047	-0.262	0.169
India	Karnataka-Gadag	107	0.669	-0.078	-0.312	0.156	-0.069	-0.277	0.138
India	Karnataka-Gulbarga	128	0.486	0.128	-0.073	0.328	0.128	-0.073	0.328
India	Karnataka-Hassan	66	0.762	-0.147	-0.477	0.184	-0.106	-0.346	0.133
India	Karnataka-Haveri	112	0.749	0.186	-0.061	0.432	0.140	-0.046	0.325
India	Karnataka-Kodagu	71	0.721	0.098	-0.204	0.401	0.079	-0.164	0.323
India	Karnataka-Kolar	89	0.835	-0.024	-0.351	0.303	-0.013	-0.193	0.167
India	Karnataka-Koppal	118	0.511	-0.050	-0.260	0.160	-0.050	-0.260	0.160
India	Karnataka-Mandya	56	0.899	0.103	-0.408	0.613	0.037	-0.148	0.223
India	Karnataka-Mysore	69	0.832	0.009	-0.362	0.379	0.005	-0.202	0.211
India	Karnataka-Raichur	138	0.641	0.099	-0.103	0.300	0.091	-0.095	0.276
India	Karnataka-Ramanagara	49	0.851	0.010	-0.453	0.474	0.005	-0.230	0.240
India	Karnataka-Shimoga	85	0.756	-0.087	-0.375	0.202	-0.064	-0.277	0.149
India	Karnataka-Tumkur	70	0.889	0.535	0.117	0.954	0.210	0.046	0.375
India	Karnataka-Udupi	88	0.815	0.104	-0.210	0.417	0.063	-0.127	0.252
India	Karnataka-Uttara Kannada	89	0.838	0.044	-0.285	0.373	0.024	-0.155	0.203
India	Karnataka-Yadgir	163	0.635	0.159	-0.025	0.343	0.147	-0.023	0.318
India	Kerala-Alappuzha	45	0.449	0.109	-0.236	0.454	0.108	-0.234	0.450
India	Kerala-Ernakulam	55	0.399	0.018	-0.299	0.336	0.018	-0.287	0.322
India	Kerala-Idukki	48	0.371	0.083	-0.261	0.428	0.078	-0.244	0.399
India	Kerala-Kannur	94	0.311	-0.221	-0.472	0.029	-0.190	-0.405	0.025
India	Kerala-Kasaragod	88	0.519	0.106	-0.137	0.349	0.106	-0.137	0.349
India	Kerala-Kollam	50	0.459	-0.013	-0.341	0.315	-0.013	-0.338	0.313
India	Kerala-Kottayam	45	0.468	-0.171	-0.513	0.171	-0.170	-0.511	0.170
India	Kerala-Kozhikode	73	0.294	0.212	-0.079	0.502	0.176	-0.065	0.417
India	Kerala-Malappuram	100	0.449	0.006	-0.223	0.236	0.006	-0.221	0.234
India	Kerala-Palakkad	75	0.398	0.041	-0.230	0.311	0.039	-0.220	0.298
India	Kerala-Pathanamthitta	33	0.408	-0.124	-0.535	0.287	-0.120	-0.517	0.277
India	Kerala-Thiruvananthapuram	60	0.389	0.006	-0.299	0.310	0.005	-0.284	0.295
India	Kerala-Thrissur	44	0.293	-0.284	-0.658	0.090	-0.235	-0.545	0.075
India	Kerala-Wayanad	72	0.581	-0.233	-0.502	0.035	-0.227	-0.489	0.034
India	Ladakh-Kargil	77	0.855	-0.155	-0.524	0.214	-0.077	-0.260	0.106
India	Ladakh-Leh	83	0.694	0.014	-0.259	0.287	0.012	-0.220	0.243
India	Lakshadweep-Lakshadweep	83	0.348	0.221	-0.038	0.481	0.201	-0.035	0.436
India	Madhya Pradesh-Agar Malwa	45	0.721	0.180	-0.201	0.561	0.145	-0.161	0.451
India	Madhya Pradesh-Alirajpur	148	0.696	0.014	-0.190	0.217	0.012	-0.161	0.184
India	Madhya Pradesh-Anuppur	100	0.756	0.089	-0.176	0.354	0.066	-0.130	0.262
India	Madhya Pradesh-Ashoknagar	98	0.664	0.074	-0.170	0.318	0.066	-0.152	0.284
India	Madhya Pradesh-Balaghat	79	0.662	0.500	0.251	0.748	0.447	0.225	0.670
India	Madhya Pradesh-Barwani	177	0.656	0.099	-0.081	0.278	0.089	-0.073	0.251
India	Madhya Pradesh-Betul	122	0.743	0.083	-0.153	0.319	0.063	-0.117	0.243

India	Madhya Pradesh-Bhind	121	0.630	-0.063	-0.277	0.152	-0.058	-0.258	0.142
India	Madhya Pradesh-Bhopal	36	0.529	-0.036	-0.424	0.352	-0.035	-0.422	0.351
India	Madhya Pradesh-Burhanpur	131	0.797	0.239	-0.005	0.484	0.155	-0.003	0.313
India	Madhya Pradesh-Chhatarpur	125	0.630	0.270	0.064	0.476	0.251	0.060	0.443
India	Madhya Pradesh-Chhindwara	86	0.656	0.414	0.169	0.658	0.374	0.153	0.594
India	Madhya Pradesh-Damoh	108	0.604	-0.097	-0.321	0.126	-0.093	-0.308	0.121
India	Madhya Pradesh-Datia	100	0.682	0.137	-0.107	0.381	0.119	-0.093	0.330
India	Madhya Pradesh-Dewas	102	0.706	0.160	-0.086	0.407	0.133	-0.071	0.338
India	Madhya Pradesh-Dhar	72	0.679	0.050	-0.240	0.339	0.043	-0.209	0.295
India	Madhya Pradesh-Dindori	115	0.654	0.225	0.005	0.445	0.203	0.004	0.403
India	Madhya Pradesh-Guna	147	0.784	0.007	-0.221	0.235	0.005	-0.150	0.159
India	Madhya Pradesh-Gwalior	114	0.688	0.217	-0.010	0.445	0.187	-0.008	0.382
India	Madhya Pradesh-Harda	113	0.908	-0.117	-0.488	0.254	-0.039	-0.163	0.085
India	Madhya Pradesh-Hoshangabad	113	0.633	0.042	-0.181	0.264	0.039	-0.168	0.246
India	Madhya Pradesh-Indore	106	0.742	-0.163	-0.415	0.088	-0.125	-0.318	0.067
India	Madhya Pradesh-Jabalpur	15	0.873	0.095	-0.844	1.035	0.042	-0.374	0.458
India	Madhya Pradesh-Jhabua	157	0.884	0.119	-0.164	0.401	0.049	-0.068	0.165
India	Madhya Pradesh-Katni	70	0.828	-0.162	-0.524	0.200	-0.092	-0.298	0.114
India	Madhya Pradesh-Khandwa (East Nimar)	57	0.863	0.149	-0.293	0.591	0.070	-0.139	0.279
India	Madhya Pradesh-Khargone (West Nimar)	105	0.672	0.355	0.128	0.583	0.314	0.113	0.514
India	Madhya Pradesh-Mandla	112	0.855	0.170	-0.135	0.474	0.084	-0.067	0.235
India	Madhya Pradesh-Mandsaur	91	0.660	0.087	-0.165	0.340	0.078	-0.148	0.305
India	Madhya Pradesh-Morena	162	0.687	0.033	-0.160	0.226	0.028	-0.138	0.194
India	Madhya Pradesh-Narsimhapur	127	0.742	0.368	0.146	0.590	0.282	0.112	0.452
India	Madhya Pradesh-Neemuch	125	0.853	-0.009	-0.297	0.280	-0.004	-0.149	0.140
India	Madhya Pradesh-Panna	112	0.555	0.351	0.144	0.558	0.346	0.142	0.551
India	Madhya Pradesh-Raisen	38	0.714	0.774	0.443	1.106	0.632	0.361	0.903
India	Madhya Pradesh-Rajgarh	98	0.571	0.349	0.126	0.572	0.342	0.124	0.560
India	Madhya Pradesh-Ratlam	136	0.854	0.497	0.233	0.761	0.248	0.116	0.380
India	Madhya Pradesh-Rewa	119	0.595	-0.089	-0.302	0.123	-0.086	-0.291	0.119
India	Madhya Pradesh-Sagar	127	0.625	0.176	-0.031	0.383	0.165	-0.029	0.359
India	Madhya Pradesh-Satna	79	0.601	0.240	-0.018	0.498	0.230	-0.017	0.478
India	Madhya Pradesh-Sehore	102	0.541	0.089	-0.137	0.316	0.089	-0.136	0.313
India	Madhya Pradesh-Seoni	114	0.812	-0.154	-0.426	0.118	-0.094	-0.260	0.072
India	Madhya Pradesh-Shahdol	100	0.800	-0.011	-0.297	0.274	-0.007	-0.190	0.176
India	Madhya Pradesh-Shajapur	132	0.732	0.020	-0.204	0.244	0.016	-0.160	0.192
India	Madhya Pradesh-Sheopur	151	0.626	0.283	0.097	0.469	0.265	0.091	0.439
India	Madhya Pradesh-Shivpuri	115	0.581	0.019	-0.197	0.234	0.018	-0.192	0.228
India	Madhya Pradesh-Sidhi	124	0.735	-0.044	-0.276	0.188	-0.035	-0.215	0.146
India	Madhya Pradesh-Singrauli	69	0.522	-0.217	-0.489	0.055	-0.217	-0.488	0.055
India	Madhya Pradesh-Tikamgarh	59	0.699	-0.143	-0.467	0.182	-0.120	-0.393	0.153
India	Madhya Pradesh-Ujjain	117	0.787	0.057	-0.201	0.315	0.038	-0.134	0.211
India	Madhya Pradesh-Umaria	98	0.581	0.142	-0.090	0.374	0.138	-0.088	0.364
India	Madhya Pradesh-Vidisha	122	0.735	0.174	-0.058	0.406	0.136	-0.045	0.316
India	Maharashtra-Ahmadnagar	108	0.701	0.376	0.147	0.605	0.315	0.123	0.507
India	Maharashtra-Akola	127	0.691	0.052	-0.167	0.271	0.044	-0.143	0.231
India	Maharashtra-Amravati	73	0.827	-0.142	-0.495	0.211	-0.081	-0.284	0.121
India	Maharashtra-Aurangabad	112	0.449	-0.079	-0.295	0.138	-0.078	-0.292	0.136
India	Maharashtra-Bhandara	74	0.859	0.092	-0.290	0.474	0.045	-0.141	0.230
India	Maharashtra-Bid	102	0.700	0.250	0.008	0.492	0.210	0.007	0.413
India	Maharashtra-Buldana	89	0.773	0.177	-0.110	0.464	0.124	-0.077	0.326
India	Maharashtra-Chandrapur	63	0.898	-0.239	-0.714	0.235	-0.088	-0.262	0.086
India	Maharashtra-Dhule	108	0.386	0.132	-0.093	0.356	0.125	-0.088	0.338
India	Maharashtra-Gadchiroli	82	0.945	0.456	-0.091	1.004	0.094	-0.019	0.207
India	Maharashtra-Gondiya	83	0.819	-0.284	-0.605	0.036	-0.169	-0.359	0.021
India	Maharashtra-Hingoli	108	0.643	0.108	-0.120	0.337	0.099	-0.110	0.309
India	Maharashtra-Jalgaon	104	0.285	0.316	0.076	0.557	0.258	0.062	0.454
India	Maharashtra-Jalna	96	0.493	0.012	-0.221	0.246	0.012	-0.221	0.246
India	Maharashtra-Kolhapur	82	0.371	0.124	-0.136	0.385	0.116	-0.127	0.359
India	Maharashtra-Latur	98	0.727	0.244	-0.010	0.499	0.194	-0.008	0.396
India	Maharashtra-Mumbai	41	0.632	0.015	-0.360	0.391	0.014	-0.335	0.364
India	Maharashtra-Mumbai Suburban	40	0.313	-0.306	-0.690	0.078	-0.263	-0.593	0.067
India	Maharashtra-Nagpur	85	0.770	-0.183	-0.475	0.110	-0.129	-0.337	0.078

India	Maharashtra-Nanded	106	0.536	0.090	-0.132	0.312	0.089	-0.131	0.310
India	Maharashtra-Nandurbar	119	0.289	0.118	-0.112	0.348	0.097	-0.092	0.286
India	Maharashtra-Nashik	117	0.591	0.348	0.143	0.553	0.337	0.138	0.535
India	Maharashtra-Osmanabad	105	0.791	0.009	-0.265	0.284	0.006	-0.175	0.188
India	Maharashtra-Palghar	66	0.806	-0.384	-0.729	-0.039	-0.240	-0.456	-0.024
India	Maharashtra-Parbhani	139	0.315	0.197	-0.008	0.403	0.170	-0.007	0.347
India	Maharashtra-Pune	75	0.352	0.090	-0.187	0.366	0.082	-0.170	0.334
India	Maharashtra-Raigarh	66	0.829	-0.001	-0.376	0.374	-0.001	-0.214	0.213
India	Maharashtra-Ratnagiri	62	0.502	-0.173	-0.462	0.115	-0.173	-0.462	0.115
India	Maharashtra-Sangli	85	0.444	0.274	0.031	0.517	0.270	0.030	0.510
India	Maharashtra-Satara	77	0.834	0.043	-0.308	0.394	0.024	-0.171	0.218
India	Maharashtra-Sindhudurg	42	0.556	0.236	-0.117	0.588	0.233	-0.115	0.581
India	Maharashtra-Solapur	108	0.750	0.011	-0.243	0.265	0.008	-0.182	0.199
India	Maharashtra-Thane	72	0.497	0.022	-0.248	0.292	0.022	-0.248	0.292
India	Maharashtra-Wardha	77	0.779	0.502	0.208	0.795	0.345	0.143	0.548
India	Maharashtra-Washim	81	0.660	-0.078	-0.346	0.190	-0.070	-0.311	0.171
India	Maharashtra-Yavatmal	88	0.707	0.047	-0.221	0.315	0.039	-0.183	0.261
India	Manipur-Bishnupur	96	0.266	0.068	-0.195	0.332	0.053	-0.153	0.259
India	Manipur-Chandel	111	0.263	0.147	-0.098	0.391	0.114	-0.076	0.304
India	Manipur-Churachandpur	143	0.421	0.279	0.091	0.466	0.272	0.089	0.455
India	Manipur-Imphal East	107	0.271	0.268	0.025	0.511	0.212	0.020	0.404
India	Manipur-Imphal West	75	0.280	0.153	-0.140	0.445	0.123	-0.113	0.359
India	Manipur-Senapati	125	0.376	0.187	-0.022	0.395	0.175	-0.020	0.370
India	Manipur-Tamenglong	125	0.257	0.016	-0.218	0.249	0.012	-0.166	0.190
India	Manipur-Thoubal	110	0.408	0.146	-0.074	0.365	0.141	-0.072	0.353
India	Manipur-Ukhrul	129	0.201	0.372	0.130	0.614	0.239	0.084	0.394
India	Meghalaya-East Garo Hills	104	0.195	-0.283	-0.560	-0.006	-0.178	-0.352	-0.004
India	Meghalaya-East Jantia Hills	336	0.480	-0.069	-0.193	0.054	-0.069	-0.193	0.054
India	Meghalaya-East Khasi Hills	146	0.240	-0.002	-0.222	0.219	-0.001	-0.162	0.160
India	Meghalaya-North Garo Hills	77	0.090	-0.143	-0.597	0.312	-0.047	-0.196	0.103
India	Meghalaya-Ribhoi	203	0.208	0.198	0.004	0.393	0.131	0.002	0.259
India	Meghalaya-South Garo Hills	81	0.313	-0.233	-0.502	0.037	-0.200	-0.432	0.032
India	Meghalaya-South West Garo Hills	104	0.438	-0.393	-0.606	-0.181	-0.387	-0.597	-0.178
India	Meghalaya-South West Khasi Hills	312	0.452	-0.097	-0.226	0.031	-0.096	-0.224	0.031
India	Meghalaya-West Garo Hills	88	0.289	-0.534	-0.778	-0.289	-0.439	-0.640	-0.238
India	Meghalaya-West Jaintia Hills	256	0.481	-0.007	-0.149	0.136	-0.007	-0.148	0.135
India	Meghalaya-West Khasi Hills	360	0.393	0.129	0.007	0.250	0.123	0.006	0.239
India	Mizoram-Aizawl	87	0.529	0.128	-0.116	0.373	0.128	-0.116	0.371
India	Mizoram-Champhai	70	0.660	0.169	-0.118	0.456	0.152	-0.106	0.409
India	Mizoram-Kolasib	110	0.698	0.233	0.000	0.466	0.196	0.000	0.393
India	Mizoram-Lawngtlai	123	0.571	0.156	-0.050	0.362	0.152	-0.049	0.354
India	Mizoram-Lunglei	59	0.519	-0.068	-0.368	0.231	-0.068	-0.367	0.231
India	Mizoram-Mamit	101	0.577	0.399	0.182	0.615	0.389	0.178	0.600
India	Mizoram-Saiha	125	0.401	0.003	-0.205	0.211	0.003	-0.197	0.203
India	Mizoram-Serchhip	138	0.581	-0.049	-0.245	0.148	-0.047	-0.239	0.144
India	Nagaland-Dimapur	82	0.267	0.026	-0.259	0.312	0.021	-0.203	0.245
India	Nagaland-Kiphire	103	0.264	-0.048	-0.303	0.208	-0.037	-0.235	0.161
India	Nagaland-Kohima	62	0.617	-0.117	-0.416	0.182	-0.111	-0.393	0.172
India	Nagaland-Longleng	116	0.280	0.176	-0.057	0.410	0.142	-0.046	0.331
India	Nagaland-Mokokchung	62	0.682	0.350	0.049	0.651	0.304	0.043	0.565
India	Nagaland-Mon	84	0.437	0.301	0.058	0.545	0.297	0.057	0.536
India	Nagaland-Peren	115	0.335	0.150	-0.074	0.374	0.134	-0.066	0.333
India	Nagaland-Phek	107	0.416	0.204	-0.017	0.425	0.198	-0.016	0.413
India	Nagaland-Tuensang	136	0.328	-0.027	-0.235	0.181	-0.023	-0.207	0.160
India	Nagaland-Wokha	52	0.391	0.363	0.051	0.675	0.346	0.049	0.643
India	Nagaland-Zunheboto	75	0.342	0.023	-0.256	0.302	0.021	-0.230	0.272
India	Nct Of Delhi-Central	75	0.760	0.242	-0.063	0.546	0.176	-0.046	0.399
India	Nct Of Delhi-East	94	0.682	0.143	-0.109	0.394	0.124	-0.095	0.342
India	Nct Of Delhi-New Delhi	99	0.679	0.081	-0.165	0.326	0.070	-0.144	0.284
India	Nct Of Delhi-North	103	0.562	0.001	-0.226	0.227	0.001	-0.223	0.224
India	Nct Of Delhi-North East	99	0.813	0.398	0.114	0.682	0.242	0.069	0.414
India	Nct Of Delhi-North West	90	0.815	-0.242	-0.548	0.065	-0.146	-0.331	0.039
India	Nct Of Delhi-Shahdara	91	0.688	-0.098	-0.356	0.160	-0.084	-0.306	0.137

India	Nct Of Delhi-South	81	0.817	0.187	-0.139	0.514	0.112	-0.083	0.308
India	Nct Of Delhi-South East	80	0.718	0.091	-0.193	0.375	0.074	-0.156	0.303
India	Nct Of Delhi-South West	83	0.852	0.356	0.010	0.701	0.179	0.005	0.354
India	Nct Of Delhi-West	115	0.686	0.292	0.069	0.515	0.252	0.060	0.444
India	Odisha-Anugul	97	0.822	0.268	-0.031	0.566	0.157	-0.018	0.332
India	Odisha-Balangir	94	0.936	0.773	0.318	1.227	0.186	0.077	0.295
India	Odisha-Baleshwar	77	0.834	0.166	-0.183	0.515	0.092	-0.101	0.285
India	Odisha-Bargarh	81	0.947	-0.301	-0.863	0.262	-0.061	-0.174	0.053
India	Odisha-Baudh	99	0.878	0.301	-0.045	0.647	0.129	-0.019	0.277
India	Odisha-Bhadrak	100	0.811	0.129	-0.162	0.420	0.079	-0.099	0.258
India	Odisha-Cuttack	77	0.852	-0.124	-0.490	0.243	-0.063	-0.248	0.123
India	Odisha-Debagarh	67	0.881	0.484	0.066	0.901	0.202	0.028	0.377
India	Odisha-Dhenkanal	82	0.854	-0.110	-0.468	0.248	-0.055	-0.233	0.123
India	Odisha-Gajapati	104	0.866	0.062	-0.266	0.391	0.029	-0.124	0.182
India	Odisha-Ganjam	97	0.883	0.476	0.129	0.824	0.197	0.053	0.342
India	Odisha-Jagatsinghapur	72	0.856	0.535	0.171	0.898	0.264	0.084	0.444
India	Odisha-Jajapur	86	0.805	0.086	-0.225	0.396	0.054	-0.142	0.249
India	Odisha-Jharsuguda	74	0.966	0.122	-0.613	0.856	0.016	-0.081	0.113
India	Odisha-Kalahandi	100	0.858	-0.138	-0.464	0.188	-0.067	-0.226	0.092
India	Odisha-Kandhamal	142	0.917	0.237	-0.107	0.580	0.072	-0.033	0.178
India	Odisha-Kendrapara	79	0.792	-0.012	-0.330	0.305	-0.008	-0.218	0.201
India	Odisha-Kendujhar	95	0.695	0.269	0.020	0.518	0.228	0.017	0.439
India	Odisha-Khordha	82	0.907	-0.324	-0.753	0.106	-0.109	-0.254	0.036
India	Odisha-Koraput	111	0.943	0.042	-0.427	0.512	0.009	-0.091	0.109
India	Odisha-Malkangiri	141	0.869	0.482	0.209	0.756	0.219	0.095	0.343
India	Odisha-Mayurbhanj	92	0.706	0.366	0.115	0.616	0.303	0.095	0.512
India	Odisha-Nabarangapur	146	0.911	-0.019	-0.351	0.313	-0.006	-0.113	0.101
India	Odisha-Nayagarh	90	0.926	-0.115	-0.577	0.346	-0.031	-0.157	0.094
India	Odisha-Nuapada	110	0.928	0.507	0.097	0.918	0.135	0.026	0.245
India	Odisha-Puri	73	0.943	0.073	-0.505	0.652	0.016	-0.109	0.140
India	Odisha-Rayagada	134	0.853	0.195	-0.081	0.471	0.098	-0.040	0.237
India	Odisha-Sambalpur	81	0.941	-0.183	-0.723	0.357	-0.040	-0.160	0.079
India	Odisha-Subarnapur	94	0.905	-0.227	-0.627	0.174	-0.078	-0.215	0.060
India	Odisha-Sundargarh	63	0.758	0.169	-0.166	0.505	0.124	-0.122	0.371
India	Puducherry-Karaikal	74	0.785	0.192	-0.129	0.514	0.130	-0.087	0.346
India	Puducherry-Mahe	55	0.944	0.314	-0.357	0.984	0.066	-0.076	0.208
India	Puducherry-Puducherry	70	0.851	0.015	-0.370	0.401	0.008	-0.188	0.203
India	Puducherry-Yanam	74	0.731	0.009	-0.292	0.309	0.007	-0.230	0.243
India	Punjab-Amritsar	90	0.484	-0.092	-0.333	0.148	-0.092	-0.332	0.148
India	Punjab-Barnala	73	0.621	0.105	-0.170	0.381	0.099	-0.160	0.359
India	Punjab-Bathinda	73	0.469	-0.043	-0.312	0.226	-0.043	-0.311	0.225
India	Punjab-Faridkot	85	0.555	0.050	-0.200	0.299	0.049	-0.198	0.296
India	Punjab-Fatehgarh Sahib	89	0.794	0.499	0.218	0.780	0.326	0.142	0.510
India	Punjab-Fazilka	92	0.525	0.164	-0.072	0.400	0.164	-0.072	0.399
India	Punjab-Firozpur	112	0.600	0.223	0.007	0.440	0.214	0.007	0.422
India	Punjab-Gurdaspur	85	0.547	0.217	-0.028	0.462	0.215	-0.028	0.458
India	Punjab-Hoshiarpur	72	0.726	0.036	-0.268	0.339	0.028	-0.213	0.269
India	Punjab-Jalandhar	76	0.780	0.145	-0.171	0.460	0.099	-0.118	0.316
India	Punjab-Kapurthala	90	0.552	0.245	0.008	0.482	0.242	0.008	0.477
India	Punjab-Ludhiana	75	0.586	0.184	-0.082	0.449	0.178	-0.079	0.436
India	Punjab-Mansa	103	0.643	-0.072	-0.306	0.163	-0.066	-0.281	0.150
India	Punjab-Moga	92	0.659	0.249	0.003	0.496	0.224	0.003	0.445
India	Punjab-Muktsar	82	0.551	0.342	0.099	0.585	0.339	0.098	0.579
India	Punjab-Pathankot	84	0.535	0.064	-0.186	0.314	0.064	-0.185	0.313
India	Punjab-Patiala	89	0.524	0.217	-0.022	0.455	0.216	-0.022	0.454
India	Punjab-Rupnagar	77	0.658	0.127	-0.146	0.401	0.115	-0.132	0.361
India	Punjab-Sahibzada Ajit Singh Nagar	85	0.782	-0.268	-0.564	0.027	-0.183	-0.384	0.019
India	Punjab-Sangrur	81	0.754	0.235	-0.056	0.527	0.175	-0.041	0.390
India	Punjab-Shahid Bhagat Singh Nagar	83	0.905	0.107	-0.322	0.536	0.037	-0.110	0.184
India	Punjab-Tarn Taran	90	0.576	0.306	0.070	0.541	0.299	0.069	0.529
India	Rajasthan-Ajmer	90	0.432	-0.052	-0.296	0.191	-0.052	-0.290	0.187
India	Rajasthan-Alwar	153	0.403	0.256	0.073	0.439	0.247	0.070	0.423
India	Rajasthan-Banswara	131	0.738	0.327	0.107	0.546	0.253	0.083	0.423

India	Rajasthan-Baran	157	0.792	0.082	-0.142	0.305	0.054	-0.094	0.201
India	Rajasthan-Barmer	176	0.831	0.517	0.301	0.733	0.290	0.169	0.411
India	Rajasthan-Bharatpur	161	0.382	0.053	-0.131	0.238	0.050	-0.124	0.224
India	Rajasthan-Bhilwara	122	0.611	-0.116	-0.327	0.094	-0.111	-0.311	0.090
India	Rajasthan-Bikaner	180	0.555	-0.029	-0.199	0.142	-0.028	-0.197	0.140
India	Rajasthan-Bundi	139	0.710	0.088	-0.124	0.301	0.073	-0.102	0.248
India	Rajasthan-Chittaurgarh	117	0.829	0.303	0.028	0.577	0.172	0.016	0.328
India	Rajasthan-Churu	157	0.532	0.142	-0.039	0.322	0.141	-0.039	0.321
India	Rajasthan-Dausa	143	0.679	0.012	-0.192	0.216	0.010	-0.168	0.188
India	Rajasthan-Dhaulpur	177	0.647	0.033	-0.146	0.211	0.030	-0.134	0.193
India	Rajasthan-Dungarpur	122	0.732	-0.100	-0.333	0.132	-0.079	-0.261	0.104
India	Rajasthan-Ganganagar	117	0.570	0.193	-0.017	0.403	0.189	-0.017	0.395
India	Rajasthan-Hanumangarh	124	0.555	0.081	-0.124	0.287	0.080	-0.123	0.284
India	Rajasthan-Jaipur	125	0.679	-0.304	-0.516	-0.092	-0.265	-0.450	-0.081
India	Rajasthan-Jaisalmer	187	0.749	0.330	0.144	0.516	0.248	0.108	0.388
India	Rajasthan-Jalor	150	0.707	0.005	-0.199	0.209	0.004	-0.165	0.174
India	Rajasthan-Jhalawar	128	0.759	0.366	0.139	0.593	0.268	0.102	0.434
India	Rajasthan-Jhunjhunun	122	0.607	-0.197	-0.405	0.012	-0.188	-0.387	0.011
India	Rajasthan-Jodhpur	193	0.771	0.095	-0.100	0.289	0.067	-0.070	0.204
India	Rajasthan-Karauli	142	0.606	0.279	0.089	0.470	0.267	0.085	0.448
India	Rajasthan-Kota	113	0.719	0.187	-0.049	0.423	0.151	-0.040	0.342
India	Rajasthan-Nagaur	153	0.746	0.134	-0.076	0.345	0.102	-0.058	0.261
India	Rajasthan-Pali	129	0.848	0.458	0.190	0.726	0.236	0.098	0.374
India	Rajasthan-Pratapgarh	138	0.736	0.159	-0.060	0.377	0.123	-0.046	0.293
India	Rajasthan-Rajsamand	115	0.424	0.105	-0.110	0.319	0.102	-0.107	0.312
India	Rajasthan-Sawai Madhopur	135	0.444	0.097	-0.099	0.294	0.096	-0.098	0.290
India	Rajasthan-Sikar	155	0.644	0.021	-0.170	0.212	0.020	-0.156	0.195
India	Rajasthan-Sirohi	123	0.510	0.014	-0.191	0.220	0.014	-0.191	0.220
India	Rajasthan-Tonk	109	0.546	-0.013	-0.232	0.207	-0.013	-0.230	0.205
India	Rajasthan-Udaipur	124	0.766	-0.049	-0.291	0.193	-0.035	-0.208	0.138
India	Sikkim-East District	50	0.299	0.011	-0.345	0.368	0.009	-0.290	0.308
India	Sikkim-North District	52	0.387	-0.090	-0.417	0.238	-0.085	-0.396	0.225
India	Sikkim-South District	55	0.580	-0.082	-0.396	0.232	-0.080	-0.386	0.226
India	Sikkim-West District	56	0.907	0.237	-0.290	0.764	0.080	-0.098	0.257
India	Tamil Nadu-Ariyalur	67	0.920	-0.841	-1.316	-0.366	-0.248	-0.388	-0.108
India	Tamil Nadu-Chennai	44	0.882	0.236	-0.299	0.772	0.099	-0.125	0.322
India	Tamil Nadu-Coimbatore	55	0.809	0.201	-0.190	0.592	0.124	-0.118	0.367
India	Tamil Nadu-Cuddalore	73	0.733	0.231	-0.068	0.530	0.181	-0.053	0.415
India	Tamil Nadu-Dharmapuri	76	0.737	-0.107	-0.405	0.190	-0.083	-0.314	0.148
India	Tamil Nadu-Dindigul	89	0.881	0.071	-0.303	0.445	0.030	-0.127	0.187
India	Tamil Nadu-Erode	73	0.881	0.221	-0.190	0.631	0.092	-0.079	0.264
India	Tamil Nadu-Kancheepuram	64	0.631	-0.190	-0.484	0.104	-0.177	-0.451	0.097
India	Tamil Nadu-Kanniyakumari	61	0.769	-0.388	-0.723	-0.053	-0.276	-0.514	-0.037
India	Tamil Nadu-Karur	58	0.644	-0.111	-0.426	0.203	-0.102	-0.390	0.186
India	Tamil Nadu-Krishnagiri	97	0.761	-0.042	-0.314	0.230	-0.031	-0.229	0.167
India	Tamil Nadu-Madurai	72	0.689	-0.005	-0.297	0.288	-0.004	-0.254	0.246
India	Tamil Nadu-Nagapattinam	67	0.919	0.022	-0.493	0.537	0.006	-0.146	0.159
India	Tamil Nadu-Namakkal	62	0.690	-0.132	-0.446	0.182	-0.113	-0.382	0.156
India	Tamil Nadu-Perambalur	62	0.858	-0.158	-0.575	0.259	-0.077	-0.280	0.126
India	Tamil Nadu-Pudukkottai	85	0.734	0.027	-0.254	0.308	0.021	-0.198	0.240
India	Tamil Nadu-Ramanathapuram	101	0.872	-0.128	-0.468	0.212	-0.057	-0.208	0.094
India	Tamil Nadu-Salem	47	0.684	0.027	-0.336	0.390	0.023	-0.290	0.337
India	Tamil Nadu-Sivaganga	84	0.848	0.222	-0.123	0.567	0.114	-0.063	0.292
India	Tamil Nadu-Thanjavur	57	0.661	-0.158	-0.478	0.161	-0.142	-0.428	0.144
India	Tamil Nadu-The Nilgiris	59	0.678	0.191	-0.126	0.508	0.167	-0.110	0.444
India	Tamil Nadu-Theni	106	0.948	-0.434	-0.927	0.059	-0.086	-0.183	0.012
India	Tamil Nadu-Thiruvallur	73	0.802	-0.067	-0.403	0.270	-0.042	-0.256	0.172
India	Tamil Nadu-Thiruvarur	56	0.912	0.095	-0.449	0.639	0.030	-0.144	0.204
India	Tamil Nadu-Thoothukkudi	76	0.865	-0.358	-0.734	0.019	-0.167	-0.343	0.009
India	Tamil Nadu-Tiruchirappalli	61	0.761	0.121	-0.223	0.465	0.088	-0.162	0.338
India	Tamil Nadu-Tirunelveli	65	0.843	-0.116	-0.507	0.275	-0.061	-0.268	0.146
India	Tamil Nadu-Tiruppur	50	0.834	0.299	-0.132	0.730	0.166	-0.073	0.404
India	Tamil Nadu-Tiruvannamalai	64	0.882	0.636	0.221	1.052	0.266	0.092	0.439

India	Tamil Nadu-Vellore	95	0.835	0.013	-0.303	0.329	0.007	-0.167	0.182
India	Tamil Nadu-Viluppuram	63	0.710	-0.085	-0.403	0.234	-0.070	-0.332	0.193
India	Tamil Nadu-Virudhunagar	72	0.677	-0.201	-0.486	0.084	-0.176	-0.426	0.074
India	Telangana-Adilabad	111	0.680	0.247	0.019	0.475	0.215	0.017	0.413
India	Telangana-Bhadradri Kothagudem	64	0.787	-0.363	-0.702	-0.024	-0.243	-0.471	-0.016
India	Telangana-Hyderabad	73	0.634	0.223	-0.051	0.497	0.207	-0.047	0.461
India	Telangana-Jagitial	73	0.729	-0.115	-0.415	0.186	-0.091	-0.329	0.147
India	Telangana-Jangoan	75	0.812	-0.077	-0.416	0.261	-0.047	-0.254	0.159
India	Telangana-Jayashankar Bhupalapally	52	0.829	-0.078	-0.502	0.347	-0.044	-0.285	0.197
India	Telangana-Jogulamba Gadwal	118	0.792	0.123	-0.134	0.381	0.081	-0.089	0.251
India	Telangana-Kamareddy	83	0.642	-0.260	-0.516	-0.004	-0.239	-0.474	-0.004
India	Telangana-Karimnagar	59	0.663	-0.305	-0.612	0.002	-0.273	-0.547	0.002
India	Telangana-Khammam	65	0.765	-0.038	-0.375	0.298	-0.028	-0.269	0.214
India	Telangana-Komaram Bheem Asifabad	80	0.704	-0.106	-0.386	0.173	-0.089	-0.322	0.144
India	Telangana-Mahabubabad	56	0.642	0.018	-0.303	0.339	0.016	-0.279	0.312
India	Telangana-Mahabubnagar	106	0.772	-0.136	-0.399	0.127	-0.096	-0.281	0.090
India	Telangana-Mancherial	65	0.681	0.187	-0.115	0.489	0.162	-0.100	0.425
India	Telangana-Medak	63	0.528	0.075	-0.214	0.365	0.075	-0.214	0.364
India	Telangana-Medchal-Malkajgiri	77	0.615	0.005	-0.263	0.274	0.005	-0.249	0.259
India	Telangana-Nagarkurnool	91	0.665	0.022	-0.232	0.276	0.020	-0.206	0.246
India	Telangana-Nalgonda	69	0.543	-0.029	-0.307	0.248	-0.029	-0.304	0.246
India	Telangana-Nirmal	79	0.768	0.116	-0.188	0.420	0.083	-0.134	0.300
India	Telangana-Nizamabad	98	0.729	-0.015	-0.275	0.245	-0.012	-0.217	0.193
India	Telangana-Peddapalli	53	0.688	0.162	-0.177	0.501	0.139	-0.152	0.430
India	Telangana-Rajanna Sircilla	73	0.737	-0.292	-0.589	0.006	-0.226	-0.457	0.004
India	Telangana-Ranga Reddy	93	0.738	-0.050	-0.320	0.219	-0.039	-0.247	0.170
India	Telangana-Sangareddy	93	0.494	0.050	-0.187	0.287	0.050	-0.187	0.287
India	Telangana-Siddipet	79	0.507	-0.238	-0.490	0.015	-0.238	-0.490	0.015
India	Telangana-Suryapet	49	0.682	-0.047	-0.401	0.307	-0.041	-0.348	0.266
India	Telangana-Vikarabad	93	0.710	0.067	-0.194	0.328	0.055	-0.160	0.270
India	Telangana-Wanaparthy	76	0.662	0.160	-0.116	0.435	0.143	-0.104	0.390
India	Telangana-Warangal Rural	59	0.652	0.156	-0.156	0.468	0.142	-0.141	0.425
India	Telangana-Warangal Urban	82	0.745	0.312	0.030	0.594	0.237	0.023	0.451
India	Telangana-Yadadri Bhuvanagiri	91	0.754	0.139	-0.138	0.416	0.103	-0.102	0.309
India	Tripura-Dhalai	104	0.654	0.477	0.261	0.694	0.432	0.236	0.628
India	Tripura-Gomati	78	0.212	0.135	-0.181	0.451	0.090	-0.121	0.301
India	Tripura-Khowai	65	0.509	0.205	-0.076	0.485	0.205	-0.076	0.485
India	Tripura-North Tripura	129	0.414	0.197	-0.004	0.397	0.191	-0.004	0.386
India	Tripura-Sepahijala	72	0.357	-0.066	-0.348	0.216	-0.060	-0.319	0.198
India	Tripura-South Tripura	77	0.338	0.095	-0.181	0.370	0.085	-0.162	0.331
India	Tripura-Unakoti	85	0.458	0.104	-0.145	0.352	0.103	-0.144	0.349
India	Tripura-West Tripura	61	0.666	-0.363	-0.661	-0.064	-0.322	-0.588	-0.057
India	Uttar Pradesh-Agra	166	0.633	-0.092	-0.275	0.091	-0.086	-0.255	0.084
India	Uttar Pradesh-Aligarh	149	0.684	0.020	-0.181	0.221	0.017	-0.156	0.191
India	Uttar Pradesh-Allahabad	138	0.422	0.313	0.123	0.502	0.305	0.120	0.490
India	Uttar Pradesh-Ambedkar Nagar	106	0.627	0.084	-0.145	0.313	0.079	-0.136	0.293
India	Uttar Pradesh-Amethi	167	0.617	-0.059	-0.240	0.122	-0.056	-0.227	0.115
India	Uttar Pradesh-Auraiya	141	0.592	-0.137	-0.331	0.057	-0.132	-0.319	0.055
India	Uttar Pradesh-Azamgarh	150	0.459	-0.009	-0.195	0.178	-0.009	-0.194	0.177
India	Uttar Pradesh-Baghpat	157	0.747	0.161	-0.046	0.369	0.122	-0.035	0.279
India	Uttar Pradesh-Bahraich	209	0.325	0.328	0.166	0.490	0.288	0.146	0.430
India	Uttar Pradesh-Ballia	116	0.408	0.203	-0.010	0.415	0.196	-0.009	0.401
India	Uttar Pradesh-Balrampur	207	0.461	-0.018	-0.176	0.141	-0.018	-0.175	0.140
India	Uttar Pradesh-Banda	131	0.423	0.021	-0.180	0.223	0.021	-0.176	0.218
India	Uttar Pradesh-Bara Banki	135	0.568	0.023	-0.175	0.221	0.023	-0.172	0.217
India	Uttar Pradesh-Bareilly	126	0.691	-0.165	-0.383	0.053	-0.141	-0.327	0.045
India	Uttar Pradesh-Basti	149	0.618	0.146	-0.045	0.337	0.138	-0.042	0.318
India	Uttar Pradesh-Bijnor	134	0.833	0.053	-0.211	0.317	0.030	-0.117	0.176
India	Uttar Pradesh-Budaun	169	0.674	0.026	-0.160	0.213	0.023	-0.141	0.187
India	Uttar Pradesh-Bulandshahr	179	0.677	0.007	-0.175	0.189	0.006	-0.153	0.165
India	Uttar Pradesh-Chandauli	148	0.509	-0.007	-0.194	0.180	-0.007	-0.194	0.180
India	Uttar Pradesh-Chitrakoot	139	0.426	0.133	-0.061	0.327	0.130	-0.060	0.320
India	Uttar Pradesh-Deoria	146	0.422	-0.050	-0.240	0.141	-0.048	-0.234	0.138

India	Uttar Pradesh-Etah	160	0.543	-0.007	-0.188	0.174	-0.007	-0.186	0.172
India	Uttar Pradesh-Etawah	153	0.713	0.250	0.050	0.450	0.204	0.041	0.368
India	Uttar Pradesh-Faizabad	144	0.539	0.178	-0.010	0.366	0.177	-0.010	0.364
India	Uttar Pradesh-Farrukhabad	159	0.560	0.271	0.094	0.447	0.267	0.092	0.441
India	Uttar Pradesh-Fatehpur	136	0.467	0.050	-0.146	0.246	0.050	-0.145	0.245
India	Uttar Pradesh-Firozabad	149	0.628	0.082	-0.110	0.275	0.077	-0.103	0.257
India	Uttar Pradesh-Gautam Buddha Nagar	96	0.667	0.237	-0.006	0.480	0.211	-0.005	0.426
India	Uttar Pradesh-Ghaziabad	84	0.677	0.028	-0.239	0.295	0.025	-0.209	0.258
India	Uttar Pradesh-Ghazipur	151	0.517	0.029	-0.156	0.214	0.029	-0.156	0.214
India	Uttar Pradesh-Gonda	154	0.517	0.230	0.050	0.410	0.230	0.050	0.409
India	Uttar Pradesh-Gorakhpur	153	0.562	0.021	-0.164	0.207	0.021	-0.162	0.203
India	Uttar Pradesh-Hamirpur	144	0.645	0.060	-0.139	0.258	0.055	-0.127	0.236
India	Uttar Pradesh-Hapur	194	0.795	-0.084	-0.286	0.118	-0.055	-0.186	0.077
India	Uttar Pradesh-Hardoi	150	0.471	0.001	-0.185	0.187	0.001	-0.185	0.187
India	Uttar Pradesh-Jalaun	132	0.548	0.083	-0.116	0.282	0.082	-0.115	0.279
India	Uttar Pradesh-Jaunpur	162	0.481	0.058	-0.121	0.237	0.058	-0.121	0.237
India	Uttar Pradesh-Jhansi	131	0.436	-0.065	-0.266	0.135	-0.064	-0.262	0.133
India	Uttar Pradesh-Jyotiba Phule Nagar	194	0.691	0.171	-0.004	0.346	0.146	-0.004	0.295
India	Uttar Pradesh-Kannauj	169	0.461	0.089	-0.086	0.264	0.088	-0.086	0.262
India	Uttar Pradesh-Kanpur Dehat	147	0.470	0.062	-0.126	0.250	0.062	-0.126	0.249
India	Uttar Pradesh-Kanpur Nagar	104	0.566	-0.158	-0.382	0.066	-0.155	-0.375	0.065
India	Uttar Pradesh-Kanshiram Nagar	186	0.609	0.004	-0.167	0.175	0.004	-0.159	0.167
India	Uttar Pradesh-Kaushambi	159	0.538	-0.107	-0.287	0.073	-0.106	-0.286	0.073
India	Uttar Pradesh-Kheri	175	0.617	0.110	-0.066	0.287	0.104	-0.062	0.271
India	Uttar Pradesh-Kushinagar	179	0.542	0.247	0.080	0.414	0.245	0.080	0.411
India	Uttar Pradesh-Lalitpur	179	0.500	0.001	-0.169	0.171	0.001	-0.169	0.171
India	Uttar Pradesh-Lucknow	94	0.621	0.140	-0.102	0.381	0.132	-0.096	0.359
India	Uttar Pradesh-Mahamaya Nagar	159	0.559	0.053	-0.129	0.235	0.052	-0.127	0.231
India	Uttar Pradesh-Maharajganj	147	0.558	-0.002	-0.191	0.188	-0.001	-0.188	0.185
India	Uttar Pradesh-Mahoba	162	0.569	-0.024	-0.205	0.156	-0.024	-0.201	0.153
India	Uttar Pradesh-Mainpuri	169	0.631	0.172	-0.008	0.351	0.160	-0.007	0.327
India	Uttar Pradesh-Mathura	165	0.631	-0.040	-0.223	0.144	-0.037	-0.208	0.134
India	Uttar Pradesh-Mau	136	0.410	-0.031	-0.230	0.167	-0.030	-0.223	0.162
India	Uttar Pradesh-Meerut	169	0.812	0.315	0.096	0.534	0.192	0.059	0.326
India	Uttar Pradesh-Mirzapur	160	0.583	0.058	-0.125	0.240	0.056	-0.121	0.234
India	Uttar Pradesh-Moradabad	150	0.715	-0.013	-0.219	0.193	-0.011	-0.179	0.157
India	Uttar Pradesh-Muzaffarnagar	130	0.713	0.225	0.007	0.442	0.184	0.006	0.362
India	Uttar Pradesh-Pilibhit	117	0.803	-0.112	-0.376	0.153	-0.070	-0.238	0.097
India	Uttar Pradesh-Pratapgarh	160	0.563	0.225	0.047	0.403	0.222	0.046	0.397
India	Uttar Pradesh-Rae Bareli	132	0.629	0.037	-0.168	0.242	0.035	-0.157	0.226
India	Uttar Pradesh-Rampur	129	0.707	-0.022	-0.242	0.199	-0.018	-0.201	0.165
India	Uttar Pradesh-Saharanpur	142	0.880	0.124	-0.169	0.418	0.053	-0.072	0.177
India	Uttar Pradesh-Sambhal	193	0.756	0.216	0.027	0.404	0.159	0.020	0.298
India	Uttar Pradesh-Sant Kabir Nagar	170	0.641	0.045	-0.137	0.227	0.041	-0.126	0.209
India	Uttar Pradesh-Sant Ravidas Nagar	197	0.414	0.059	-0.105	0.223	0.057	-0.102	0.217
India	Uttar Pradesh-Shahjahanpur	190	0.732	0.091	-0.095	0.277	0.071	-0.075	0.217
India	Uttar Pradesh-Shamli	159	0.860	-0.034	-0.294	0.227	-0.016	-0.141	0.109
India	Uttar Pradesh-Shrawasti	229	0.573	0.096	-0.055	0.248	0.094	-0.054	0.242
India	Uttar Pradesh-Siddharthnagar	222	0.603	0.085	-0.070	0.241	0.082	-0.067	0.231
India	Uttar Pradesh-Sitapur	169	0.597	0.019	-0.160	0.197	0.018	-0.154	0.190
India	Uttar Pradesh-Sonbhadra	160	0.498	0.063	-0.116	0.243	0.063	-0.116	0.243
India	Uttar Pradesh-Sultanpur	146	0.579	0.118	-0.072	0.308	0.115	-0.070	0.300
India	Uttar Pradesh-Unnao	104	0.432	0.052	-0.174	0.278	0.051	-0.171	0.273
India	Uttar Pradesh-Varanasi	144	0.548	0.016	-0.174	0.207	0.016	-0.173	0.205
India	Uttarakhand-Almora	63	0.832	0.185	-0.200	0.570	0.103	-0.112	0.318
India	Uttarakhand-Bageshwar	90	0.757	-0.072	-0.352	0.209	-0.053	-0.259	0.154
India	Uttarakhand-Chamoli	90	0.767	0.033	-0.252	0.318	0.023	-0.181	0.227
India	Uttarakhand-Champawat	108	0.846	0.341	0.044	0.639	0.178	0.023	0.333
India	Uttarakhand-Dehradun	81	0.750	-0.098	-0.391	0.195	-0.074	-0.294	0.146
India	Uttarakhand-Garhwal	60	0.706	0.081	-0.244	0.407	0.068	-0.203	0.338
India	Uttarakhand-Hardwar	96	0.666	0.322	0.084	0.561	0.287	0.074	0.499
India	Uttarakhand-Nainital	105	0.780	0.475	0.222	0.728	0.326	0.152	0.499
India	Uttarakhand-Pithoragarh	87	0.824	0.121	-0.200	0.443	0.070	-0.116	0.257

India	Uttarakhand-Rudraprayag	109	0.852	0.087	-0.221	0.395	0.044	-0.111	0.199
India	Uttarakhand-Tehri Garhwal	91	0.788	0.585	0.318	0.853	0.391	0.212	0.569
India	Uttarakhand-Udham Singh Nagar	126	0.728	-0.115	-0.342	0.113	-0.091	-0.271	0.089
India	Uttarakhand-Uttarkashi	106	0.729	0.114	-0.135	0.362	0.090	-0.107	0.287
India	West Bengal-Bankura	84	0.786	0.115	-0.189	0.418	0.077	-0.127	0.281
India	West Bengal-Birbhum	114	0.792	0.008	-0.255	0.271	0.005	-0.168	0.179
India	West Bengal-Dakshin Dinajpur	97	0.910	-0.129	-0.533	0.275	-0.042	-0.175	0.091
India	West Bengal-Darjiling	88	0.849	0.172	-0.166	0.511	0.088	-0.085	0.262
India	West Bengal-Haora	84	0.713	-0.051	-0.327	0.225	-0.042	-0.268	0.184
India	West Bengal-Hugli	75	0.685	0.235	-0.045	0.515	0.203	-0.039	0.444
India	West Bengal-Jalpaiguri	88	0.793	-0.331	-0.624	-0.038	-0.217	-0.410	-0.025
India	West Bengal-Koch Bihar	118	0.839	0.231	-0.052	0.514	0.125	-0.028	0.277
India	West Bengal-Kolkata	54	0.713	-0.340	-0.674	-0.005	-0.278	-0.552	-0.004
India	West Bengal-Maldah	137	0.744	0.068	-0.154	0.291	0.052	-0.118	0.222
India	West Bengal-Murshidabad	124	0.691	-0.209	-0.428	0.010	-0.179	-0.365	0.008
India	West Bengal-Nadia	77	0.812	0.182	-0.150	0.514	0.111	-0.092	0.314
India	West Bengal-North Twenty Four Parganas	77	0.928	0.545	0.056	1.034	0.146	0.015	0.278
India	West Bengal-Paschim Barddhaman	98	0.618	-0.035	-0.273	0.202	-0.033	-0.258	0.191
India	West Bengal-Paschim Medinipur	96	0.725	0.280	0.024	0.535	0.223	0.020	0.427
India	West Bengal-Purba Barddhaman	90	0.825	-0.136	-0.453	0.180	-0.078	-0.261	0.104
India	West Bengal-Purba Medinipur	93	0.643	-0.148	-0.394	0.098	-0.136	-0.362	0.090
India	West Bengal-Puruliya	106	0.713	-0.052	-0.297	0.193	-0.042	-0.243	0.158
India	West Bengal-South Twenty Four Parganas	95	0.678	0.366	0.126	0.606	0.320	0.110	0.529
India	West Bengal-Uttar Dinajpur	149	0.800	-0.026	-0.259	0.207	-0.017	-0.166	0.133
Indonesia	Bali	85	0.843	0.332	-0.002	0.665	0.176	-0.001	0.352
Indonesia	Bangka Belitung	90	0.803	-0.033	-0.336	0.270	-0.021	-0.213	0.171
Indonesia	Banten	186	0.559	0.251	0.087	0.415	0.247	0.086	0.409
Indonesia	Bengkulu	90	0.589	0.061	-0.184	0.306	0.059	-0.178	0.296
Indonesia	Central Java	357	0.825	0.023	-0.135	0.181	0.013	-0.078	0.105
Indonesia	Central Kalimantan	69	0.492	0.336	0.072	0.601	0.336	0.072	0.601
Indonesia	Central Sulawesi	157	0.644	0.466	0.291	0.641	0.427	0.267	0.588
Indonesia	Di Aceh	318	0.393	0.183	0.054	0.312	0.175	0.051	0.298
Indonesia	Di Yogyakarta	60	0.394	0.036	-0.268	0.340	0.035	-0.256	0.325
Indonesia	Dki Jakarta	189	0.681	-0.166	-0.343	0.011	-0.144	-0.298	0.010
Indonesia	East Java	371	0.759	0.140	0.003	0.277	0.102	0.002	0.203
Indonesia	East Kalimantan	174	0.766	0.028	-0.176	0.231	0.020	-0.126	0.166
Indonesia	East Nusa Tenggara	356	0.769	0.251	0.110	0.392	0.178	0.078	0.279
Indonesia	Gorontalo	76	0.738	0.185	-0.111	0.481	0.143	-0.086	0.372
Indonesia	Jambi	76	0.665	0.064	-0.214	0.342	0.057	-0.191	0.305
Indonesia	Lampung	141	0.766	-0.248	-0.471	-0.024	-0.177	-0.338	-0.017
Indonesia	Maluku	293	0.616	0.228	0.094	0.362	0.216	0.089	0.343
Indonesia	North Kalimantan	97	0.792	0.180	-0.104	0.464	0.119	-0.068	0.306
Indonesia	North Maluku	147	0.522	0.230	0.044	0.416	0.230	0.044	0.416
Indonesia	North Sulawesi	65	0.741	0.218	-0.103	0.538	0.167	-0.079	0.414
Indonesia	North Sumatera	327	0.628	0.209	0.081	0.337	0.195	0.076	0.315
Indonesia	Papua	100	0.534	0.223	-0.002	0.448	0.222	-0.002	0.446
Indonesia	Riau	151	0.560	0.196	0.011	0.380	0.193	0.011	0.374
Indonesia	Riau Islands	127	0.725	0.150	-0.075	0.375	0.120	-0.060	0.299
Indonesia	South Kalimantan	104	0.864	-0.146	-0.471	0.179	-0.069	-0.222	0.084
Indonesia	South Sulawesi	193	0.728	0.137	-0.047	0.321	0.108	-0.037	0.254
Indonesia	South Sumatera	136	0.669	0.118	-0.089	0.325	0.104	-0.079	0.288
Indonesia	Southeast Sulawesi	201	0.772	0.148	-0.042	0.339	0.105	-0.030	0.239
Indonesia	West Java	598	0.613	0.104	0.009	0.199	0.098	0.008	0.189
Indonesia	West Kalimantan	133	0.703	0.014	-0.205	0.232	0.011	-0.171	0.194
Indonesia	West Nusa Tenggara	169	0.779	-0.104	-0.315	0.107	-0.071	-0.216	0.073
Indonesia	West Papua	91	0.631	0.648	0.439	0.857	0.604	0.409	0.798
Indonesia	West Sulawesi	211	0.620	0.093	-0.068	0.253	0.087	-0.064	0.239
Indonesia	West Sumatera	128	0.524	0.227	0.029	0.425	0.226	0.029	0.424
Jordan	Ajloun	315	0.837	0.114	-0.059	0.286	0.062	-0.032	0.157
Jordan	Amman	361	0.816	0.069	-0.085	0.223	0.041	-0.051	0.134
Jordan	Aqaba	232	0.753	0.087	-0.086	0.259	0.065	-0.064	0.193
Jordan	Balqa	210	0.776	0.001	-0.187	0.189	0.001	-0.130	0.131
Jordan	Irbid	308	0.789	0.013	-0.145	0.171	0.009	-0.097	0.114

Jordan	Jarash	314	0.834	0.009	-0.163	0.181	0.005	-0.090	0.100
Jordan	Karak	202	0.811	-0.110	-0.314	0.094	-0.068	-0.193	0.057
Jordan	Ma'an	232	0.653	0.301	0.150	0.453	0.273	0.136	0.411
Jordan	Madaba	262	0.777	0.142	-0.025	0.310	0.099	-0.018	0.215
Jordan	Mafraq	485	0.761	-0.156	-0.276	-0.036	-0.114	-0.201	-0.026
Jordan	Tafiela	291	0.856	0.220	0.032	0.408	0.109	0.016	0.201
Jordan	Zarqa	346	0.874	-0.056	-0.239	0.128	-0.025	-0.106	0.056
Kenya	Baringo	150	0.762	0.334	0.122	0.545	0.242	0.088	0.395
Kenya	Bomet	160	0.857	0.482	0.236	0.728	0.236	0.115	0.356
Kenya	Bungoma	196	0.744	-0.083	-0.272	0.107	-0.063	-0.207	0.081
Kenya	Busia	168	0.757	-0.020	-0.225	0.185	-0.015	-0.165	0.136
Kenya	Elgeyo Marakwet	145	0.879	0.288	0.002	0.575	0.123	0.001	0.245
Kenya	Embu	90	0.822	-0.065	-0.380	0.249	-0.038	-0.223	0.146
Kenya	Garissa	158	0.540	-0.081	-0.263	0.100	-0.081	-0.261	0.099
Kenya	Homa Bay	188	0.567	0.028	-0.140	0.196	0.027	-0.138	0.193
Kenya	Isiolo	174	0.789	-0.162	-0.372	0.048	-0.108	-0.248	0.032
Kenya	Kajiado	175	0.504	-0.005	-0.178	0.167	-0.005	-0.178	0.167
Kenya	Kakamega	184	0.662	-0.068	-0.245	0.109	-0.061	-0.219	0.098
Kenya	Kericho	145	0.676	-0.066	-0.268	0.136	-0.058	-0.234	0.119
Kenya	Kiambu	113	0.762	0.054	-0.199	0.307	0.040	-0.144	0.223
Kenya	Kilifi	184	0.768	-0.064	-0.263	0.135	-0.045	-0.187	0.096
Kenya	Kirinyaga	67	0.734	0.256	-0.056	0.567	0.200	-0.044	0.443
Kenya	Kisii	160	0.827	0.229	-0.007	0.464	0.131	-0.004	0.265
Kenya	Kisumu	147	0.772	0.228	0.006	0.450	0.161	0.004	0.317
Kenya	Kitui	132	0.698	0.258	0.046	0.469	0.217	0.039	0.396
Kenya	Kwale	132	0.843	0.160	-0.112	0.431	0.085	-0.059	0.228
Kenya	Laikipia	130	0.765	0.391	0.165	0.617	0.281	0.119	0.443
Kenya	Lamu	121	0.656	-0.034	-0.255	0.187	-0.031	-0.230	0.169
Kenya	Machakos	118	0.885	0.100	-0.229	0.430	0.041	-0.093	0.174
Kenya	Makueni	129	0.845	0.145	-0.131	0.422	0.076	-0.069	0.221
Kenya	Mandera	155	0.272	0.199	-0.004	0.402	0.158	-0.003	0.318
Kenya	Marsabit	144	0.772	0.255	0.031	0.479	0.180	0.022	0.338
Kenya	Meru	109	0.826	0.107	-0.181	0.395	0.062	-0.104	0.227
Kenya	Migori	217	0.397	-0.126	-0.282	0.031	-0.120	-0.270	0.030
Kenya	Mombasa	107	0.687	-0.207	-0.446	0.031	-0.179	-0.384	0.026
Kenya	Murang'a	98	0.742	0.289	0.031	0.546	0.221	0.024	0.418
Kenya	Nairobi	155	0.729	-0.033	-0.238	0.173	-0.026	-0.188	0.137
Kenya	Nakuru	164	0.777	0.029	-0.185	0.242	0.020	-0.128	0.168
Kenya	Nandi	154	0.932	0.435	0.077	0.793	0.110	0.020	0.201
Kenya	Narok	234	0.679	0.182	0.025	0.339	0.159	0.021	0.296
Kenya	Nyamira	116	0.913	0.117	-0.258	0.491	0.037	-0.082	0.157
Kenya	Nyandarua	96	0.781	0.211	-0.068	0.490	0.144	-0.046	0.335
Kenya	Nyeri	118	0.722	0.006	-0.228	0.241	0.005	-0.183	0.193
Kenya	Samburu	198	0.633	0.333	0.172	0.494	0.309	0.160	0.459
Kenya	Siaya	151	0.706	-0.010	-0.214	0.193	-0.009	-0.178	0.160
Kenya	Taita Taveta	90	0.828	0.450	0.145	0.755	0.257	0.083	0.431
Kenya	Tana River	209	0.630	-0.091	-0.253	0.072	-0.085	-0.236	0.067
Kenya	Tharaka-Nithi	97	0.881	-0.073	-0.432	0.286	-0.031	-0.181	0.119
Kenya	Trans-Nzoia	143	0.645	0.021	-0.179	0.222	0.019	-0.164	0.203
Kenya	Turkana	174	0.537	-0.006	-0.180	0.168	-0.006	-0.179	0.167
Kenya	Uasin Gishu	154	0.697	0.003	-0.197	0.203	0.002	-0.166	0.171
Kenya	Vihiga	134	0.905	0.108	-0.227	0.443	0.037	-0.078	0.153
Kenya	Wajir	191	0.456	0.130	-0.034	0.295	0.129	-0.034	0.293
Kenya	West Pokot	194	0.411	-0.006	-0.173	0.161	-0.006	-0.167	0.156
Lesotho	Berea	120	0.726	0.193	-0.038	0.424	0.154	-0.030	0.337
Lesotho	Butha-Buthe	96	0.680	0.234	-0.011	0.480	0.204	-0.010	0.418
Lesotho	Leribe	130	0.730	-0.132	-0.357	0.092	-0.104	-0.281	0.072
Lesotho	Mafeteng	79	0.742	0.035	-0.259	0.330	0.027	-0.198	0.253
Lesotho	Maseru	123	0.695	-0.015	-0.239	0.208	-0.013	-0.202	0.177
Lesotho	Mohale's Hoek	99	0.643	0.027	-0.213	0.267	0.025	-0.195	0.245
Lesotho	Mokhotlong	104	0.524	-0.019	-0.244	0.205	-0.019	-0.243	0.204
Lesotho	Qacha's Nek	78	0.784	0.130	-0.184	0.444	0.088	-0.125	0.301
Lesotho	Quthing	88	0.615	0.037	-0.214	0.287	0.035	-0.203	0.272

Lesotho	Thaba-Tseka	112	0.634	0.150	-0.072	0.373	0.140	-0.067	0.346
Liberia	Bomi	82	0.562	-0.085	-0.339	0.169	-0.083	-0.334	0.167
Liberia	Bong	163	0.439	0.087	-0.092	0.266	0.086	-0.091	0.262
Liberia	Gbarpolu	98	0.472	0.271	0.046	0.496	0.270	0.046	0.495
Liberia	Grand Bassa	145	0.335	0.222	0.025	0.419	0.197	0.022	0.373
Liberia	Grand Cape Mount	104	0.540	-0.063	-0.288	0.161	-0.063	-0.286	0.160
Liberia	Grand Gedeh	85	0.414	0.158	-0.092	0.407	0.153	-0.090	0.395
Liberia	Grand Kru	104	0.514	0.201	-0.020	0.421	0.200	-0.020	0.421
Liberia	Lofa	125	0.646	-0.051	-0.264	0.162	-0.047	-0.241	0.148
Liberia	Margibi	89	0.328	0.219	-0.035	0.473	0.193	-0.031	0.417
Liberia	Maryland	117	0.556	0.416	0.218	0.615	0.411	0.215	0.607
Liberia	Montserrado Incl. Monrovia	194	0.468	0.113	-0.050	0.276	0.112	-0.050	0.275
Liberia	Nimba	203	0.504	-0.019	-0.178	0.141	-0.019	-0.178	0.141
Liberia	River Cess	83	0.401	-0.017	-0.274	0.239	-0.016	-0.263	0.230
Liberia	River Gee	77	0.343	0.140	-0.133	0.413	0.126	-0.120	0.373
Liberia	Sinoe	102	0.310	0.306	0.069	0.544	0.262	0.059	0.465
Madagascar	Alaotra Mangoro	137	0.591	0.511	0.333	0.690	0.495	0.322	0.667
Madagascar	Amoron'i Mania	196	0.737	0.350	0.173	0.528	0.272	0.134	0.410
Madagascar	Analamanga Excluding Capital	139	0.732	-0.144	-0.361	0.073	-0.113	-0.284	0.057
Madagascar	Analanjirofo	149	0.556	0.334	0.154	0.514	0.330	0.153	0.508
Madagascar	Androy	273	0.331	0.088	-0.058	0.233	0.078	-0.051	0.207
Madagascar	Anosy	207	0.385	0.133	-0.028	0.295	0.126	-0.027	0.279
Madagascar	Antananarivo Capital	213	0.588	0.237	0.082	0.392	0.230	0.080	0.380
Madagascar	Atsimo Andrefana	217	0.307	0.069	-0.098	0.236	0.059	-0.084	0.201
Madagascar	Atsimo Atsinanana	207	0.440	0.351	0.199	0.502	0.346	0.196	0.495
Madagascar	Atsinanana	160	0.359	0.225	0.040	0.409	0.207	0.037	0.376
Madagascar	Betsiboka	179	0.336	0.167	-0.012	0.345	0.149	-0.011	0.308
Madagascar	Boeny	117	0.423	0.379	0.177	0.581	0.370	0.173	0.568
Madagascar	Bongolava	193	0.555	0.228	0.067	0.390	0.225	0.066	0.385
Madagascar	Diana	151	0.460	0.162	-0.022	0.346	0.161	-0.022	0.344
Madagascar	Haute Matsiatra	184	0.625	0.026	-0.147	0.199	0.025	-0.138	0.187
Madagascar	Ihorombe	211	0.419	0.194	0.038	0.350	0.189	0.037	0.341
Madagascar	Itasy	178	0.777	-0.034	-0.239	0.171	-0.024	-0.166	0.118
Madagascar	Melaky	157	0.352	0.423	0.245	0.602	0.386	0.224	0.549
Madagascar	Menabe	139	0.361	0.074	-0.127	0.275	0.069	-0.117	0.254
Madagascar	Sava	148	0.257	0.319	0.111	0.527	0.244	0.085	0.403
Madagascar	Sofia	140	0.378	0.476	0.293	0.658	0.447	0.276	0.618
Madagascar	Vakinankaratra	167	0.671	0.012	-0.175	0.200	0.011	-0.155	0.176
Madagascar	Vatovavy Fitovinany	144	0.417	0.145	-0.046	0.336	0.141	-0.045	0.327
Malawi	Balaka	196	0.696	0.156	-0.019	0.331	0.132	-0.016	0.281
Malawi	Blantyre	191	0.573	-0.158	-0.322	0.007	-0.154	-0.316	0.007
Malawi	Chikwawa	206	0.658	0.221	0.057	0.385	0.199	0.051	0.347
Malawi	Chiradzulu	161	0.793	0.026	-0.195	0.247	0.017	-0.128	0.163
Malawi	Chitipa	148	0.774	0.220	-0.001	0.441	0.154	-0.001	0.308
Malawi	Dedza	192	0.717	0.410	0.238	0.583	0.333	0.193	0.473
Malawi	Dowa	180	0.754	0.077	-0.119	0.274	0.057	-0.088	0.203
Malawi	Karonga	187	0.817	0.223	0.010	0.436	0.133	0.006	0.260
Malawi	Kasungu	208	0.777	-0.128	-0.316	0.061	-0.089	-0.219	0.042
Malawi	Likoma	134	0.829	-0.312	-0.568	-0.056	-0.177	-0.322	-0.032
Malawi	Lilongwe	251	0.745	0.157	-0.006	0.320	0.119	-0.005	0.244
Malawi	Machinga	238	0.669	0.057	-0.100	0.213	0.050	-0.088	0.189
Malawi	Mangochi	288	0.706	0.097	-0.050	0.243	0.080	-0.041	0.202
Malawi	Mchinji	237	0.759	0.049	-0.124	0.221	0.036	-0.090	0.162
Malawi	Mulanje	193	0.763	-0.062	-0.254	0.131	-0.044	-0.184	0.095
Malawi	Mwanza	161	0.896	0.322	0.032	0.612	0.120	0.012	0.228
Malawi	Mzimba	234	0.761	0.131	-0.042	0.304	0.095	-0.031	0.222
Malawi	Neno	208	0.832	-0.029	-0.240	0.182	-0.016	-0.134	0.102
Malawi	Nkhata Bay	184	0.652	0.084	-0.092	0.260	0.076	-0.083	0.236
Malawi	Nkhotakota	226	0.682	-0.025	-0.187	0.137	-0.022	-0.162	0.119
Malawi	Nsanje	198	0.642	-0.075	-0.243	0.094	-0.069	-0.223	0.086
Malawi	Ntcheu	221	0.710	0.129	-0.039	0.296	0.106	-0.032	0.244
Malawi	Ntchisi	187	0.655	0.168	-0.005	0.341	0.152	-0.005	0.308
Malawi	Phalombe	207	0.724	0.119	-0.057	0.295	0.095	-0.046	0.235

Malawi	Rumphi	182	0.815	0.294	0.081	0.507	0.177	0.049	0.306
Malawi	Salima	223	0.783	0.097	-0.087	0.281	0.066	-0.059	0.191
Malawi	Thyolo	177	0.768	0.111	-0.091	0.313	0.079	-0.065	0.223
Malawi	Zomba	205	0.736	0.099	-0.081	0.279	0.077	-0.063	0.216
Maldives	Central	154	0.822	0.103	-0.136	0.342	0.060	-0.080	0.201
Maldives	Male	87	0.802	-0.043	-0.351	0.265	-0.027	-0.223	0.168
Maldives	North	176	0.784	0.161	-0.046	0.368	0.109	-0.031	0.249
Maldives	North Central	207	0.751	0.042	-0.141	0.225	0.031	-0.105	0.168
Maldives	South	167	0.698	-0.072	-0.263	0.120	-0.060	-0.222	0.101
Maldives	South Central	228	0.754	0.125	-0.049	0.299	0.092	-0.037	0.222
Mali	Bamako	400	0.447	0.162	0.049	0.274	0.160	0.048	0.271
Mali	Gao	244	0.202	0.243	0.064	0.422	0.156	0.041	0.271
Mali	Kayes	416	0.348	0.177	0.062	0.293	0.161	0.056	0.266
Mali	Kidal	150	0.000	0.000	0.000	0.000	0.000	0.000	0.000
Mali	Koulikoro	448	0.389	0.032	-0.077	0.142	0.031	-0.074	0.135
Mali	Mopti	264	0.413	0.047	-0.095	0.189	0.045	-0.092	0.183
Mali	Segou	404	0.473	-0.071	-0.184	0.041	-0.071	-0.184	0.041
Mali	Sikasso	566	0.393	0.014	-0.083	0.112	0.014	-0.079	0.107
Mali	Tombouctou	332	0.361	-0.036	-0.166	0.094	-0.033	-0.153	0.086
Mauritania	Adrar	149	0.486	0.122	-0.064	0.307	0.122	-0.064	0.307
Mauritania	Assaba	323	0.351	-0.181	-0.312	-0.050	-0.165	-0.284	-0.046
Mauritania	Brakna	383	0.415	-0.207	-0.323	-0.091	-0.201	-0.314	-0.089
Mauritania	Dakhlet Nouadhibou	143	0.391	0.105	-0.090	0.299	0.100	-0.085	0.285
Mauritania	Eastern Basin Region	363	0.259	0.036	-0.100	0.171	0.027	-0.077	0.132
Mauritania	Gorgol	353	0.387	0.125	0.001	0.248	0.118	0.001	0.235
Mauritania	Guidimagha	528	0.448	-0.086	-0.185	0.013	-0.085	-0.183	0.013
Mauritania	North Nouakchott	173	0.388	-0.220	-0.394	-0.045	-0.209	-0.374	-0.043
Mauritania	South Nouakchott	153	0.249	0.138	-0.074	0.350	0.103	-0.056	0.261
Mauritania	Tagant	236	0.408	0.056	-0.094	0.207	0.054	-0.091	0.200
Mauritania	Tiris Zemour And Inchiri	190	0.392	0.030	-0.139	0.199	0.028	-0.133	0.189
Mauritania	Trarza	252	0.183	-0.048	-0.233	0.137	-0.029	-0.139	0.082
Mauritania	West Nouakchott	119	0.312	-0.041	-0.267	0.185	-0.035	-0.229	0.158
Mauritania	Western Basin Region	380	0.503	0.165	0.050	0.280	0.165	0.050	0.280
Myanmar	Ayeyarwaddy	108	0.396	-0.101	-0.324	0.123	-0.096	-0.310	0.118
Myanmar	Bago	85	0.548	0.271	0.028	0.513	0.268	0.028	0.509
Myanmar	Chin	147	0.550	0.211	0.026	0.397	0.209	0.025	0.393
Myanmar	Kachin	111	0.726	0.196	-0.045	0.436	0.156	-0.035	0.347
Myanmar	Kayah	131	0.796	0.357	0.118	0.597	0.232	0.077	0.388
Myanmar	Kayin	117	0.621	0.529	0.333	0.725	0.498	0.314	0.682
Myanmar	Magway	72	0.653	0.095	-0.188	0.378	0.086	-0.171	0.343
Myanmar	Mandalay	86	0.863	-0.014	-0.373	0.345	-0.007	-0.176	0.163
Myanmar	Mon	79	0.682	-0.056	-0.333	0.220	-0.049	-0.289	0.191
Myanmar	Naypyitaw	81	0.494	0.422	0.185	0.659	0.422	0.185	0.659
Myanmar	Rakhine	127	0.420	0.185	-0.019	0.389	0.180	-0.019	0.379
Myanmar	Sagaing	99	0.707	0.052	-0.200	0.304	0.043	-0.166	0.252
Myanmar	Shan	113	0.592	0.114	-0.104	0.331	0.110	-0.100	0.320
Myanmar	Taninthayi	119	0.493	0.307	0.105	0.510	0.307	0.105	0.510
Myanmar	Yangon	85	0.772	0.202	-0.091	0.495	0.142	-0.064	0.349
Nepal	Province 1	254	0.814	-0.036	-0.220	0.147	-0.022	-0.133	0.089
Nepal	Province 2	346	0.712	0.082	-0.053	0.216	0.067	-0.043	0.177
Nepal	Province 3	174	0.750	-0.009	-0.208	0.191	-0.007	-0.156	0.143
Nepal	Province 4	180	0.941	0.259	-0.099	0.618	0.058	-0.022	0.137
Nepal	Province 5	267	0.850	0.046	-0.149	0.240	0.023	-0.076	0.122
Nepal	Province 6	272	0.776	0.076	-0.089	0.241	0.053	-0.062	0.168
Nepal	Province 7	217	0.837	0.124	-0.085	0.332	0.067	-0.046	0.181
Nigeria	Abia	209	0.327	0.104	-0.063	0.271	0.091	-0.056	0.238
Nigeria	Adamawa	311	0.320	0.065	-0.072	0.203	0.057	-0.063	0.177
Nigeria	Akwa Ibom	179	0.357	0.091	-0.086	0.268	0.083	-0.079	0.246
Nigeria	Anambra	287	0.761	0.377	0.226	0.528	0.274	0.164	0.383
Nigeria	Bauchi	439	0.204	0.322	0.191	0.453	0.209	0.124	0.294
Nigeria	Bayelsa	201	0.122	0.141	-0.103	0.385	0.061	-0.044	0.166
Nigeria	Benue	297	0.283	0.212	0.068	0.356	0.172	0.055	0.289
Nigeria	Borno	355	0.212	0.298	0.154	0.442	0.200	0.103	0.296

Nigeria	Cross River	142	0.488	0.185	-0.004	0.374	0.185	-0.004	0.374
Nigeria	Delta	168	0.487	0.140	-0.035	0.314	0.140	-0.034	0.314
Nigeria	Ebonyi	318	0.476	0.134	0.008	0.261	0.134	0.008	0.260
Nigeria	Edo	139	0.564	0.131	-0.062	0.325	0.129	-0.061	0.320
Nigeria	Ekiti	164	0.399	0.330	0.155	0.504	0.316	0.149	0.483
Nigeria	Enugu	202	0.362	0.080	-0.086	0.246	0.074	-0.080	0.227
Nigeria	Fct-Abuja	241	0.470	0.238	0.094	0.381	0.237	0.094	0.380
Nigeria	Gombe	370	0.215	0.428	0.292	0.565	0.289	0.197	0.382
Nigeria	Imo	229	0.574	0.351	0.206	0.496	0.344	0.202	0.485
Nigeria	Jigawa	417	0.189	0.153	0.012	0.294	0.094	0.007	0.180
Nigeria	Kaduna	391	0.191	0.359	0.218	0.501	0.222	0.134	0.309
Nigeria	Kano	584	0.260	0.293	0.189	0.398	0.226	0.146	0.306
Nigeria	Katsina	449	0.187	0.163	0.027	0.300	0.100	0.017	0.183
Nigeria	Kebbi	353	0.040	0.518	0.215	0.821	0.080	0.033	0.127
Nigeria	Kogi	168	0.308	0.214	0.026	0.401	0.182	0.023	0.342
Nigeria	Kwara	215	0.306	0.568	0.418	0.718	0.483	0.356	0.610
Nigeria	Lagos	275	0.565	0.252	0.118	0.387	0.248	0.116	0.381
Nigeria	Nasarawa	251	0.290	0.167	0.010	0.323	0.137	0.008	0.266
Nigeria	Niger	354	0.189	0.387	0.239	0.536	0.238	0.146	0.329
Nigeria	Ogun	168	0.257	0.542	0.359	0.726	0.414	0.274	0.554
Nigeria	Ondo	162	0.535	0.387	0.218	0.556	0.385	0.217	0.553
Nigeria	Osun	172	0.319	0.003	-0.183	0.189	0.003	-0.159	0.164
Nigeria	Oyo	210	0.201	0.251	0.058	0.444	0.161	0.037	0.285
Nigeria	Plateau	258	0.458	0.230	0.091	0.369	0.229	0.091	0.367
Nigeria	Rivers	219	0.407	0.278	0.126	0.430	0.268	0.121	0.415
Nigeria	Sokoto	299	0.045	0.272	-0.042	0.586	0.047	-0.007	0.101
Nigeria	Taraba	310	0.236	0.164	0.013	0.314	0.118	0.009	0.227
Nigeria	Yobe	381	0.168	0.305	0.153	0.458	0.170	0.085	0.256
Nigeria	Zamfara	325	0.069	0.421	0.177	0.664	0.109	0.046	0.172
Pakistan	Azad, Jammu And Kashmir	449	0.771	0.211	0.086	0.337	0.149	0.061	0.238
Pakistan	Balochistan	456	0.358	0.325	0.218	0.431	0.298	0.200	0.397
Pakistan	Federally Administered Tribal Areas	364	0.306	0.095	-0.034	0.223	0.080	-0.029	0.190
Pakistan	Gilgit Baltistan	293	0.727	0.131	-0.017	0.280	0.104	-0.013	0.222
Pakistan	Islamabad (Ict)	271	0.744	0.115	-0.043	0.272	0.087	-0.033	0.208
Pakistan	Khyber Pakhtunkhwa	664	0.554	0.367	0.283	0.451	0.362	0.279	0.445
Pakistan	Punjab Excluding Islamabad (Ict)<br>Sindh	875	0.794	0.162	0.068	0.256	0.106	0.044	0.167
	Sindh								
Pakistan	Sindh	705	0.524	0.407	0.327	0.487	0.406	0.326	0.486
Philippines	Armm	289	0.204	0.123	-0.042	0.288	0.080	-0.027	0.187
Philippines	Bicol	251	0.741	0.030	-0.133	0.194	0.023	-0.102	0.149
Philippines	Cagayan Valley	142	0.624	0.118	-0.079	0.314	0.111	-0.074	0.295
Philippines	Calabarzon	193	0.730	0.069	-0.115	0.253	0.054	-0.091	0.199
Philippines	Caraga	207	0.765	0.262	0.079	0.444	0.188	0.057	0.320
Philippines	Central Luzon	264	0.637	0.224	0.081	0.367	0.207	0.075	0.339
Philippines	Central Visayas	153	0.726	-0.231	-0.434	-0.028	-0.184	-0.346	-0.022
Philippines	Cordillera Admin Region	218	0.767	0.329	0.152	0.506	0.235	0.109	0.362
Philippines	Davao Peninsula	197	0.862	0.297	0.066	0.528	0.141	0.031	0.251
Philippines	Eastern Visayas	254	0.795	0.090	-0.087	0.266	0.058	-0.056	0.173
Philippines	Ilocos	114	0.676	0.144	-0.083	0.371	0.126	-0.073	0.325
Philippines	Mimaropa	161	0.594	0.255	0.076	0.433	0.246	0.074	0.418
Philippines	National Capital Region	267	0.748	0.169	0.010	0.328	0.128	0.008	0.247
Philippines	Northern Mindanao	239	0.693	0.157	-0.001	0.315	0.134	-0.001	0.268
Philippines	Soccsksargen	185	0.425	0.311	0.148	0.474	0.304	0.145	0.464
Philippines	Western Visayas	241	0.650	0.436	0.293	0.579	0.397	0.266	0.527
Philippines	Zamboanga Peninsula	148	0.586	0.188	0.000	0.376	0.182	0.000	0.365
Rwanda	East	678	0.945	0.180	-0.010	0.370	0.038	-0.002	0.077
Rwanda	Kigali	307	0.961	0.148	-0.186	0.481	0.022	-0.028	0.072
Rwanda	North	423	0.926	0.059	-0.151	0.269	0.016	-0.042	0.074
Rwanda	South	623	0.971	0.232	-0.036	0.501	0.026	-0.004	0.057

Rwanda	West	698	0.940	0.107	-0.074	0.287	0.024	-0.017	0.065
Senegal	Center	700	0.796	-0.050	-0.156	0.056	-0.032	-0.101	0.037
Senegal	North	410	0.689	0.430	0.317	0.544	0.369	0.272	0.467
Senegal	South	681	0.662	0.218	0.127	0.308	0.195	0.114	0.276
Senegal	West	312	0.771	0.083	-0.070	0.236	0.059	-0.049	0.167
Sierra Leone	Bo	235	0.671	-0.076	-0.234	0.081	-0.067	-0.206	0.072
Sierra Leone	Bombali	180	0.514	0.047	-0.123	0.216	0.046	-0.123	0.216
Sierra Leone	Bonthe	186	0.592	0.029	-0.140	0.199	0.028	-0.136	0.192
Sierra Leone	Falaba	141	0.575	-0.026	-0.220	0.168	-0.026	-0.215	0.164
Sierra Leone	Kailahun	162	0.572	0.182	0.003	0.361	0.178	0.003	0.353
Sierra Leone	Kambia	205	0.415	0.116	-0.045	0.276	0.112	-0.044	0.268
Sierra Leone	Karene	156	0.439	0.114	-0.068	0.297	0.113	-0.067	0.293
Sierra Leone	Kenema	252	0.658	0.100	-0.050	0.250	0.090	-0.045	0.225
Sierra Leone	Koinadugu	139	0.528	0.169	-0.023	0.360	0.168	-0.023	0.359
Sierra Leone	Kono	181	0.582	-0.031	-0.202	0.141	-0.030	-0.197	0.137
Sierra Leone	Moyamba	227	0.651	0.036	-0.122	0.194	0.033	-0.111	0.176
Sierra Leone	Port Loko	202	0.443	-0.106	-0.266	0.054	-0.105	-0.263	0.054
Sierra Leone	Pujehun	178	0.614	0.094	-0.081	0.268	0.089	-0.077	0.254
Sierra Leone	Tonkolili	190	0.489	0.020	-0.145	0.185	0.020	-0.145	0.185
Sierra Leone	Western Area Rural	203	0.468	0.086	-0.074	0.245	0.085	-0.074	0.244
Sierra Leone	Western Area Urban	193	0.473	0.103	-0.060	0.267	0.103	-0.060	0.266
South Africa	Eastern Cape	150	0.697	-0.186	-0.387	0.014	-0.157	-0.326	0.012
South Africa	Free State	97	0.723	0.120	-0.138	0.379	0.096	-0.111	0.303
South Africa	Gauteng	115	0.509	0.181	-0.029	0.391	0.181	-0.029	0.391
South Africa	Kwazulu Natal	185	0.592	-0.096	-0.265	0.074	-0.092	-0.256	0.071
South Africa	Limpopo	180	0.665	-0.165	-0.343	0.013	-0.147	-0.306	0.011
South Africa	Mpumalanga	162	0.663	0.000	-0.190	0.189	0.000	-0.169	0.169
South Africa	North West	135	0.479	-0.156	-0.351	0.038	-0.156	-0.350	0.038
South Africa	Northern Cape	88	0.699	-0.003	-0.269	0.262	-0.003	-0.227	0.221
South Africa	Western Cape	64	0.603	-0.093	-0.386	0.199	-0.089	-0.370	0.191
Tajikistan	Drs	584	0.673	0.002	-0.095	0.100	0.002	-0.084	0.088
Tajikistan	Dushanbe	277	0.695	-0.072	-0.127	-0.017	-0.061	-0.108	-0.014
Tajikistan	Gbao	179	0.642	0.136	-0.027	0.299	0.125	-0.025	0.275
Tajikistan	Khatlon	722	0.867	-0.076	-0.197	0.044	-0.035	-0.091	0.020
Tajikistan	Sughd	428	0.924	-0.122	-0.323	0.079	-0.034	-0.091	0.022
Tanzania	Arusha	104	0.832	0.172	-0.126	0.470	0.096	-0.070	0.263
Tanzania	Dar Es Salaam	152	0.814	-0.107	-0.344	0.130	-0.064	-0.208	0.079
Tanzania	Dodoma	82	0.823	-0.134	-0.464	0.196	-0.078	-0.270	0.114
Tanzania	Geita	171	0.624	0.013	-0.167	0.193	0.012	-0.157	0.181
Tanzania	Iringa	73	0.820	0.159	-0.189	0.507	0.094	-0.111	0.299
Tanzania	Kagera	122	0.895	0.186	-0.150	0.522	0.070	-0.056	0.195
Tanzania	Kaskazini Pemba	113	0.817	0.208	-0.067	0.483	0.124	-0.040	0.289
Tanzania	Kaskazini Unguja	99	0.938	0.106	-0.371	0.582	0.025	-0.086	0.135
Tanzania	Katavi	164	0.606	0.321	0.146	0.496	0.306	0.139	0.474
Tanzania	Kigoma	147	0.756	0.217	0.001	0.433	0.160	0.001	0.319
Tanzania	Kilimanjaro	50	0.909	-0.107	-0.674	0.460	-0.036	-0.223	0.152
Tanzania	Kusini Pemba	103	0.811	0.110	-0.177	0.397	0.067	-0.108	0.243
Tanzania	Kusini Unguja	91	0.857	0.214	-0.126	0.553	0.105	-0.062	0.272
Tanzania	Lindi	79	0.744	0.337	0.051	0.623	0.257	0.039	0.474
Tanzania	Manyara	118	0.898	-0.105	-0.453	0.242	-0.038	-0.165	0.088
Tanzania	Mara	189	0.700	0.101	-0.079	0.281	0.085	-0.066	0.236
Tanzania	Mbeya	73	0.673	0.186	-0.097	0.469	0.164	-0.086	0.413
Tanzania	Mjini Magharibi	112	0.788	-0.168	-0.430	0.094	-0.112	-0.287	0.063
Tanzania	Morogoro	94	0.797	0.237	-0.052	0.526	0.153	-0.034	0.340
Tanzania	Mtwara	70	0.858	0.049	-0.344	0.442	0.024	-0.168	0.216
Tanzania	Mwanza	130	0.596	0.252	0.053	0.451	0.243	0.051	0.435
Tanzania	Njombe	70	0.831	-0.197	-0.560	0.166	-0.111	-0.315	0.093
Tanzania	Pwani	76	0.814	0.166	-0.170	0.502	0.100	-0.103	0.304
Tanzania	Rukwa	139	0.760	0.030	-0.197	0.256	0.022	-0.143	0.187
Tanzania	Ruvuma	83	0.787	0.245	-0.058	0.547	0.164	-0.039	0.367
Tanzania	Shinyanga	147	0.673	-0.099	-0.298	0.101	-0.087	-0.263	0.089
Tanzania	Simiyu	209	0.735	0.089	-0.089	0.267	0.069	-0.069	0.208
Tanzania	Singida	125	0.767	0.273	0.036	0.509	0.195	0.026	0.364

Tanzania	Tabora	194	0.532	0.293	0.134	0.451	0.291	0.134	0.449
Tanzania	Tanga	106	0.774	0.109	-0.155	0.374	0.076	-0.109	0.262
The Gambia	Banjul	137	0.783	0.193	-0.040	0.427	0.132	-0.028	0.291
The Gambia	Basse	524	0.853	-0.051	-0.191	0.088	-0.026	-0.096	0.044
The Gambia	Brikama	495	0.822	0.021	-0.113	0.154	0.012	-0.066	0.090
The Gambia	Janjanbureh	300	0.826	0.071	-0.102	0.243	0.041	-0.059	0.140
The Gambia	Kanifing	239	0.768	0.250	0.078	0.421	0.178	0.056	0.300
The Gambia	Kerewan	348	0.906	-0.267	-0.473	-0.061	-0.091	-0.162	-0.021
The Gambia	Kuntaur	358	0.840	0.007	-0.157	0.170	0.004	-0.084	0.092
The Gambia	Mansakonko	248	0.907	-0.176	-0.423	0.071	-0.059	-0.143	0.024
Timor-Leste	Aileu	169	0.568	-0.059	-0.235	0.118	-0.058	-0.231	0.116
Timor-Leste	Ainaro	185	0.287	0.216	0.034	0.398	0.177	0.027	0.326
Timor-Leste	Baucau	177	0.634	0.128	-0.049	0.304	0.119	-0.045	0.282
Timor-Leste	Bobonaro	197	0.469	0.105	-0.057	0.266	0.104	-0.057	0.265
Timor-Leste	Cova Lima	154	0.401	0.425	0.250	0.599	0.408	0.240	0.576
Timor-Leste	Dili	277	0.482	0.039	-0.097	0.176	0.039	-0.097	0.176
Timor-Leste	Ermera	199	0.275	0.145	-0.034	0.325	0.116	-0.027	0.259
Timor-Leste	Lautem	161	0.565	0.118	-0.062	0.298	0.116	-0.061	0.293
Timor-Leste	Liquica	191	0.488	0.044	-0.120	0.209	0.044	-0.120	0.209
Timor-Leste	Manatuto	183	0.555	0.231	0.065	0.397	0.228	0.064	0.392
Timor-Leste	Manufahi	198	0.274	0.290	0.114	0.467	0.231	0.091	0.372
Timor-Leste	Oecussi	146	0.396	0.127	-0.064	0.319	0.122	-0.062	0.305
Timor-Leste	Viqueque	179	0.443	0.148	-0.022	0.318	0.146	-0.021	0.314
Uganda	Acholi	309	0.620	0.021	-0.112	0.154	0.020	-0.106	0.145
Uganda	Ankole	304	0.649	0.082	-0.054	0.218	0.075	-0.049	0.199
Uganda	Bugishu	254	0.441	0.054	-0.089	0.198	0.053	-0.088	0.195
Uganda	Bukedi	386	0.512	0.141	0.027	0.256	0.141	0.027	0.256
Uganda	Bunyoro	340	0.625	0.084	-0.043	0.211	0.079	-0.040	0.198
Uganda	Busoga	445	0.509	0.053	-0.055	0.160	0.053	-0.055	0.160
Uganda	Central 1	413	0.440	-0.114	-0.226	-0.002	-0.113	-0.223	-0.002
Uganda	Central 2	403	0.467	0.200	0.089	0.312	0.199	0.088	0.310
Uganda	Kampala	264	0.451	0.067	-0.073	0.207	0.067	-0.072	0.205
Uganda	Karamoja	285	0.664	-0.013	-0.155	0.129	-0.012	-0.139	0.115
Uganda	Kigezi	228	0.700	0.051	-0.114	0.215	0.042	-0.095	0.180
Uganda	Lango	327	0.465	0.138	0.013	0.263	0.137	0.013	0.261
Uganda	Teso	387	0.642	0.145	0.026	0.265	0.134	0.024	0.243
Uganda	Tooro	400	0.535	-0.020	-0.133	0.094	-0.020	-0.133	0.094
Uganda	West Nile	382	0.636	0.090	-0.030	0.210	0.083	-0.028	0.195
Zambia	Central	344	0.749	0.240	0.101	0.379	0.180	0.076	0.285
Zambia	Copperbelt	300	0.802	-0.031	-0.196	0.133	-0.020	-0.124	0.085
Zambia	Eastern	395	0.790	-0.031	-0.171	0.109	-0.021	-0.114	0.072
Zambia	Luapula	396	0.645	0.186	0.068	0.304	0.170	0.063	0.278
Zambia	Lusaka	346	0.743	-0.034	-0.173	0.106	-0.026	-0.132	0.081
Zambia	Muchinga	303	0.657	0.260	0.126	0.395	0.235	0.114	0.356
Zambia	North Western	278	0.742	-0.126	-0.281	0.028	-0.097	-0.216	0.022
Zambia	Northern	316	0.782	0.255	0.103	0.407	0.174	0.070	0.278
Zambia	Southern	357	0.706	-0.096	-0.228	0.035	-0.080	-0.189	0.029
Zambia	Western	288	0.638	0.066	-0.073	0.205	0.061	-0.067	0.190
Zimbabwe	Bulawayo	146	0.865	0.087	-0.189	0.363	0.040	-0.088	0.169
Zimbabwe	Harare Chitungwiza	243	0.785	0.139	-0.038	0.316	0.094	-0.025	0.213
Zimbabwe	Manicaland	211	0.695	0.166	-0.003	0.334	0.141	-0.002	0.284
Zimbabwe	Mashonaland Central	224	0.773	0.290	0.113	0.467	0.204	0.079	0.328
Zimbabwe	Mashonaland East	181	0.859	0.210	-0.031	0.451	0.102	-0.015	0.218
Zimbabwe	Mashonaland West	227	0.689	0.087	-0.075	0.250	0.075	-0.064	0.214
Zimbabwe	Masvingo	228	0.744	0.045	-0.127	0.218	0.035	-0.097	0.166
Zimbabwe	Matabeleland North	173	0.936	-0.168	-0.520	0.184	-0.040	-0.125	0.044
Zimbabwe	Matabeleland South	146	0.800	0.196	-0.037	0.430	0.126	-0.024	0.275
Zimbabwe	Midlands	230	0.705	0.025	-0.139	0.189	0.021	-0.116	0.157
