# Supplementary material for: Essential childhood immunization in 43 low- and middle-income countries: Analysis of spatial trends and socioeconomic inequalities in vaccine coverage
Source: PLoS Med. 2023 Jan 17;20(1):e1004166. doi: 10.1371/journal.pmed.1004166 (PMC9888726; doi:10.1371/journal.pmed.1004166)
Supplement: S6 Table — (PDF) [file pmed.1004166.s006.pdf]

Table S6. Theil index of inequality by country for individual vaccinations, full immunization coverage (FIC), and Wagstaff's (W) and Erreygers' (E) indices of inequality.

Country	BCG	DTP	OPV	MCV	FIC	W	E
Afghanistan	0.066	0.099	0.052	0.079	0.127	0.210	0.245
Albania	0.001	0.001	0.001	0.001	0.001	0.203	0.248
Angola	0.027	0.069	0.078	0.040	0.106	0.103	0.113
Armenia	0.000	0.002	0.002	0.002	0.003	0.151	0.302
Bangladesh	0.000	0.000	0.000	0.000	0.000	0.133	0.065
Benin	0.005	0.015	0.016	0.017	0.028	0.326	0.342
Burundi	0.000	0.000	0.002	0.000	0.004	0.362	0.378
Cambodia	0.001	0.004	0.004	0.004	0.007	0.230	0.337
Cameroon	0.004	0.014	0.009	0.013	0.024	0.187	0.195
Chad	0.075	0.168	0.090	0.059	0.232	0.288	0.236
Egypt	0.000	0.000	0.000	0.020	0.024	0.342	0.347
Ethiopia	0.050	0.084	0.071	0.054	0.120	0.234	0.302
Ghana	0.000	0.002	0.003	0.001	0.005	0.154	0.174
Guatemala	0.000	0.001	0.001	0.002	0.003	0.205	0.229
Guinea	0.008	0.056	0.035	0.055	0.110	0.153	0.090
Haiti	0.003	0.013	0.010	0.010	0.022	0.119	0.110
India	0.001	0.004	0.008	0.022	0.037	0.329	0.309
Indonesia	0.003	0.011	0.013	0.006	0.017	0.243	0.274
Jordan	0.001	0.002	0.003	0.002	0.003	0.384	0.445
Kenya	0.002	0.006	0.012	0.005	0.022	0.286	0.274
Lesotho	0.000	0.003	0.005	0.000	0.006	0.370	0.375
Liberia	0.001	0.007	0.016	0.004	0.023	0.183	0.180
Madagascar	0.017	0.030	0.035	0.028	0.047	0.255	0.267
Malawi	0.000	0.001	0.003	0.001	0.004	0.217	0.203
Maldives	0.002	0.001	0.002	0.002	0.001	0.109	0.104
Mali	0.070	0.124	0.112	0.082	0.023	0.506	0.410
Mauritania	0.002	0.010	0.024	0.006	0.030	0.271	0.286
Myanmar	0.002	0.016	0.009	0.004	0.024	0.177	0.212
Nepal	0.000	0.003	0.001	0.001	0.004	0.321	0.189
Nigeria	0.056	0.111	0.053	0.055	0.135	0.131	0.159
Pakistan	0.025	0.034	0.006	0.047	0.048	0.164	0.229
Philippines	0.009	0.024	0.019	0.018	0.031	0.144	0.142
Rwanda	0.000	0.000	0.000	0.000	0.000	0.106	0.055
Senegal	0.001	0.001	0.001	0.002	0.003	0.329	0.375
Sierra Leone	0.000	0.003	0.004	0.003	0.011	0.319	0.322
South Africa	0.001	0.005	0.007	0.001	0.009	0.246	0.263
Tajikistan	0.001	0.005	0.004	0.003	0.011	0.232	0.336
Tanzania	0.001	0.004	0.005	0.003	0.008	0.228	0.318
The Gambia	0.000	0.001	0.001	0.001	0.002	0.324	0.389

Timor-Leste	0.007	0.015	0.028	0.008	0.033	0.203	0.197
Uganda	0.000	0.002	0.010	0.002	0.013	0.269	0.277
Zambia	0.000	0.001	0.002	0.001	0.003	0.220	0.242
Zimbabwe	0.002	0.004	0.004	0.003	0.005	0.204	0.248
