# Supplementary material for: Essential childhood immunization in 43 low- and middle-income countries: Analysis of spatial trends and socioeconomic inequalities in vaccine coverage
Source: PLoS Med. 2023 Jan 17;20(1):e1004166. doi: 10.1371/journal.pmed.1004166 (PMC9888726; doi:10.1371/journal.pmed.1004166)

Fig S1. Location of principle sampling units (PSUs) for which geocoordinates (latitude and longitude) are available in DHS. No geocoordinate information is available for PSUs in Afghanistan, Maldives, Mauritania, and Indonesia. Spatial boundaries were retrieved from Natural Earth (https://www.naturalearthdata.com/) using "rnaturalearth" package (https://github.com/ropenscilabs/rnaturalearth).

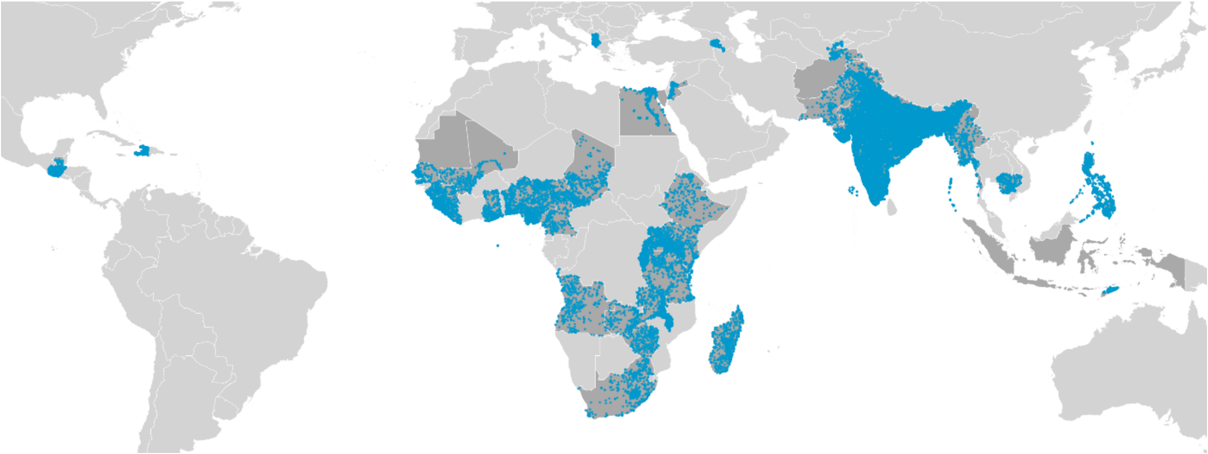

Supplement: S1 Fig — No geocoordinate information is available for PSUs in Afghanistan, Maldives, Mauritania, and Indonesia. Spatial boundaries were retrieved from Natural Earth (https://www.naturalearthdata.com/) using the “rnaturalearth” package (https://github.com/ropenscilabs/rnaturalearth). (PDF) [file pmed.1004166.s007.pdf]
