# Supplementary material for: Essential childhood immunization in 43 low- and middle-income countries: Analysis of spatial trends and socioeconomic inequalities in vaccine coverage
Source: PLoS Med. 2023 Jan 17;20(1):e1004166. doi: 10.1371/journal.pmed.1004166 (PMC9888726; doi:10.1371/journal.pmed.1004166)

Fig S2. A concentration curve based on DHS survey data from Nigeria for children aged 15 to 35 months. The *y*-axis shows the cumulative share of children who are fully immunized, and the *x*-axis shows the cumulative share of children ranked by wealth index from the poorest to the richest. The green 45° line represents a state of perfect equality.

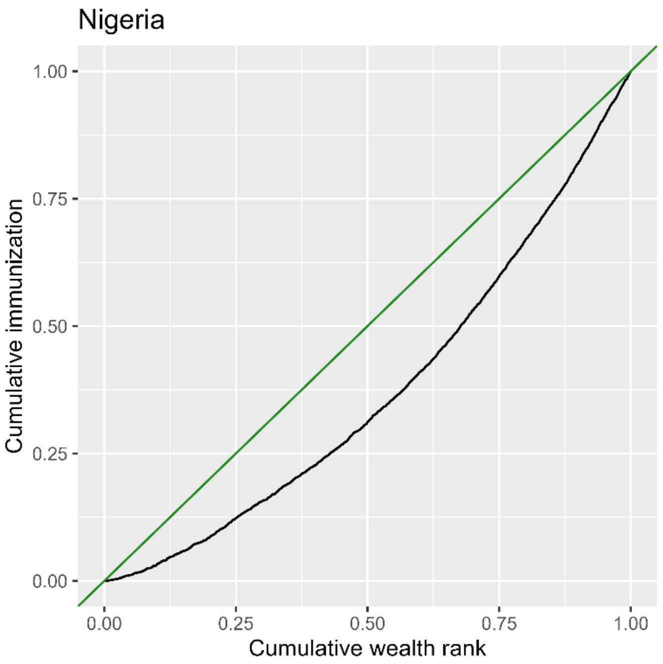

Supplement: S2 Fig — The y-axis shows the cumulative share of children who are fully immunized, and the x-axis shows the cumulative share of children ranked by wealth index from the poorest to the richest. The green 45° line represents a state of perfect equality. (PDF) [file pmed.1004166.s008.pdf]
