# Supplementary material for: Essential childhood immunization in 43 low- and middle-income countries: Analysis of spatial trends and socioeconomic inequalities in vaccine coverage
Source: PLoS Med. 2023 Jan 17;20(1):e1004166. doi: 10.1371/journal.pmed.1004166 (PMC9888726; doi:10.1371/journal.pmed.1004166)

Fig S4. Bivariate map showing the intersection between full immunization coverage (FIC) and Wagstaff's (W) index of inequality. Spatial boundaries were retrieved from Natural Earth (https://www.naturalearthdata.com/) using "rnaturalearth" package (https://github.com/ropenscilabs/rnaturalearth).

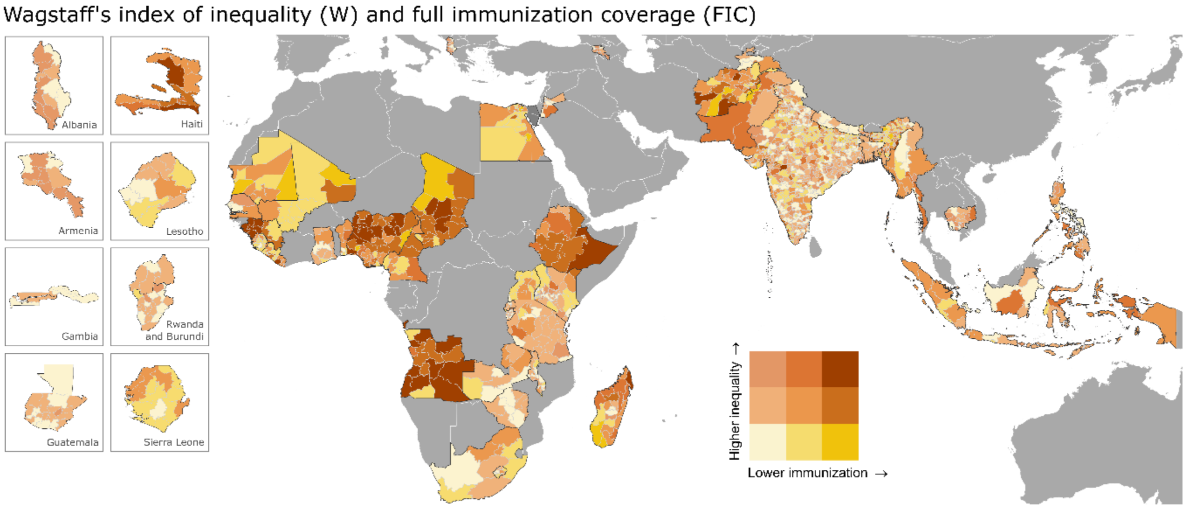

Supplement: S4 Fig — Spatial boundaries were retrieved from Natural Earth (https://www.naturalearthdata.com/) using the “rnaturalearth” package (https://github.com/ropenscilabs/rnaturalearth). (PDF) [file pmed.1004166.s010.pdf]
