# Supplementary material for: Essential childhood immunization in 43 low- and middle-income countries: Analysis of spatial trends and socioeconomic inequalities in vaccine coverage
Source: PLoS Med. 2023 Jan 17;20(1):e1004166. doi: 10.1371/journal.pmed.1004166 (PMC9888726; doi:10.1371/journal.pmed.1004166)
Supplement: S6 Fig — The black dots represent vaccine combinations and the bars represent the number of missed vaccinations for each vaccine combination. Note that multiple doses of DTP and OPV are needed to reach full immunization; therefore, the presented estimates do not refer to the number of missed doses but complete vaccinations. If a child is missing 2 or more doses of a specific vaccine, the child will be counted only once. (PDF) [file pmed.1004166.s012.pdf]

Fig S6. Intersecting sets of missed vaccinations for children aged 15 to 35 months for 43 countries. The black dots represent vaccine combinations and the bars represent the number of missed vaccinations for each vaccine combination. Note that multiple doses of DTP and OPV are needed to reach full immunization, therefore, the presented estimates do not refer to the number of missed doses but complete vaccinations. If a child is missing two or more doses of a specific vaccine, the child will be counted only once.

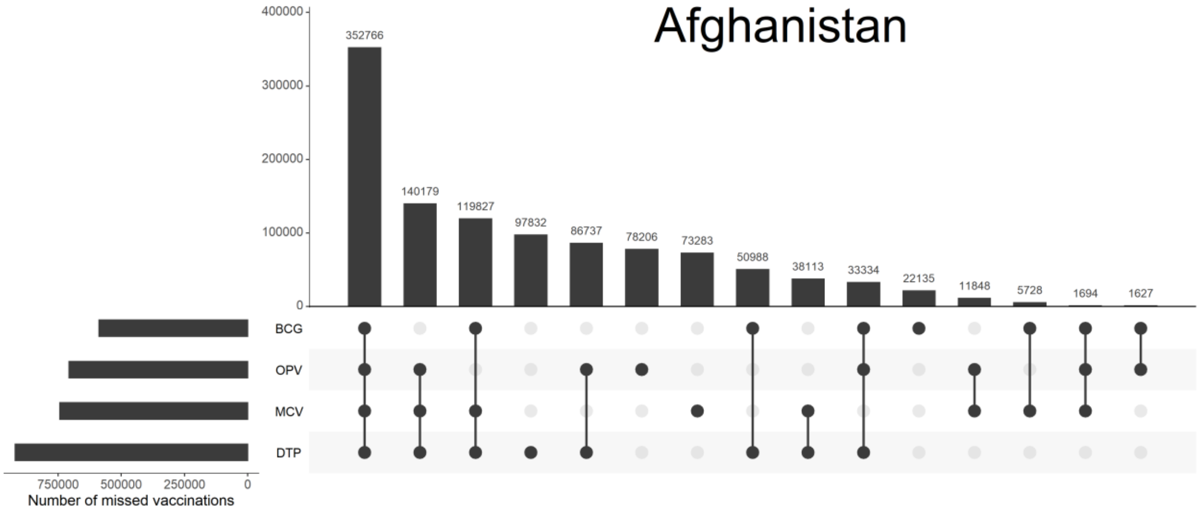

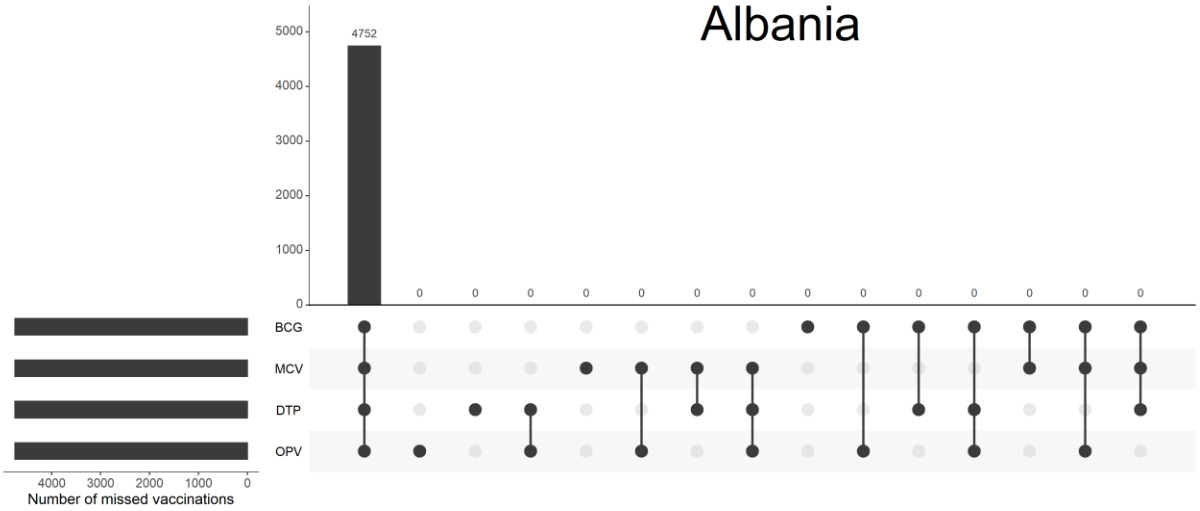

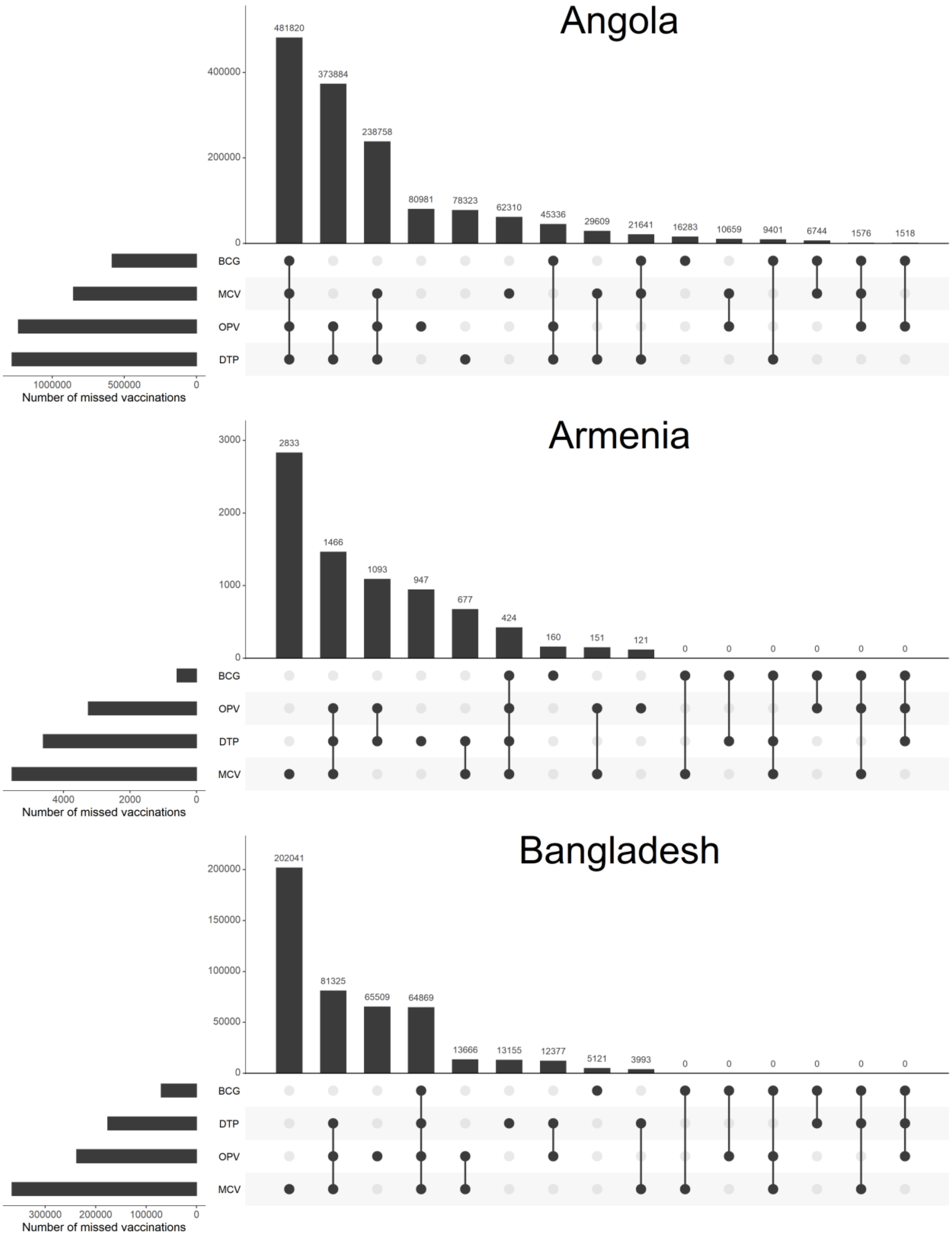

Angola
481820
373884
238758
80981
78323
62310
45336
29609
21641
16283
10659
9401
6744
1576
1518
400000
200000
0
BCG
MCV
OPV
DTP
1000000
500000
0
Number of missed vaccinations
Armenia
2833
1466
1093
947
677
424
160
151
121
0
0
0
0
0
0
3000
2000
1000
0
BCG
OPV
DTP
MCV
4000
2000
0
Number of missed vaccinations
Bangladesh
202041
81325
65509
64869
13666
13155
12377
5121
3993
0
0
0
0
0
0
200000
150000
100000
50000
0
BCG
DTP
OPV
MCV
300000
200000
100000
0
Number of missed vaccinations

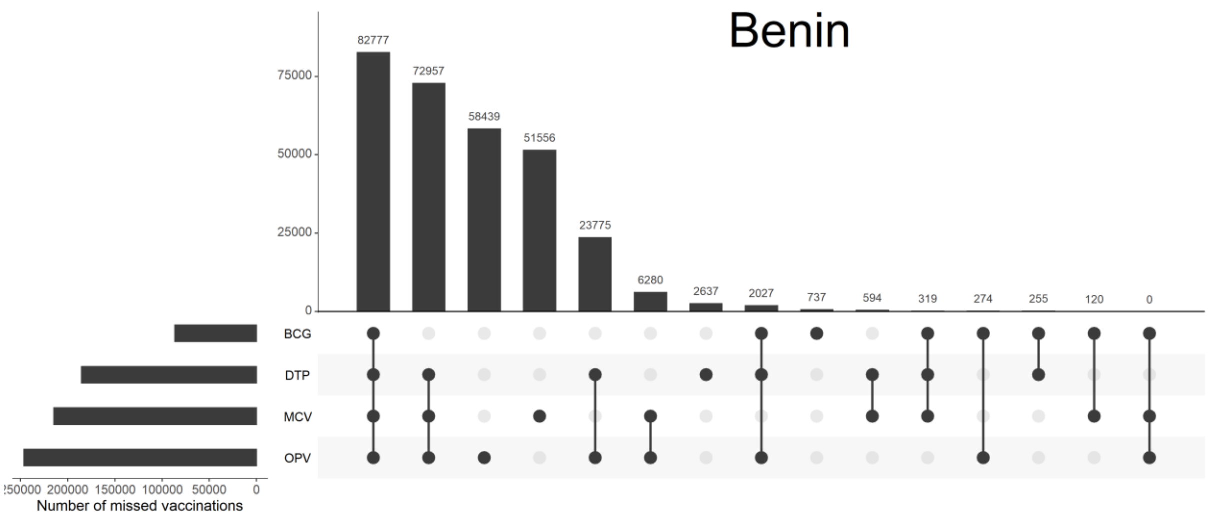
Benin
82777
72957
58439
51556
23775
6280
2637
2027
737
594
319
274
255
120
0
75000
50000
25000
0
BCG
DTP
MCV
OPV
200000 150000 100000 50000 0
Number of missed vaccinations

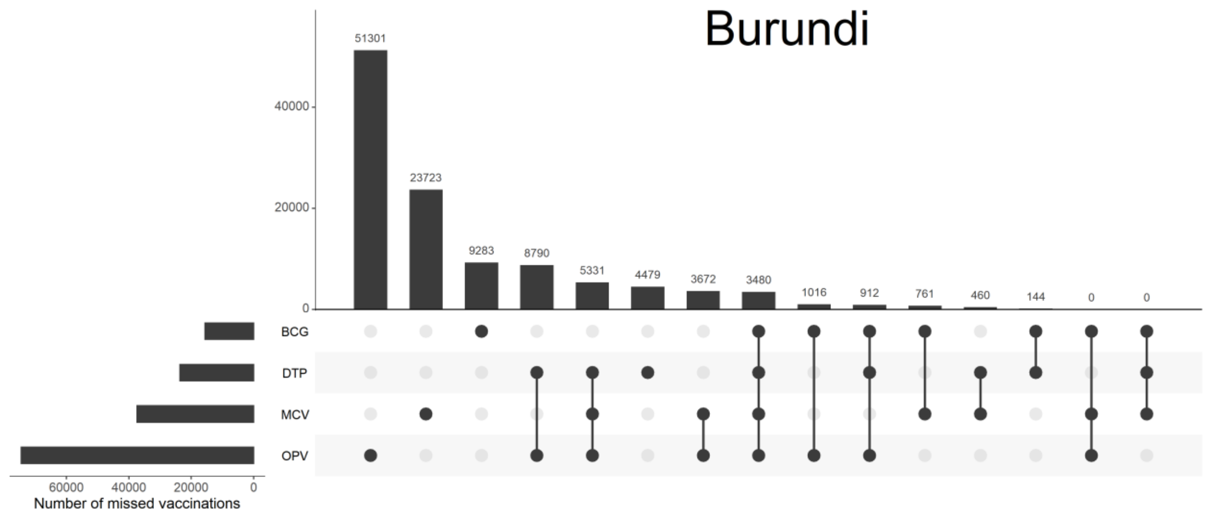
Burundi
51301
23723
9283
8790
5331
4479
3672
3480
1016
912
761
460
144
0
0
40000
20000
0
BCG
DTP
MCV
OPV
60000 40000 20000 0
Number of missed vaccinations

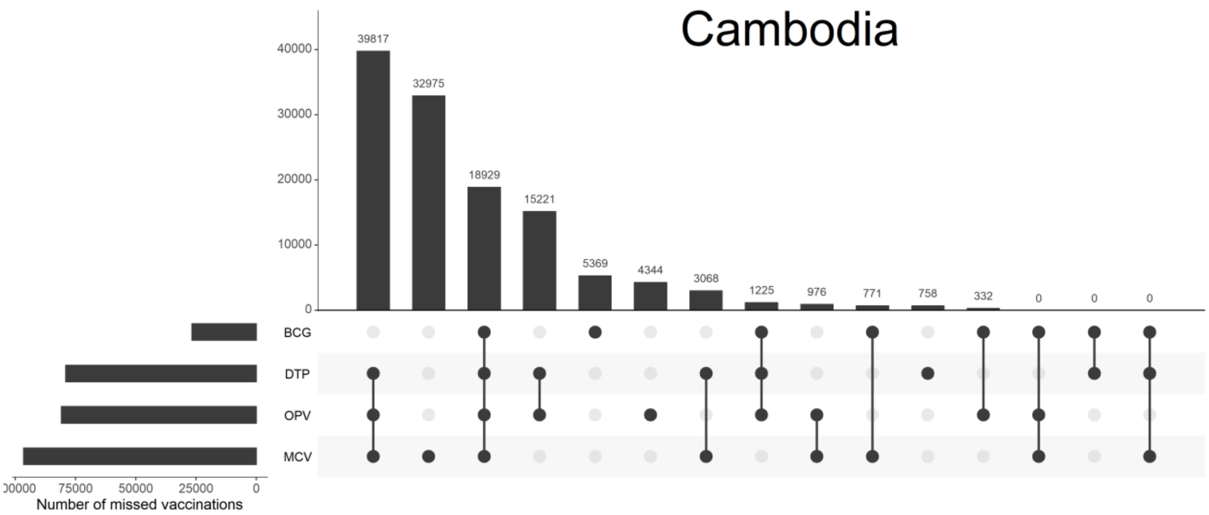
Cambodia
39817
32975
18929
15221
5369
4344
3068
1225
976
771
758
332
0
0
0
40000
30000
20000
10000
0
BCG
DTP
OPV
MCV
75000 50000 25000 0
Number of missed vaccinations

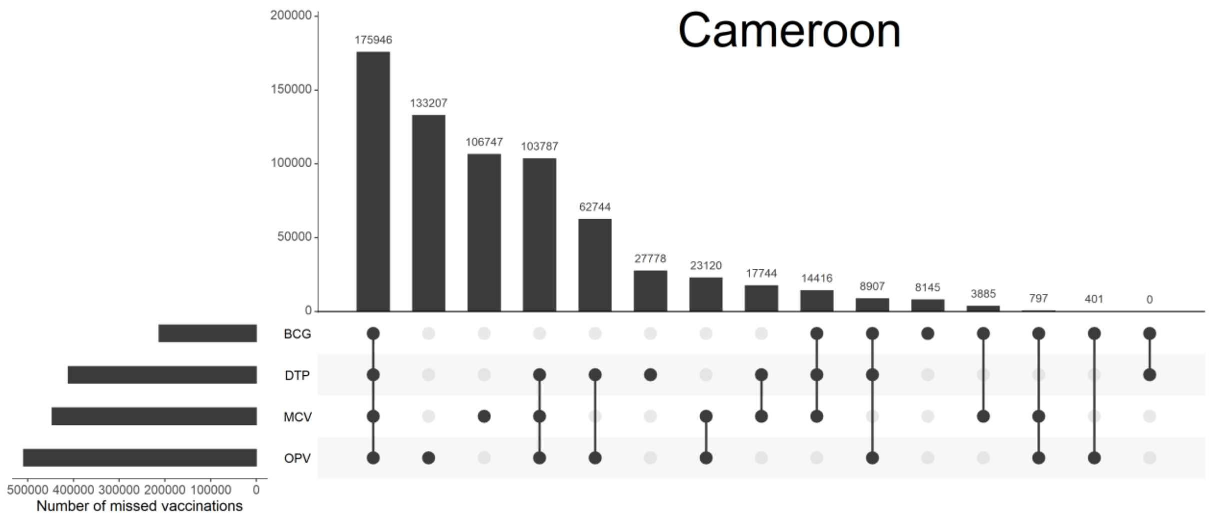
Cameroon
200000
150000
100000
50000
0
175946
133207
106747
103787
62744
27778
23120
17744
14416
8907
8145
3885
797
401
0
BCG
DTP
MCV
OPV
500000 400000 300000 200000 100000 0
Number of missed vaccinations

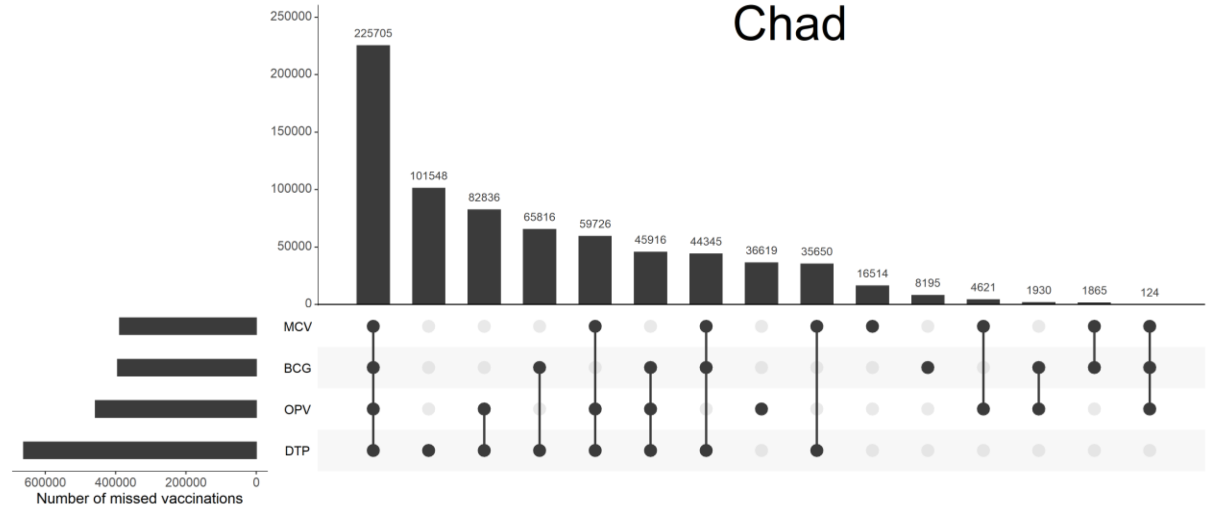
Chad
250000
200000
150000
100000
50000
0
225705
101548
82836
65816
59726
45916
44345
36619
35650
16514
8195
4621
1930
1865
124
MCV
BCG
OPV
DTP
600000 400000 200000 0
Number of missed vaccinations

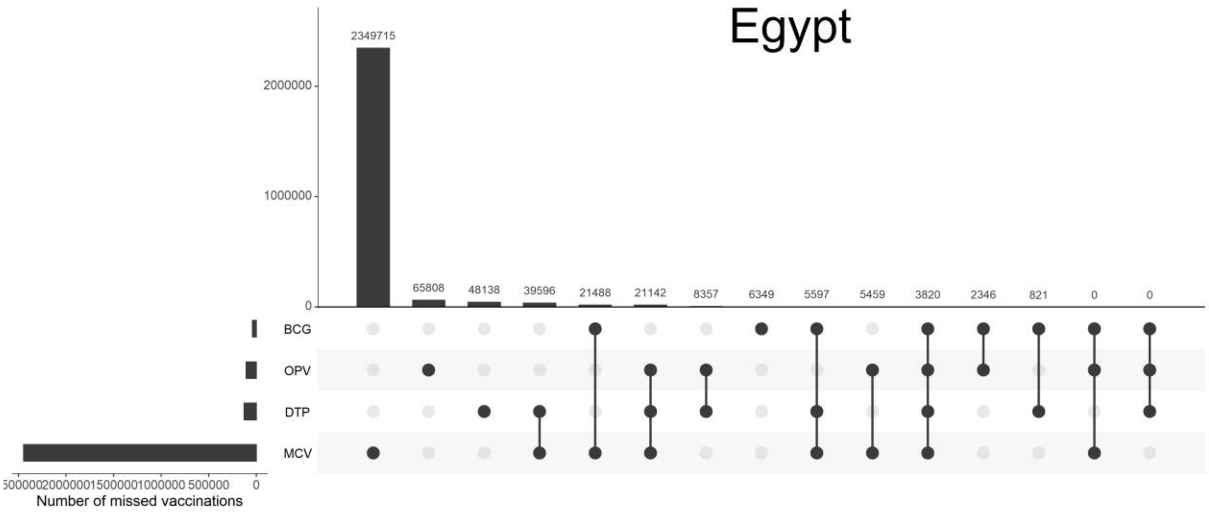
Egypt
2000000
1000000
0
2349715
65808
48138
39596
21488
21142
8357
6349
5597
5459
3820
2346
821
0
0
BCG
OPV
DTP
MCV
Number of missed vaccinations

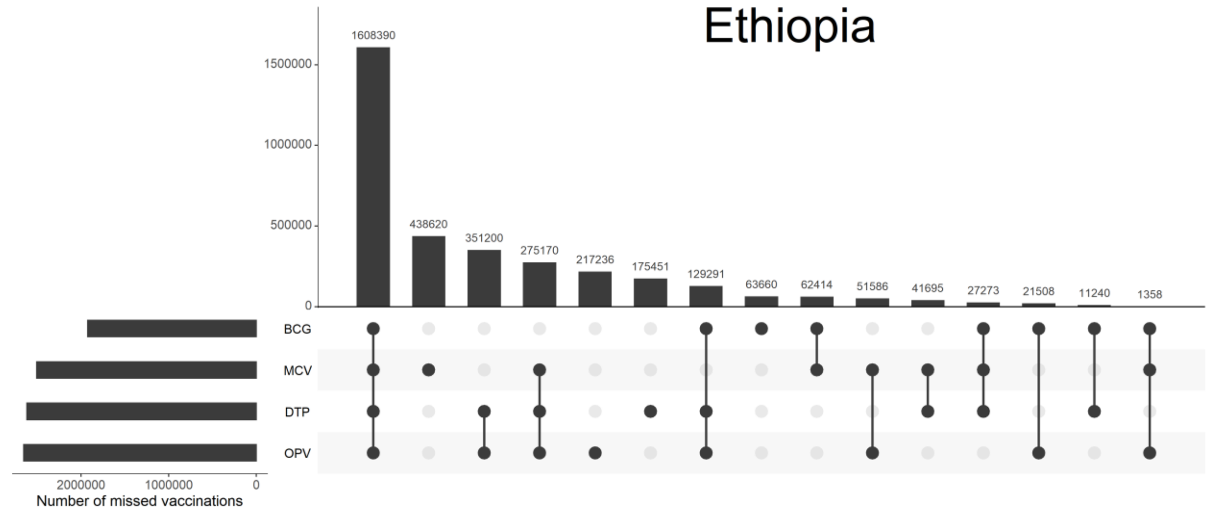
Ethiopia
1608390
438620
351200
275170
217236
175451
129291
63660
62414
51586
41695
27273
21508
11240
1358
1500000
1000000
500000
0
BCG
MCV
DTP
OPV
2000000
1000000
0
Number of missed vaccinations

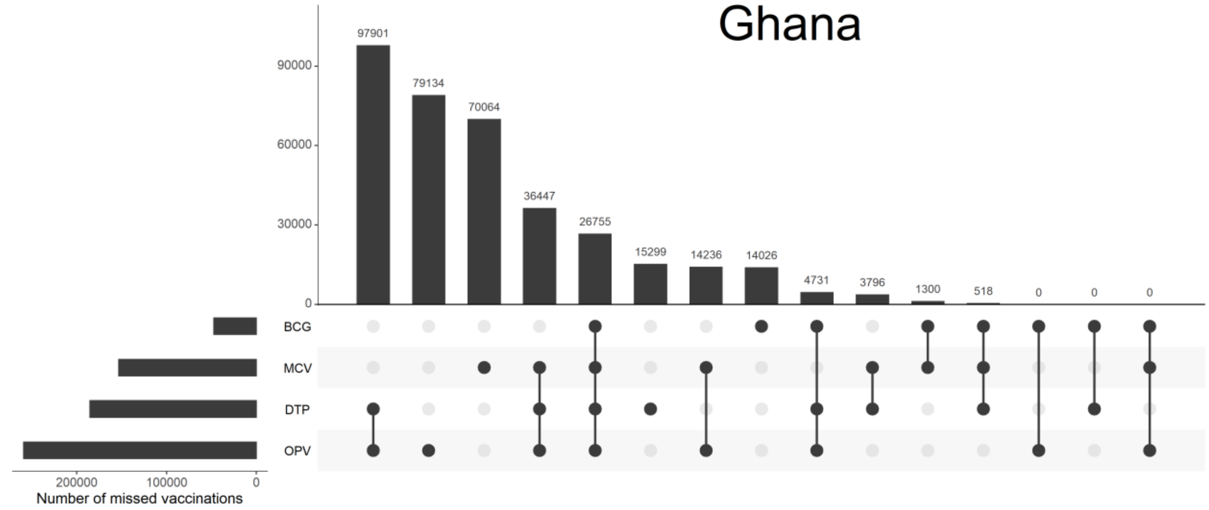
Ghana
97901
79134
70064
36447
26755
15299
14236
14026
4731
3796
1300
518
0
0
0
90000
60000
30000
0
BCG
MCV
DTP
OPV
200000
100000
0
Number of missed vaccinations

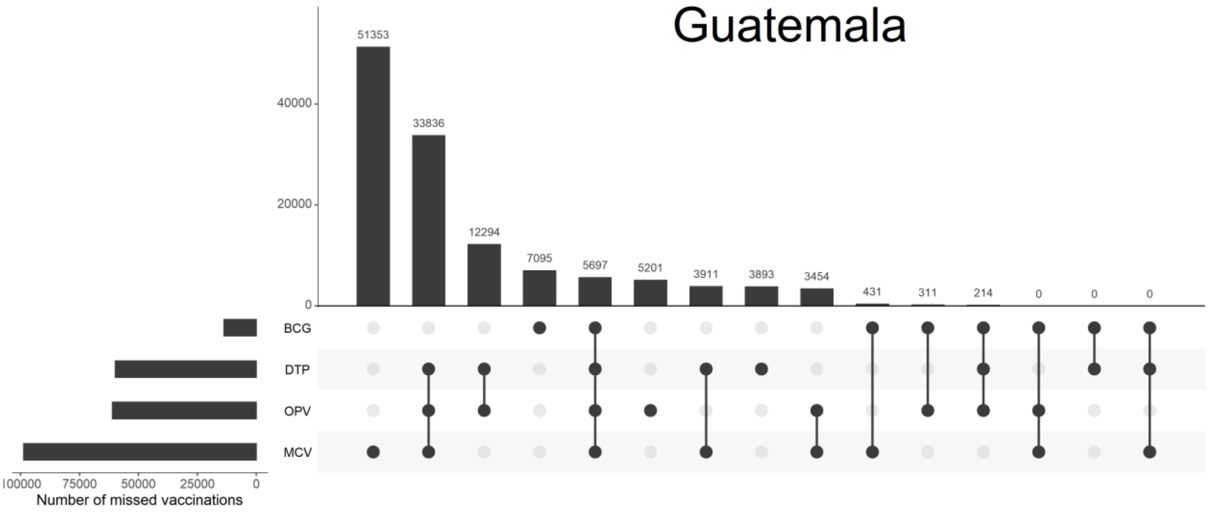
Guatemala
51353
33836
12294
7095
5697
5201
3911
3893
3454
431
311
214
0
0
0
40000
20000
0
BCG
DTP
OPV
MCV
100000
75000
50000
25000
0
Number of missed vaccinations

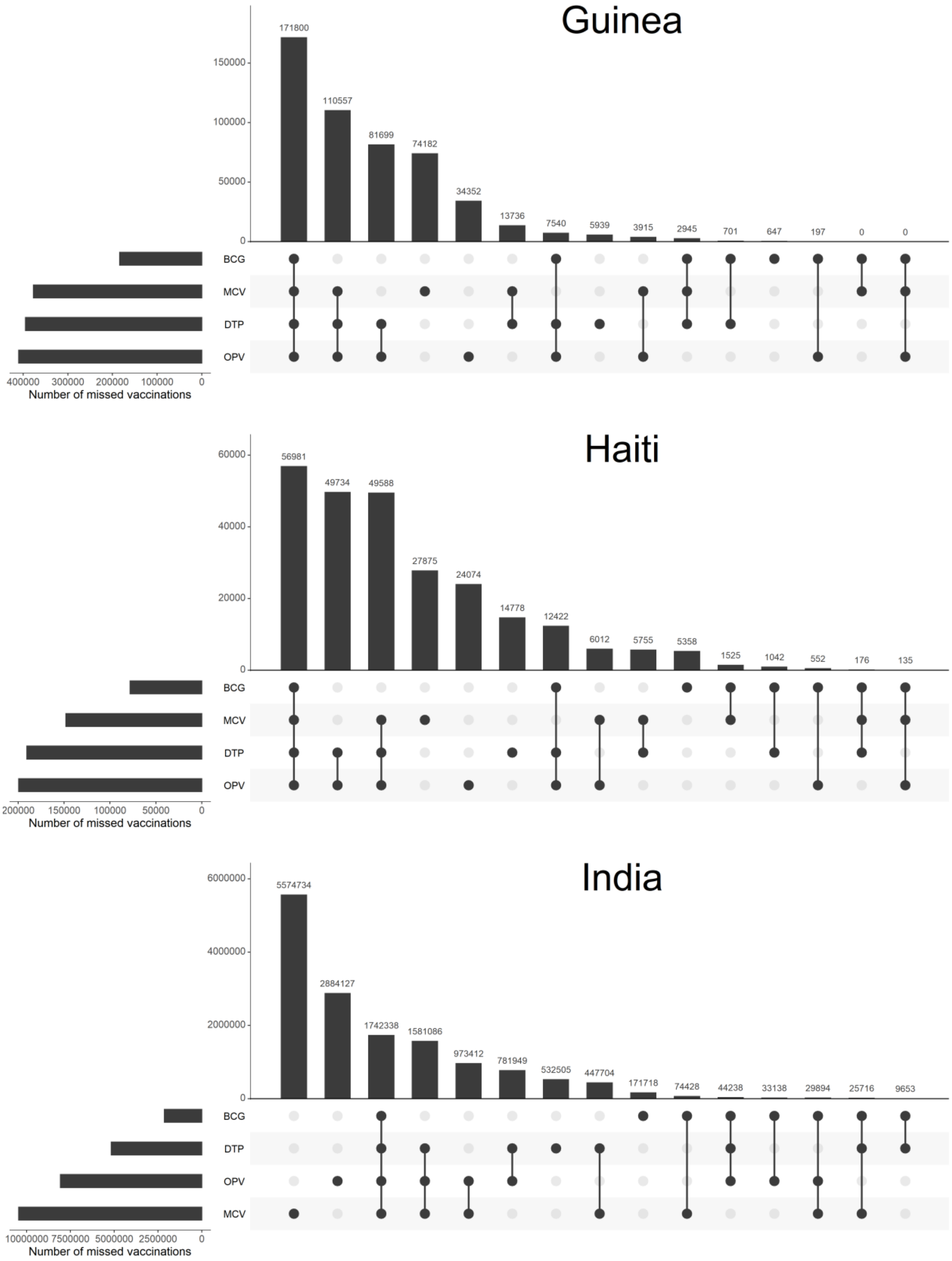
Guinea
171800
110557
81699
74182
34352
13736
7540
5939
3915
2945
701
647
197
0
0
150000
100000
50000
0
BCG
MCV
DTP
OPV
400000 300000 200000 100000 0
Number of missed vaccinations
Haiti
56981
49734
49588
27875
24074
14778
12422
6012
5755
5358
1525
1042
552
176
135
60000
40000
20000
0
BCG
MCV
DTP
OPV
200000 150000 100000 50000 0
Number of missed vaccinations
India
5574734
2884127
1742338
1581086
973412
781949
532505
447704
171718
74428
44238
33138
29894
25716
9653
6000000
4000000
2000000
0
BCG
DTP
OPV
MCV
10000000 7500000 5000000 2500000 0
Number of missed vaccinations

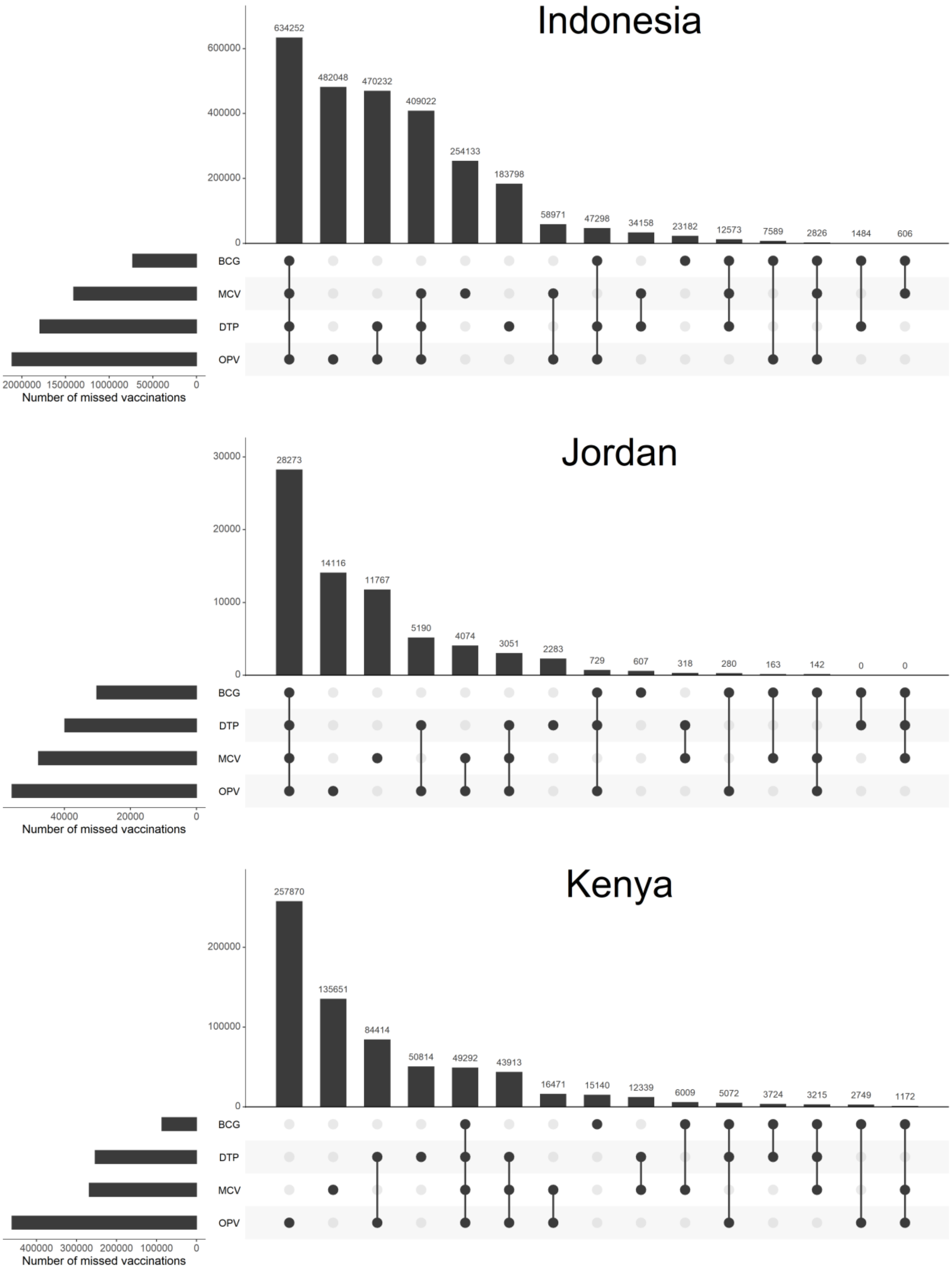

Indonesia
600000
400000
200000
0
634252
482048
470232
409022
254133
183798
58971
47298
34158
23182
12573
7589
2826
1484
606
BCG
MCV
DTP
OPV
2000000 1500000 1000000 500000 0
Number of missed vaccinations
Jordan
30000
20000
10000
0
28273
14116
11767
5190
4074
3051
2283
729
607
318
280
163
142
0
0
BCG
DTP
MCV
OPV
40000 20000 0
Number of missed vaccinations
Kenya
200000
100000
0
257870
135651
84414
50814
49292
43913
16471
15140
12339
6009
5072
3724
3215
2749
1172
BCG
DTP
MCV
OPV
400000 300000 200000 100000 0
Number of missed vaccinations

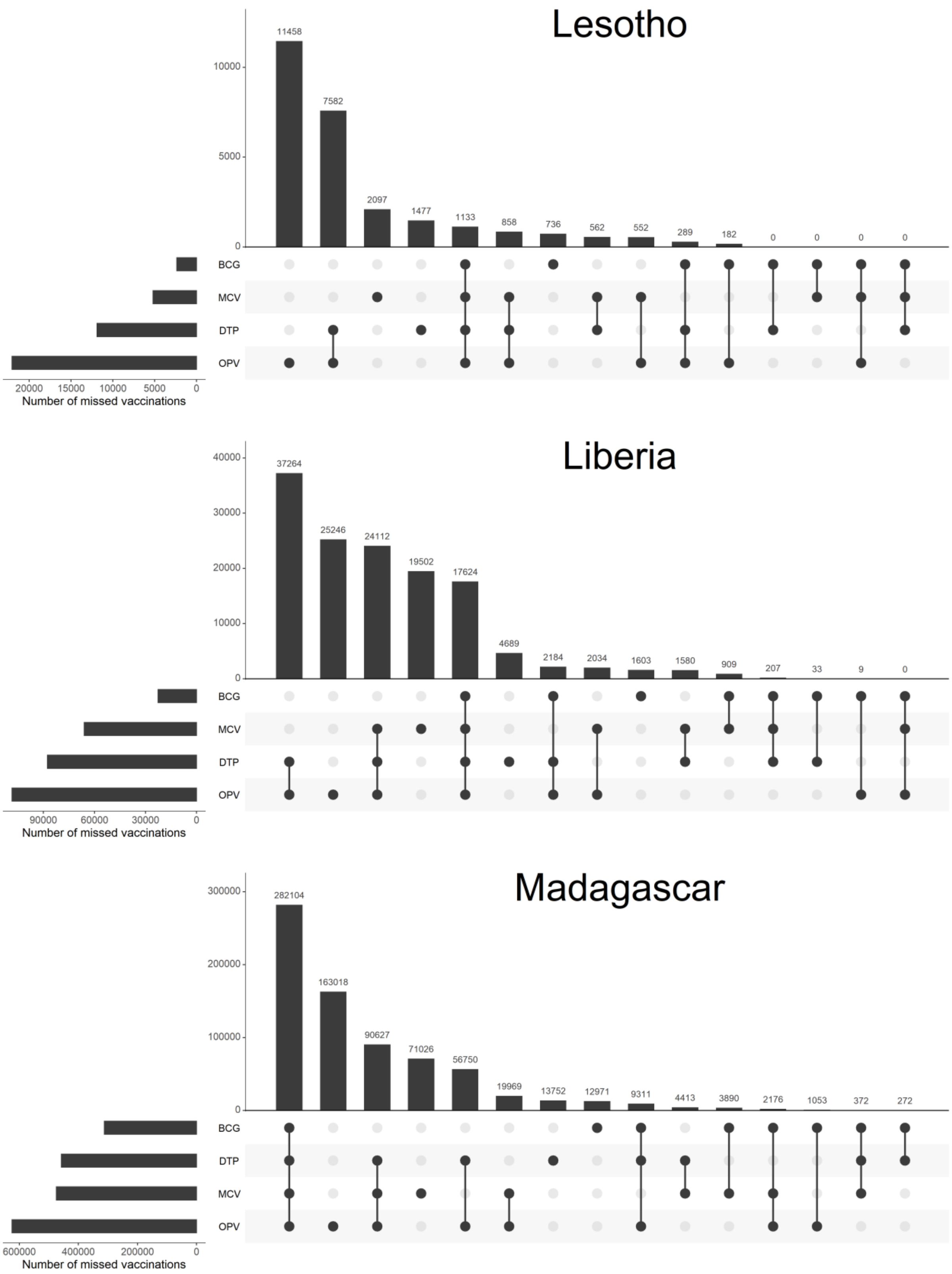

Lesotho
11458
7582
2097
1477
1133
858
736
562
552
289
182
0
0
0
0
BCG
MCV
DTP
OPV
Number of missed vaccinations
Liberia
37264
25246
24112
19502
17624
4689
2184
2034
1603
1580
909
207
33
9
0
BCG
MCV
DTP
OPV
Number of missed vaccinations
Madagascar
282104
163018
90627
71026
56750
19969
13752
12971
9311
4413
3890
2176
1053
372
272
BCG
DTP
MCV
OPV
Number of missed vaccinations

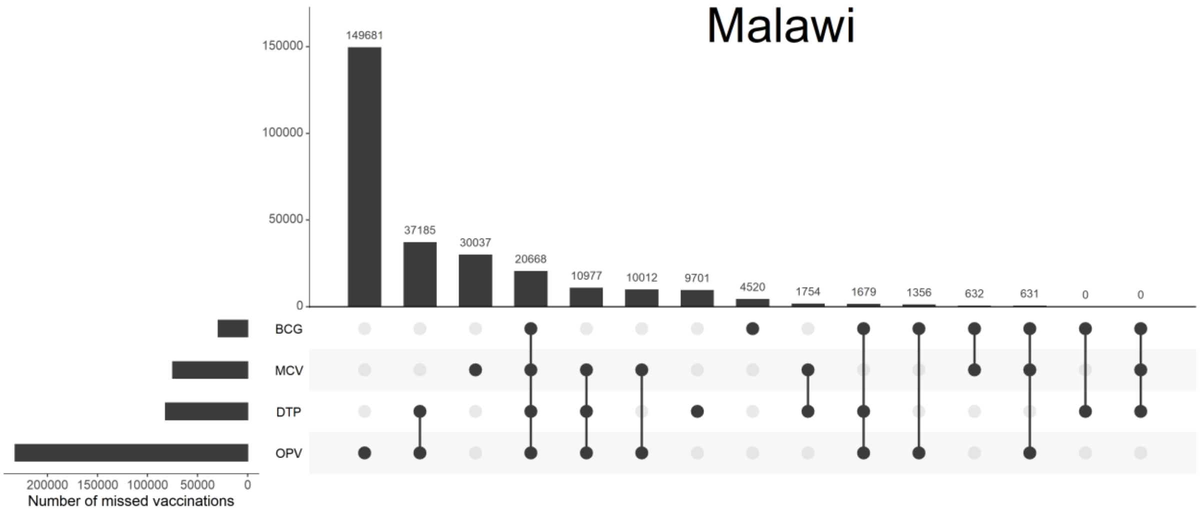
Malawi
149681
37185
30037
20668
10977
10012
9701
4520
1754
1679
1356
632
631
0
0
150000
100000
50000
0
BCG
MCV
DTP
OPV
200000 150000 100000 50000 0
Number of missed vaccinations

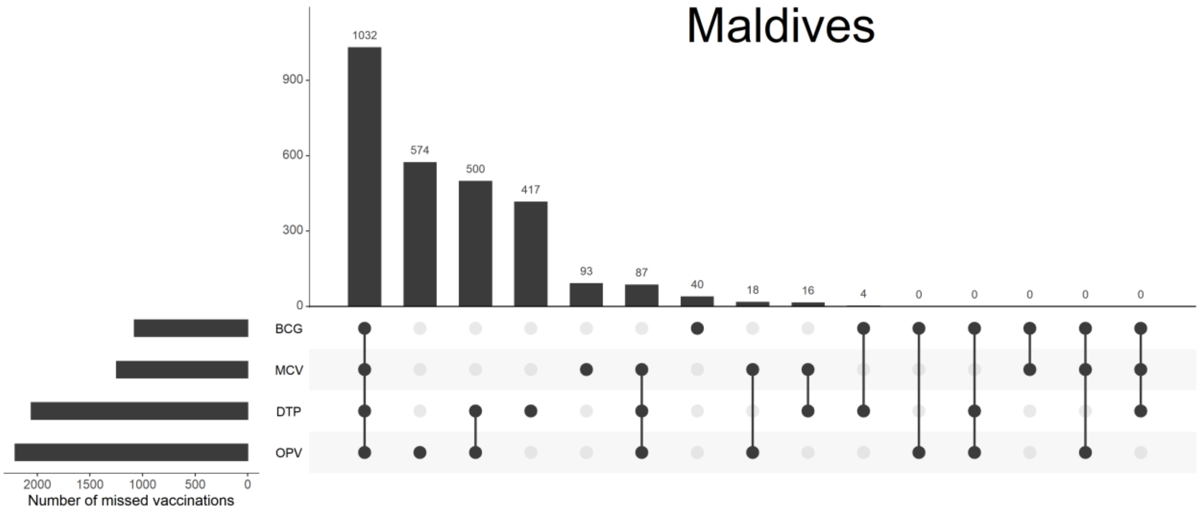
Maldives
1032
574
500
417
93
87
40
18
16
4
0
0
0
0
0
900
600
300
0
BCG
MCV
DTP
OPV
2000 1500 1000 500 0
Number of missed vaccinations

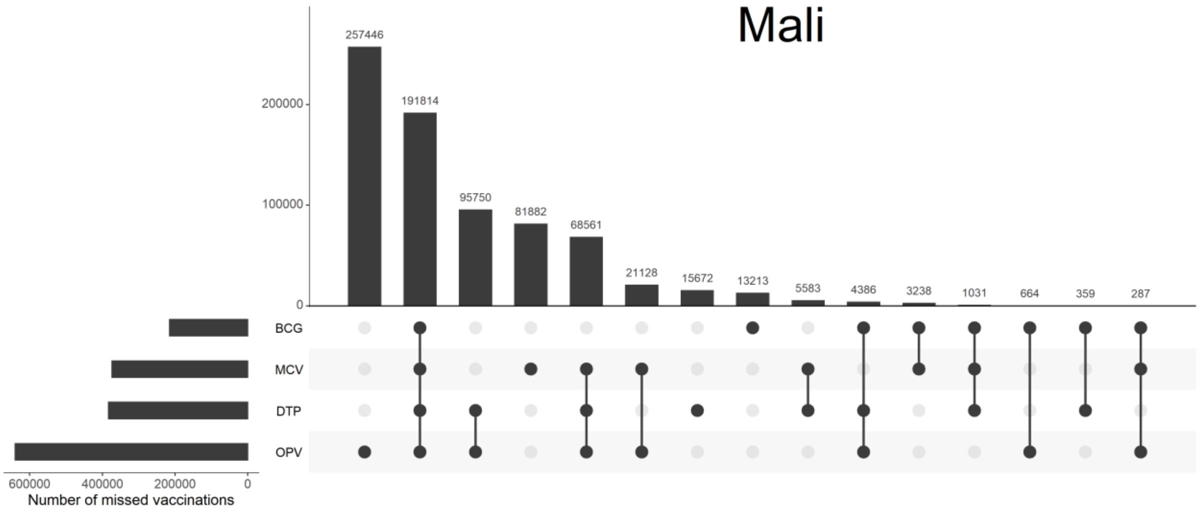
Mali
257446
191814
95750
81882
68561
21128
15672
13213
5583
4386
3238
1031
664
359
287
200000
100000
0
BCG
MCV
DTP
OPV
600000 400000 200000 0
Number of missed vaccinations

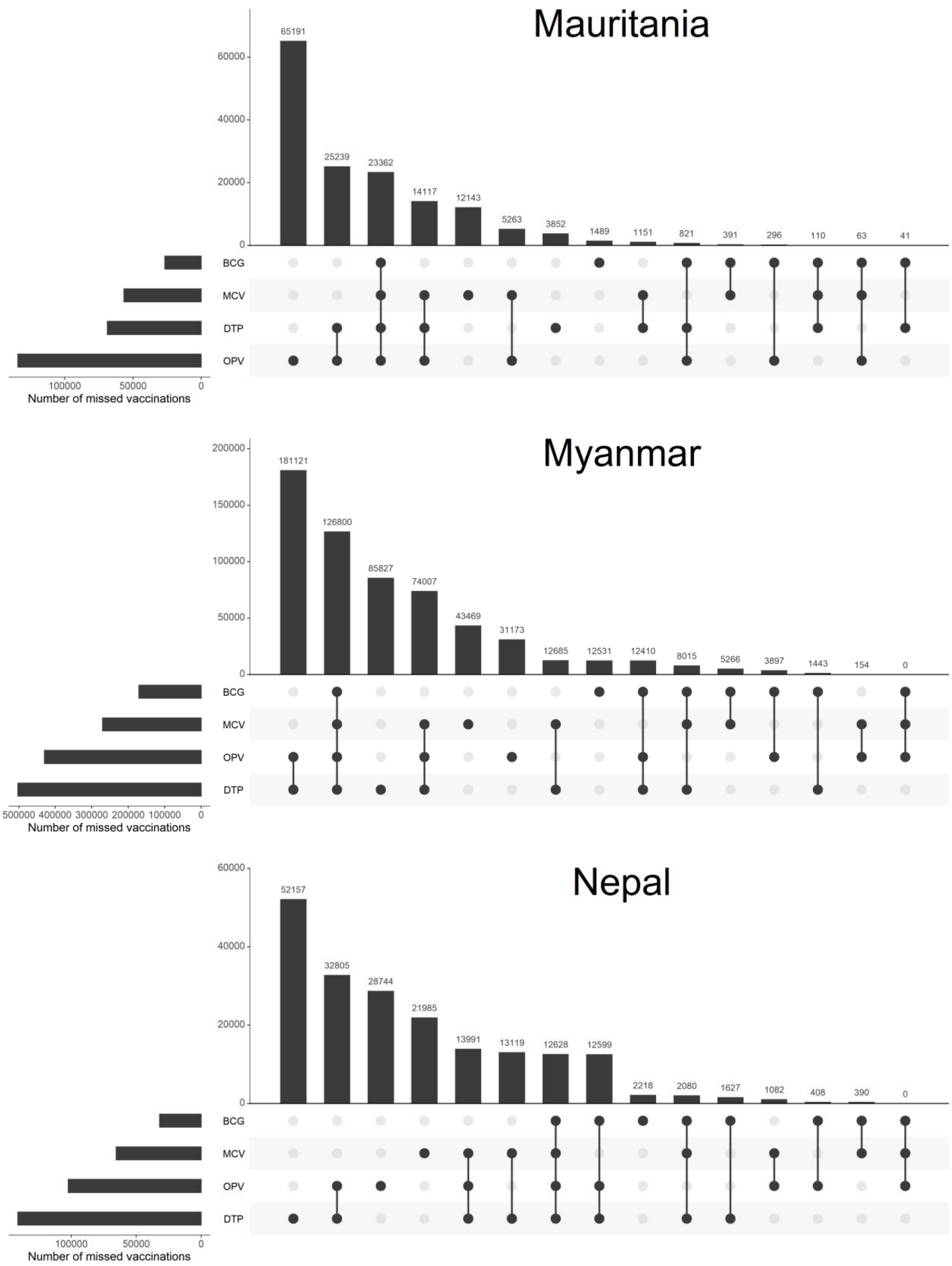
Mauritania
65191
25239
23362
14117
12143
5263
3852
1489
1151
821
391
296
110
63
41
60000
40000
20000
0
BCG
MCV
DTP
OPV
100000
50000
0
Number of missed vaccinations
Myanmar
181121
126800
85827
74007
43469
31173
12685
12531
12410
8015
5266
3897
1443
154
0
200000
150000
100000
50000
0
BCG
MCV
OPV
DTP
500000 400000 300000 200000 100000 0
Number of missed vaccinations
Nepal
52157
32805
28744
21985
13991
13119
12628
12599
2218
2080
1627
1082
408
390
0
60000
40000
20000
0
BCG
MCV
OPV
DTP
100000
50000
0
Number of missed vaccinations

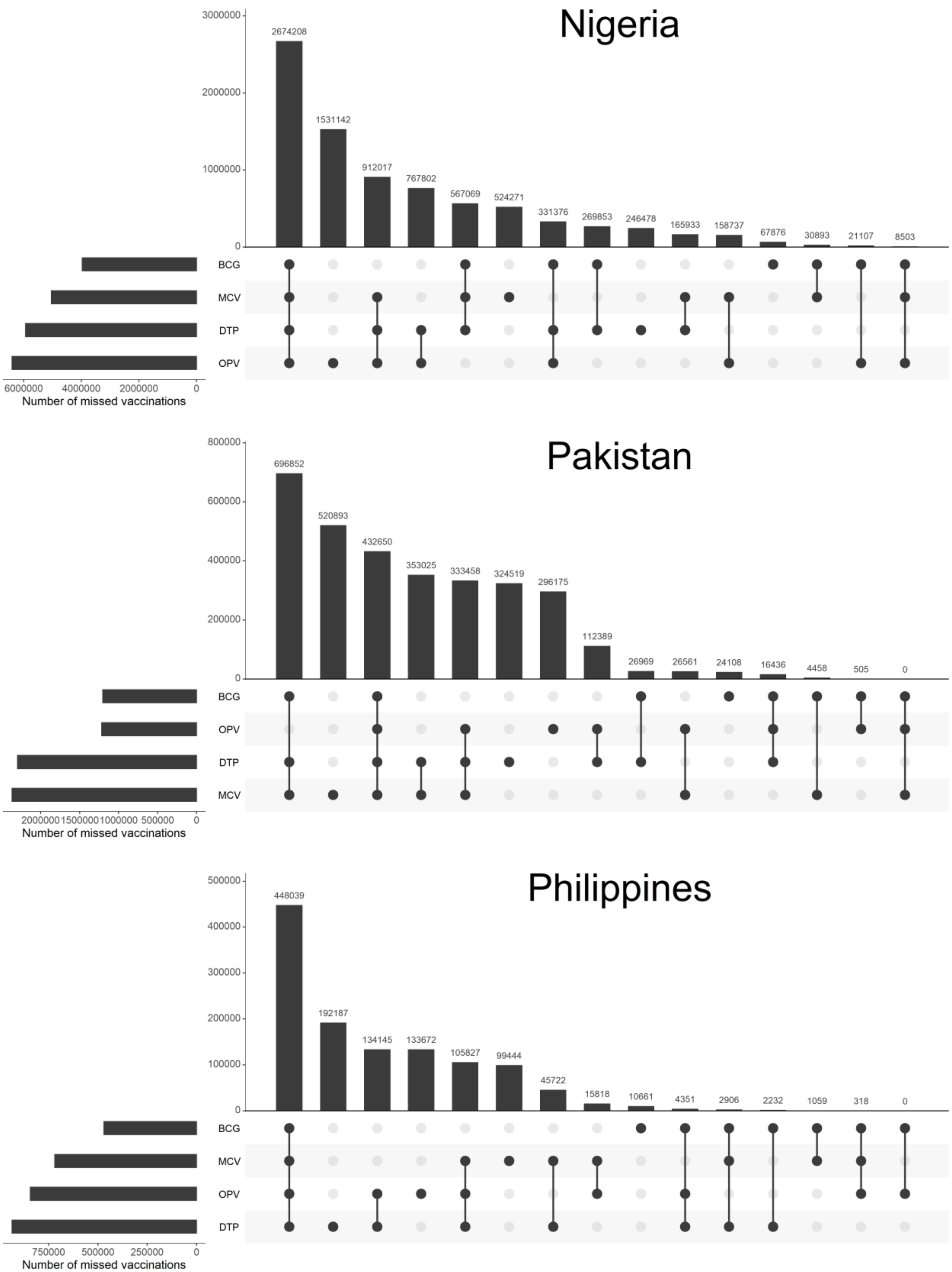
Nigeria
3000000
2000000
1000000
0
2674208
1531142
912017
767802
567069
524271
331376
269853
246478
165933
158737
67876
30893
21107
8503
BCG
MCV
DTP
OPV
6000000 4000000 2000000 0
Number of missed vaccinations
Pakistan
800000
600000
400000
200000
0
696852
520893
432650
353025
333458
324519
296175
112389
26969
26561
24108
16436
4458
505
0
BCG
OPV
DTP
MCV
2000000 1500000 1000000 500000 0
Number of missed vaccinations
Philippines
500000
400000
300000
200000
100000
0
448039
192187
134145
133672
105827
99444
45722
15818
10661
4351
2906
2232
1059
318
0
BCG
MCV
OPV
DTP
750000 500000 250000 0
Number of missed vaccinations

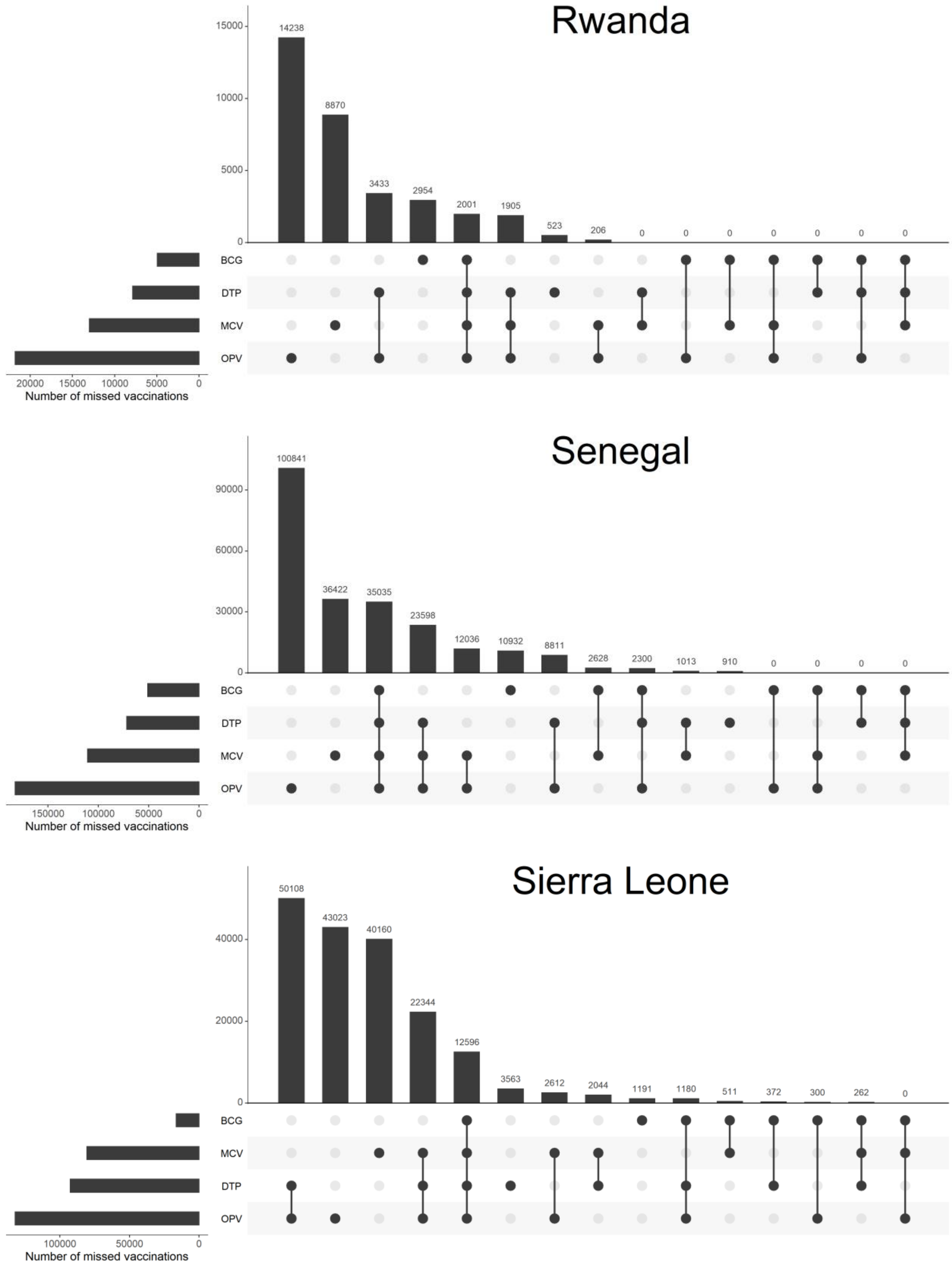
Rwanda
15000
10000
5000
0
14238
8870
3433
2954
2001
1905
523
206
0
0
0
0
0
0
0
BCG
DTP
MCV
OPV
20000 15000 10000 5000 0
Number of missed vaccinations
Senegal
90000
60000
30000
0
100841
36422
35035
23598
12036
10932
8811
2628
2300
1013
910
0
0
0
0
BCG
DTP
MCV
OPV
150000 100000 50000 0
Number of missed vaccinations
Sierra Leone
40000
20000
0
50108
43023
40160
22344
12596
3563
2612
2044
1191
1180
511
372
300
262
0
BCG
MCV
DTP
OPV
100000 50000 0
Number of missed vaccinations

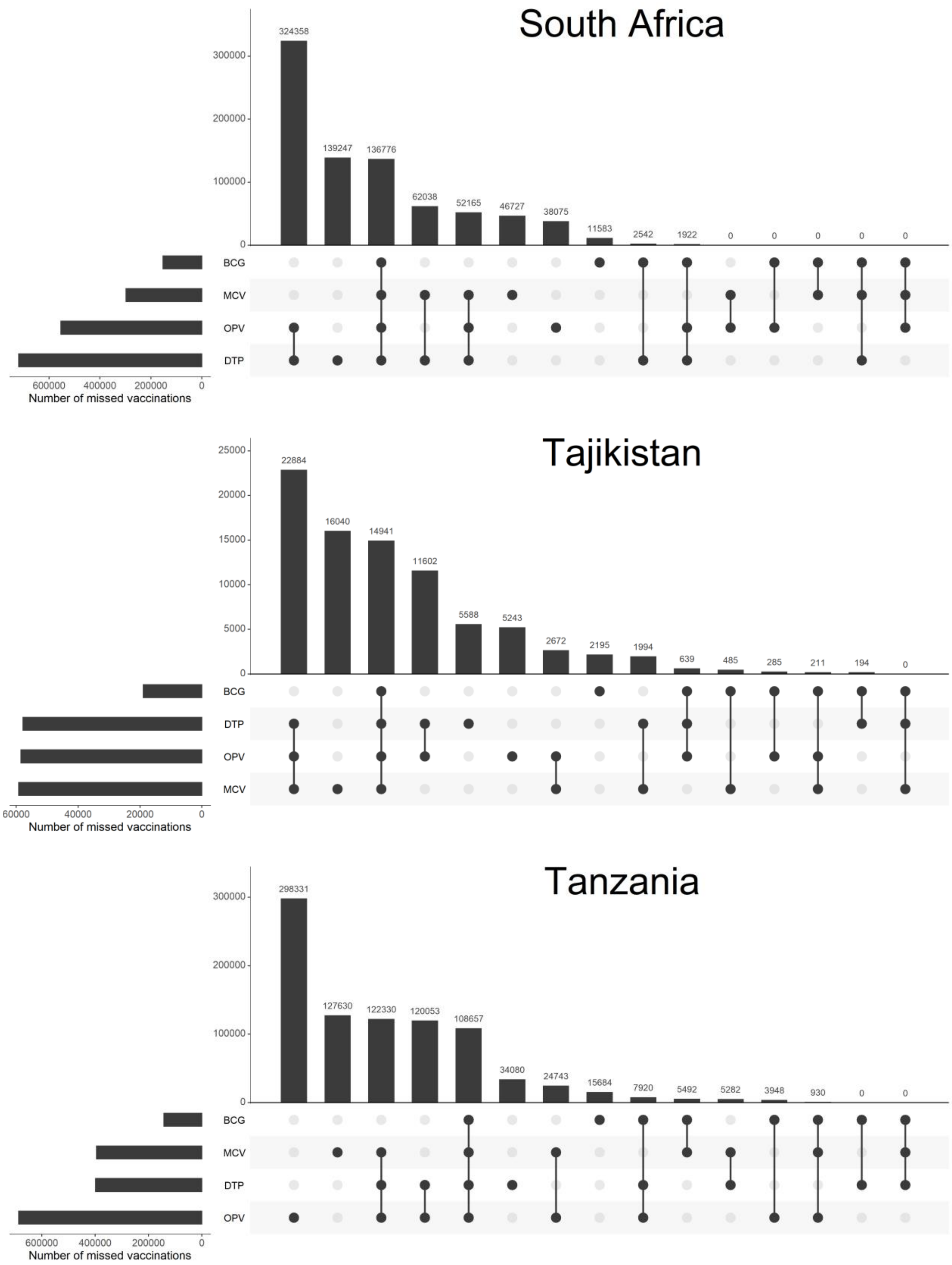
South Africa
324358
139247
136776
62038
52165
46727
38075
11583
2542
1922
0
0
0
0
0
300000
200000
100000
0
BCG
MCV
OPV
DTP
600000
400000
200000
0
Number of missed vaccinations
Tajikistan
22884
16040
14941
11602
5588
5243
2672
2195
1994
639
485
285
211
194
0
25000
20000
15000
10000
5000
0
BCG
DTP
OPV
MCV
60000
40000
20000
0
Number of missed vaccinations
Tanzania
298331
127630
122330
120053
108657
34080
24743
15684
7920
5492
5282
3948
930
0
0
300000
200000
100000
0
BCG
MCV
DTP
OPV
600000
400000
200000
0
Number of missed vaccinations

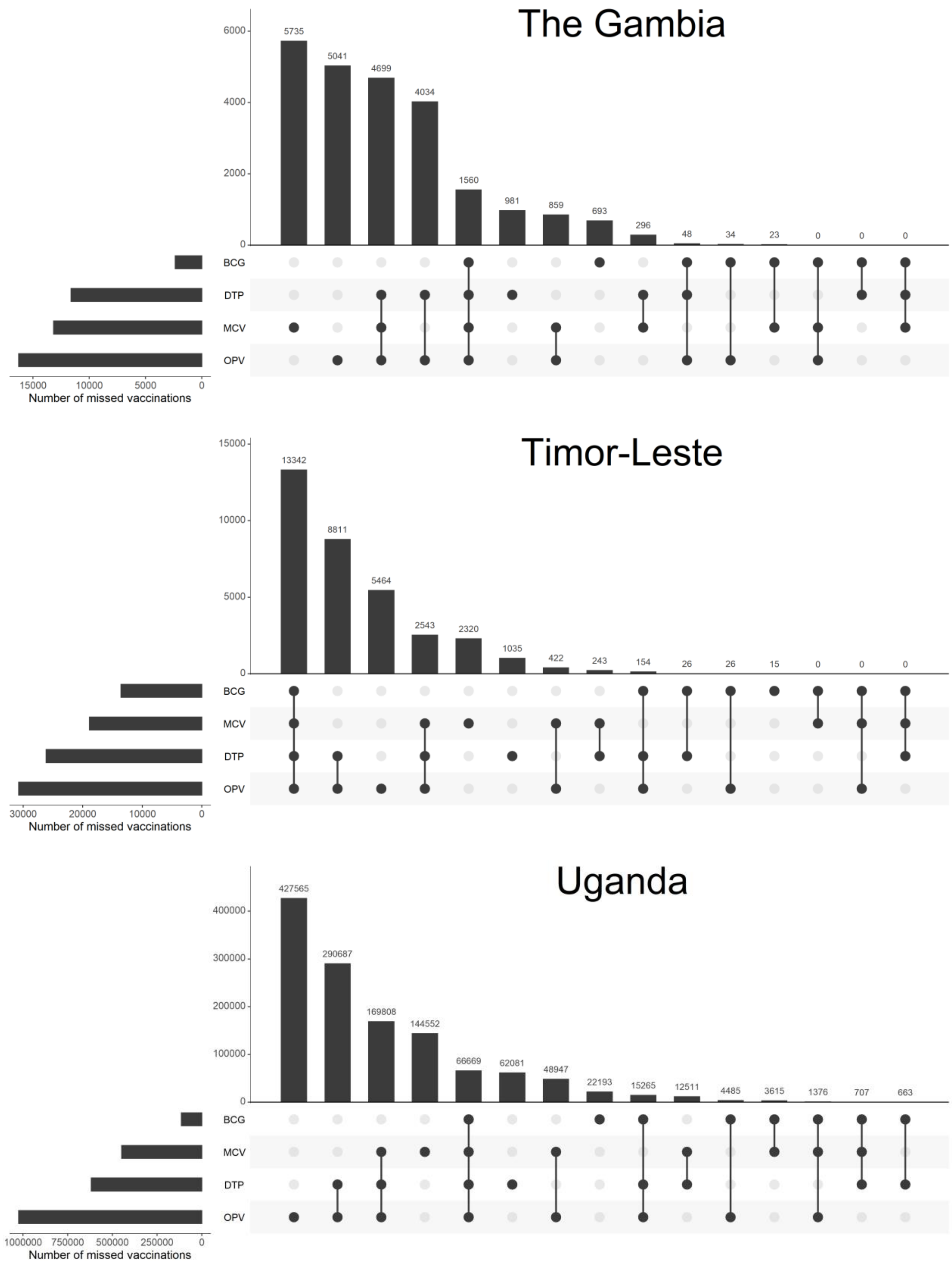

The Gambia
5735
5041
4699
4034
1560
981
859
693
296
48
34
23
0
0
0
BCG
DTP
MCV
OPV
Number of missed vaccinations
Timor-Leste
13342
8811
5464
2543
2320
1035
422
243
154
26
26
15
0
0
0
BCG
MCV
DTP
OPV
Number of missed vaccinations
Uganda
427565
290687
169808
144552
66669
62081
48947
22193
15265
12511
4485
3615
1376
707
663
BCG
MCV
DTP
OPV
Number of missed vaccinations

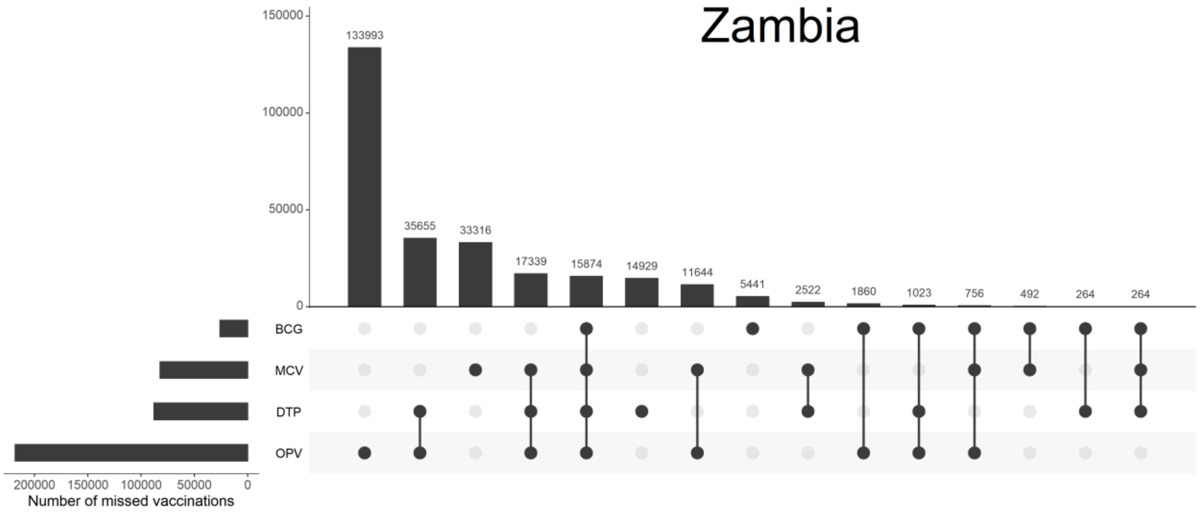
Zambia
150000
100000
50000
0
133993
35655
33316
17339
15874
14929
11644
5441
2522
1860
1023
756
492
264
264
BCG
MCV
DTP
OPV
200000 150000 100000 50000 0
Number of missed vaccinations

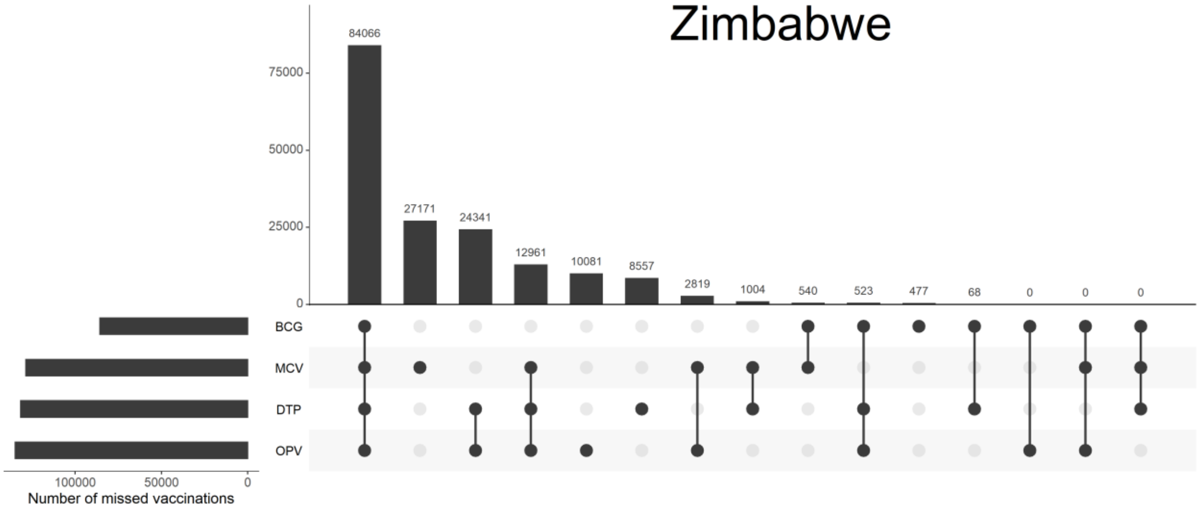
Zimbabwe
75000
50000
25000
0
84066
27171
24341
12961
10081
8557
2819
1004
540
523
477
68
0
0
0
BCG
MCV
DTP
OPV
100000 50000 0
Number of missed vaccinations
