# Supplementary material for: Essential childhood immunization in 43 low- and middle-income countries: Analysis of spatial trends and socioeconomic inequalities in vaccine coverage
Source: PLoS Med. 2023 Jan 17;20(1):e1004166. doi: 10.1371/journal.pmed.1004166 (PMC9888726; doi:10.1371/journal.pmed.1004166)
Supplement: Attachment — Submitted filename: dimitrova.pdf [file pmed.1004166.s013.pdf]

This paper uses an established set of surveys to examine childhood immunisation in low– and middle–income countries.

I was asked for a statistical report and I interpret that to include all aspects of the design and conduct of the study.

Points of detail

Page 6 Typo, principal not principle.

Page 7 I think the authors are assuming rather mtoo much background knowledge about their chosen measure of inequality. There are of course many indexes of concentration and I suspect most readers of this journal will immediately think of the Gini index. The authors' choice seems to be a bivariate index of health status and wealth so I think we need more explanation here or in supplementary material.

Page 7 Providing a proper citation for the package used would be good practice `https://cran.r-project.org/package=UpSetR` should work or do `citation("UpSetR")`. It is the only reward authors get for providing packages.

Page 8 I imagine queen here means rook+bishop but again I feel the authors assume rather more knowledge of the technology they are using than many readers will have. This section could be expanded or re–expressed.

Page 9 Typo 'low very' = 'very low'?

Page 14 Caption could explain what the dots mean. Do they mean that immunisation was missed or completed? There are only 15 sets in the figure not the $2^4 = 16$ one might expect. If including the missing one distorts the scale too much then at least tell us in the caption which it is and its relative frequency.

Supplementary Figure S3 The vaccines are not listed in the same order for each country which makes comparison between them extremely hard.

Points of more substance

The maps

The authors have for some of the maps (Figures 1 and 3) decided to provided an inset of Central America and the Caribbean. This seems to me a good idea but I would suggest the impact of the maps would be improved by providing an inset for that part of Europe and Asia which contains Albania and Armenia. At the moment they are too insignificant. Space could be made by removing all the Americas apart from the inset. I leave it to the authors whether to split Africa and the rest of Asia and Oceania into separate panels. I would make all the maps consistent, I am not sure why the inset disappears from some of them.

What exactly do the clusters mean?

More specifically what inference am I expected to draw from them? What influence does it have that the units mentioned in the Supplementary Table S2 seem to be of varying size for different countries? For instance Ethiopia and Gambia have similar numbers of units (11 v 8) but Ethiopia is about 100 times bigger than The Gambia and has about 50 times the population. I have very limited experience of Demographic Surveillance Sites (DSS) but the only one I have visited in Ethiopia (Butajira) is quite small and if that is supposed to represent the whole of the Southern Nations, Nationalities, and Peoples' Region then I am sceptical. If there is only one DSS in Oromia then I find that even more implausible. I suspect this issue means that finding clusters in a country like the Gambia one will be artefactually easier.

Summary

Putting the data on missing vaccinations in front of the readership is clearly a good idea but I need convincing about the merits of the spatial analysis.

Michael Dewey
