# Supplementary material for: Essential childhood immunization in 43 low- and middle-income countries: Analysis of spatial trends and socioeconomic inequalities in vaccine coverage
Source: PLoS Med. 2023 Jan 17;20(1):e1004166. doi: 10.1371/journal.pmed.1004166 (PMC9888726; doi:10.1371/journal.pmed.1004166)
Supplement: Attachment — Submitted filename: Response to reviewers.docx [file pmed.1004166.s014.docx]

Academic Editor:

*1) My main concern is lack of representativeness and power to support inference of the small area units used for spatial analysis, as pointed out by several reviewers as well. These are further divided by examining inequalities within those small regions (i.e., even fewer individuals per wealth group). The uncertainty estimates can only be very high here and this should be more explicitly discussed in limitations--as well as in sensitivity analyses, say dropping the smallest units. Some of the maps are not effective given the relatively few countries/areas in the analysis--see Figure 4. Otherwise, this is a fine paper though not entirely novel.*

**Response:** We would like to thank the editor for the constructive comments and suggestions. Concerning the uncertainty underlying our analysis, we have added 95% confidence intervals for the inequality indices in the new Table 1. Additionally, in Supplementary Table S5 we included 95% for the subnational estimates.

Concerning the representativeness of DHS samples at the subnational level, in the revised manuscript we have described in more details the data collection procedure, which involves a two-stage sampling design, see page 6, lines 104-106:

“The sampling procedure ensures that the samples are representative both at national and subnational levels (usually administrative level 1 or 2, e.g. region or province) and by urban/rural area.”

When preparing subnational estimates, we followed the DHS sample design for each country and aggregated the data at the subnational level at which they are representative (administrative level 1 or 2). Additionally, in all calculations we included sampling weights.

To quantify wealth inequalities, we calculate the concentration index using individual immunization and wealth information. We do not compare wealth-group aggregates in immunization coverage but use the wealth index, which is on a continuous scale, to rank individuals from the poorest to the wealthiest. Therefore, our estimates are not sensitive to the sample size of wealth groups as long as the wealth distribution within the sample is representative of the general population. What matters is the relative position of individual children within the wealth distribution in the specific country/region. We provided more information on this in the methods section on pages 8 and 9 and provided an illustration of the concentration curve underlying the inequality analysis in Supplementary Figure S2.

To communicate better the uncertainty underlying the subnational estimates, we plotted the subnational estimates that are statistically significant at 95 percent confidence level in Supplementary Figure S3. The figure shows that in administrative regions where the highest inequality is observed, the estimates are highly statistically significant. We additionally improved the maps by providing panels that zoom into small areas.

Finally, in the revised manuscript, we updated the data, retrieving the latest available DHS surveys. Two countries were added to the analysis (Madagascar and Mauritania) and the data for India were updated with the latest survey wave from 2019-2021.

*2) Please revise your title according to PLOS Medicine's style. Your title must be nondeclarative and not a question. It should begin with main concept if possible.  Please place the study design (for example, "Spatial cluster analysis”) in the subtitle (ie, after a colon).*

**Response:** We have revised the title of the paper according to the PLOS Medicine style guidelines.

*3) Abstract:*

*a) Please structure your abstract using the PLOS Medicine headings (Background, Methods and Findings, Conclusions).*

*b) Please include the actual amounts of relevant outcomes. Please provide the key estimates and confidence intervals in the Methods and Finding section*

*c) Please include the important dependent variables that are adjusted for in the analyses.*

*d) In the last sentence of the Abstract Methods and Findings section, please describe the main limitation(s) of the study's methodology.*

**Response:** We have restructured the abstract in accordance with the PLOS Medicine guidelines.

*4) Author summary - At this stage, we ask that you include a short, non-technical Author Summary of your research to make findings accessible to a wide audience that includes both scientists and non-scientists. The Author Summary should immediately follow the Abstract in your revised manuscript. This text is subject to editorial change and should be distinct from the scientific abstract. Please see our author guidelines for more information:*

[*https://urldefense.com/v3/__https://journals.plos.org/plosmedicine/s/revising-your-manuscript*loc-author-summary__;Iw!!Mih3wA!CT9eQuwO0kAK1CqnQCx3ANXHxpENZWk98cSiRgLi5whYav-x1WyN1PiCKF1k7_vD-4SaknXaxXbyzVJ58g$*](https://urldefense.com/v3/__https://journals.plos.org/plosmedicine/s/revising-your-manuscript*loc-author-summary__;Iw!!Mih3wA!CT9eQuwO0kAK1CqnQCx3ANXHxpENZWk98cSiRgLi5whYav-x1WyN1PiCKF1k7_vD-4SaknXaxXbyzVJ58g$) *.*

**Response:** We prepared an Author Summary, following the PLOS Medicine author guidelines.

*5) For streamlining, the last paragraph of the Background can be moved to the discussion section as you begin discussing the implications of the findings of your study.*

**Response:** We have revised the text accordingly.

*6) As alluded to by most reviewers, the novelty aspect of this paper needs further elaboration and justification.*

**Response:** We have emphasized the novelty aspects of the study, for example on page 5, lines 82-84:

“We add to the literature by presenting harmonized and spatially disaggregated estimates of full immunization coverage and wealth-related inequalities across multiple LMICs.”

*7) Please remove the “Role of the funding source” statement from the Methods. In the event of publication, this information will be published as metadata based on your responses to the submission form.*

**Response:** In the revised version of the manuscript, we have removed the “Role of funding sources” section.

*8) Please ensure that the study is reported according to the STROBE guideline or similar, and include the completed STROBE checklist as Supporting Information. When completing the checklist, please quote section and paragraph numbers rather than page numbers. Please add the following statement, or similar, to the Methods: "**This study is reported as per the Strengthening the Reporting of Observational Studies in Epidemiology (STROBE) guideline (S1 Checklist)."*

**Response:** We have ensured that the STROGE guideline is followed and included a STROBE checklist as Supporting Information.

*9) Please temper claims of primacy in abstract and main text (e.g., “we provide the first global analysis”) by stating, "to our knowledge" or something similar.*

**Response:** The manuscript was revised accordingly.

*10)  For your Tables and figures, please do the following:*

*a) In figure 1 and 3, please indicate in the figure caption the meaning of the bars and whiskers*

*b) Please define the abbreviations such as MCV, DTP, OPV, BCG*

*c) Please confirm that the appropriate usage rights apply to the use of this map. Please see our guidelines for map images:*

[*https://urldefense.com/v3/__https://journals.plos.org/plosmedicine/s/figures*loc-maps__;Iw!!Mih3wA!CT9eQuwO0kAK1CqnQCx3ANXHxpENZWk98cSiRgLi5whYav-x1WyN1PiCKF1k7_vD-4SaknXaxXY_PCrBfA$*](https://urldefense.com/v3/__https:/journals.plos.org/plosmedicine/s/figures*loc-maps__;Iw!!Mih3wA!CT9eQuwO0kAK1CqnQCx3ANXHxpENZWk98cSiRgLi5whYav-x1WyN1PiCKF1k7_vD-4SaknXaxXY_PCrBfA$)

**Response:** In the revised manuscript, we removed the bar figures and presented the information in a table instead. Abbreviations were defined in all figures and tables where such abbreviations were included. We made sure that the usage rights were respected in all maps and referenced all data sources.

*11) References:*

*a) Please select the PLOS Medicine reference style in your citation manager. In-text reference call outs should be presented as follows noting the absence of spaces within the square brackets, e.g., “…population [1,2].”*

*b) Please ensure that journal name abbreviations match those found in the National Center for Biotechnology Information (NCBI) databases and are appropriately formatted and capitalized.* [*https://urldefense.com/v3/__https://journals.plos.org/plosmedicine/s/submission-guidelines*loc-references__;Iw!!Mih3wA!CT9eQuwO0kAK1CqnQCx3ANXHxpENZWk98cSiRgLi5whYav-x1WyN1PiCKF1k7_vD-4SaknXaxXY2Crw1rA$*](https://urldefense.com/v3/__https:/journals.plos.org/plosmedicine/s/submission-guidelines*loc-references__;Iw!!Mih3wA!CT9eQuwO0kAK1CqnQCx3ANXHxpENZWk98cSiRgLi5whYav-x1WyN1PiCKF1k7_vD-4SaknXaxXY2Crw1rA$) *.*

*c) Please ensure that weblinks are current and accessible to date.*

**Response:** In the revised manuscript, we used the PLOS Medicine reference style, ensuring that the journal name abbreviations match the NCBI database and that weblinks are active.

# Reviewer 1:

*The paper used the most recent available Demographic Health Surveys (DHS) to assess spatial and socioeconomic inequalities in child immunization in 40 low- and middle-income countries (LMICs). The authors provide national and subnational statistics about vaccination rates for the countries included in the study. Additionally, the authors used the concentration index approach to report wealth-related inequalities in vaccination uptake in the countries. To better present the finding, the paper uses special illustration techniques to better present areas* *with higher/lower vaccination and socioeconomic inequalities in LMICs.*

**Response:** We thank the reviewer for the constructive comments and suggestions, which have helped us greatly to improve the manuscript.

*12) Although the paper addresses an important health issue, the literature review provided in the study is not comprehensive. The authors claim that this study "provide the first global analysis of spatial and socioeconomic inequalities in essential vaccine coverage." The paper also states that "no global assessment has been carried out to date based on the full EPI schedule for young children". These statements are incorrect as there are recent studies that measured and even explained socioeconomic inequalities in vaccination uptake in LMICs. For example, see*

[*https://urldefense.com/v3/__https://www.ncbi.nlm.nih.gov/pmc/articles/PMC5096343/__;!!Mih3wA!CT9eQuwO0kAK1CqnQCx3ANXHxpENZWk98cSiRgLi5whYav-x1WyN1PiCKF1k7_vD-4SaknXaxXbG-FkPAw$*](https://urldefense.com/v3/__https://www.ncbi.nlm.nih.gov/pmc/articles/PMC5096343/__;!!Mih3wA!CT9eQuwO0kAK1CqnQCx3ANXHxpENZWk98cSiRgLi5whYav-x1WyN1PiCKF1k7_vD-4SaknXaxXbG-FkPAw$)

[*https://urldefense.com/v3/__https://jech.bmj.com/content/72/8/719__;!!Mih3wA!CT9eQuwO0kAK1CqnQCx3ANXHxpENZWk98cSiRgLi5whYav-x1WyN1PiCKF1k7_vD-4SaknXaxXZAPywh0Q$*](https://urldefense.com/v3/__https:/jech.bmj.com/content/72/8/719__;!!Mih3wA!CT9eQuwO0kAK1CqnQCx3ANXHxpENZWk98cSiRgLi5whYav-x1WyN1PiCKF1k7_vD-4SaknXaxXZAPywh0Q$)

[*https://urldefense.com/v3/__https://www.sciencedirect.com/science/article/pii/S0749379720303950__;!!Mih3wA!CT9eQuwO0kAK1CqnQCx3ANXHxpENZWk98cSiRgLi5whYav-x1WyN1PiCKF1k7_vD-4SaknXaxXYZz1udXQ$*](https://urldefense.com/v3/__https://www.sciencedirect.com/science/article/pii/S0749379720303950__;!!Mih3wA!CT9eQuwO0kAK1CqnQCx3ANXHxpENZWk98cSiRgLi5whYav-x1WyN1PiCKF1k7_vD-4SaknXaxXYZz1udXQ$)

*The paper does not mention any of these papers. The authors need to conduct a comprehensive literature review and highlight the main contribution of their study given the relevant studies on this topic.*

**Response:** We thank the reviewer for the suggested literature. We expanded the literature overview and discussed our findings in the wider context. The studies suggested by the reviewers were referenced in the paper.

*13) The paper does poorly when it explains its data source. There should be further information about the DHS. For example, one may wonder what the response rate of DHS for each country is. How the data have been collected?*

**Response:** In the revised methods section, we have included a detailed description of the DHS data collection procedure. For example, on page 6, lines 96-111:

“The survey design is based on a two-step sampling procedure [23]. In the first stage, probability samples are drawn from an existing sample frame, usually the most recent census. The sampling frame is divided into subgroups (strata), which are typically geographic regions and urban/rural areas within each region. Within each stratum, primary sampling units (PSUs) are selected with probability proportional to the size within each stratum. In the second stage, a complete household listing is conducted in each PSU and a fixed number of households is selected via systematic sampling. Sampling weights are provided to adjust for differences in the probability of selection and interview. Supplementary Fig S1 shows the location of PSUs in each country included in the analysis. The sampling procedure ensures that the samples are representative both at national and subnational levels (usually administrative level 1 or 2, e.g. region or province) and by urban/rural area. Detailed description of data collection and validation procedures are described elsewhere [23,24]. Key strengths of the DHS surveys are the high response rates (usually over 90%), their national coverage, high quality of interviewer training and the standardised data collection procedures, which allow comparisons across countries and over time [25]. The DHS surveys have become a valuable source of information in epidemiological research, with a wide range of applications for monitoring of prevalence, trends, and inequalities.”

*14) It is unclear whether the paper considered the survey design in their estimation. For example, did the authors used sampling weight in the analyses?*

**Response:** Sampling weights were included in all estimation procedures. We provided more explicit information about the survey design (see our response to the above comment) and clarified the use of sampling weights in all analyses. For example, on page 9, lines 174-177:

“For the purpose of our analysis, we use both the Wagstaff’s (W) and Erreygers’ (E) adjusted concentration indices to measure socioeconomic inequalities in childhood immunization. The “conindex” package [41] in Stata 16 was used to estimate W and E. Sampling weights were applied in the calculation of both indices.”

*15) If the MCV should be administrated by 9 months, why does the study restrict its sample to the children 12-35 month?*

**Response:** In the revised manuscript, we decided to restrict the analysis to children between 15 and 35 months of age due to the differences in vaccination schedules across countries. We cross-checked the vaccination schedules for each country included in the analysis with the most up to date WHO/UNICEF reports (<https://immunizationdata.who.int/pages/schedule-by-disease/measles.html>). As it turned out, in a few countries (Albania, Armenia, Egypt, Guatemala, India and Jordan), measles vaccines are administered up to 12 or 13 months of age. This implies that full immunization coverage would be underestimated there in the age group 12-35 months. For comparability purposes and to allow for some catch up vaccination, we provided estimates for children 15 to 35 months of age instead. In a robustness analysis, we estimated vaccination rates for the age group 24-35 and the results were comparable.

*16) The existing literature on socioeconomic inequalities in health suggests using the Wagstaff index and the Erreygers Index when we measure socioeconomic inequalities for binary health outcomes. One may wonder why the paper only used the Wagstaff index in their study.*

**Response:** We thank the reviewer for the suggestion to use Erreygers’ index of inequality. In the revised manuscript, we included both Wagstaff’s and Erreygers’ indices.
 *17) The authors can provide further information on variation in vaccination rates using summary inequality measures such as the Theil index (T), between-group variance (BGV).*

**Response:** We thank the reviewer for making this suggestion. We calculated the Theil index for each vaccine separately, for the full immunization rate, and also for the inequality indices. We provided estimates of between- and within-country disparities.

*18) On page 11, what range of the C was used in their following statement, "low versus high socioeconomic inequalities in childhood immunization status"?*

**Response:** We clarified the ranges for which the degree of socioeconomic inequality in childhood immunization is considered to be low. We revised the text on page 14, lines 256-260 as follows:

“Most countries display low degree of socioeconomic inequality (absolute values of W and E between 0 and 0.15; Table 1). However, three countries stand out with a more considerable degree of inequality – Angola, Nigeria and Pakistan – with both W and E values of 0.3 or higher, implying that vaccination there is concentrated among the wealthier groups.”

# Reviewer 2:

*Overview: In this manuscript, the authors conduct a series of secondary analyses of DHS data to explore patterns of full immunization coverage and their intersections with socioeconomic status in the most recent DHS surveys for 40 low- and middle-income countries. Similar data for some of the results presented here are already available from standard DHS reports and resources online; for instance, subnational full-vaccination coverage among 12-23 month-olds is a standard component of DHS reports; here, the authors use 12-35 months as the age group of analysis instead. In this manuscript, however, the authors compile these indicators across multiple countries for easier comparison and use standard geospatial analytic techniques to identify clusters of high and low coverage. Multiple manuscripts have applied these sorts of methods in the past, but usually for single countries or larger groups of countries; the novelty in this part of the analysis comes primarily from its application across a relatively large number of countries.*

*In addition, however, the authors also look at subnational patterns of socioeconomic inequalities in full vaccinated coverage. This interesting analysis provides insight into the ways in which two different categories of vaccination inequality (spatial and socioeconomic) intersect within countries. They also analyze the specific patterns of missing vaccines within and across countries - i.e. if a child is not fully vaccinated, which vaccine(s) are most likely to be incomplete? In all, the manuscript is clearly written and provides several useful additions to the accumulated body of literature that uses DHS data to analyze patterns of vaccination coverage. I have a few major comments (below) and several minor comments for the authors' consideration, provided in the hope that they will further strengthen the manuscript.*

**Response:** We appreciate the constructive comments and suggestion provided by the reviewer, which have helped us greatly to improve the manuscript.

*19) In general, the manuscript would greatly benefit from more attention given to the uncertainty inherent in these analyses. While DHS surveys are designed to be representative at these subnational spatial scales, smaller sample sizes generally mean that the uncertainty around subnational estimates generated using traditional survey statistics for these subnational units may be wider. Did the authors use only mean estimates of coverage in all of these analyses, or was uncertainty accounted for in some way? Could the authors provide 95% CI or UI ranges for figures cited, i.e. in lines 232-236 and elsewhere? In particular, it would be useful to know how much uncertainty there is in the subnational estimates of full immunization rates and concentration index values.*

**Response:** We thank the reviewer for drawing attention to this issue. In the revised manuscript we provided uncertainty estimates for the concentration indices both at the national and subnational level (95% confidence intervals are provided in Table 1 at the national level and Supplementary Table S5 at the subnational level). We additionally provided maps where only statistically significant values were shown at the subnational level (Supplementary Figure S3), for easier interpretation. We have not provided uncertainty estimates for full immunization coverage since this statistic is used for descriptive purposes only. However, we included a detailed description of the DHS data collection procedure on page 5 and 6, lines 94-109, which ensures that samples are representative both at the national and subnational level.

*20) The manuscript also should contain a thorough discussion of the limitations of this analysis and the results. Unless I am missing it, this is not currently present in the discussion section. There are a few places where caveats are given - for instance, in lines 149-152, the authors note the (rather major) assumption that coverage hasn't changed since the last DHS survey for each country. The fact that the included DHS surveys took place over a range of 5 years, and that some of these surveys were not conducted almost 8 years ago, limits both comparability of the results and inference about the global findings. This and other limitations - a few of which I have remarked on below, but the authors likely would want to comment on others - should be discussed in some detail.*

**Response:** We thank the reviewer for pointing out that limitations were not thoroughly discussed in the paper. In the revised manuscript, we added a limitations section (pages 20-21, lines 358-373), where the limitations mentioned by the reviewer and other limitations were discussed.

*21) Line 104-106: The authors used a standard EPI schedule to set the 12-35 month time frame for vaccination. This makes sense for BCG, DTP and OPV, all of which are recommended for administration in the first few weeks or months of life. The schedule for measles, however, is variable, depending on countries' measles epidemiology, etc.*

*(*[*https://urldefense.com/v3/__https://immunizationdata.who.int/pages/schedule-by-disease/measles.html?ISO_3_CODE=&TARGETPOP_GENERAL=__;!!Mih3wA!CT9eQuwO0kAK1CqnQCx3ANXHxpENZWk98cSiRgLi5whYav-x1WyN1PiCKF1k7_vD-4SaknXaxXbpitlb0A$*](https://urldefense.com/v3/__https:/immunizationdata.who.int/pages/schedule-by-disease/measles.html?ISO_3_CODE=&TARGETPOP_GENERAL=__;!!Mih3wA!CT9eQuwO0kAK1CqnQCx3ANXHxpENZWk98cSiRgLi5whYav-x1WyN1PiCKF1k7_vD-4SaknXaxXbpitlb0A$) *).*

*How did the authors take this into account, as this is likely to affect comparability of results for children who are at the younger end of this age range?*

**Response:** We thank the reviewer for making this point. We cross-checked the country-specific vaccination schedules with the WHO/UNICEF reports and indeed in six countries included in the analysis (Albania, Armenia, Egypt, Guatemala, India and Jordan), measles vaccines are administered up to 12 or 13 months of age. To account for these differences in vaccination schedules and to allow for some vaccination catchup, we decided to change the reference group to children between 15 and 35 months of age. As a robustness check, we analyzed vaccination rates among 24–35-month-old children (Supplementary Tables S3 and S4) and the results were comparable.

*22) Also for MCV, were the authors able to distinguish between vaccines given via routine immunization services vs campaigns? This is often a challenge when working with DHS data, particularly in cases where one needs to rely on parental recall.*

**Response:** We have not been able to distinguish between vaccines administered as part of routine immunization services versus immunization campaigns due to lack of data. For majority of children included in the analysis vaccination cards were presented during the interview (67%). For the rest, the interviewers relied on parental recall, which is a manor limitation of working with DHS data and we have noted this in the limitations section, pages 20 and 21, lines 364-367:

“The vaccination information was verified via vaccination cards for 67% of children in the sample. For the rest, this information was based on the mother’s recall, which is subject to recall bias. However, the validity of relying on parental reporting of immunization has been verified in the literature [61].”

*23) The authors focus mainly on geographic and socioeconomic inequalities in vaccination coverage in this analysis. Other analyses have focused on the use of individual-level survey data to better understand inequalities, although at aggregate rather than subnational scales. The authors may consider referencing some of these analyses to strengthen and expand their discussion of other potential forms of inequality in vaccination coverage, for instance:*

[*https://urldefense.com/v3/__https://pubmed.ncbi.nlm.nih.gov/35577393/__;!!Mih3wA!CT9eQuwO0kAK1CqnQCx3ANXHxpENZWk98cSiRgLi5whYav-x1WyN1PiCKF1k7_vD-4SaknXaxXbTzUrQNg$*](https://urldefense.com/v3/__https:/pubmed.ncbi.nlm.nih.gov/35577393/__;!!Mih3wA!CT9eQuwO0kAK1CqnQCx3ANXHxpENZWk98cSiRgLi5whYav-x1WyN1PiCKF1k7_vD-4SaknXaxXbTzUrQNg$) *,*

[*https://urldefense.com/v3/__https://pubmed.ncbi.nlm.nih.gov/35356658/__;!!Mih3wA!CT9eQuwO0kAK1CqnQCx3ANXHxpENZWk98cSiRgLi5whYav-x1WyN1PiCKF1k7_vD-4SaknXaxXav_bVAiA$*](https://urldefense.com/v3/__https:/pubmed.ncbi.nlm.nih.gov/35356658/__;!!Mih3wA!CT9eQuwO0kAK1CqnQCx3ANXHxpENZWk98cSiRgLi5whYav-x1WyN1PiCKF1k7_vD-4SaknXaxXav_bVAiA$) *,*

*and/or* [*https://urldefense.com/v3/__https://pubmed.ncbi.nlm.nih.gov/34805814/__;!!Mih3wA!CT9eQuwO0kAK1CqnQCx3ANXHxpENZWk98cSiRgLi5whYav-x1WyN1PiCKF1k7_vD-4SaknXaxXYMlqbpkQ$*](https://urldefense.com/v3/__https:/pubmed.ncbi.nlm.nih.gov/34805814/__;!!Mih3wA!CT9eQuwO0kAK1CqnQCx3ANXHxpENZWk98cSiRgLi5whYav-x1WyN1PiCKF1k7_vD-4SaknXaxXYMlqbpkQ$)*.*

**Response:** We thank the reviewer for the suggested literature. We included a discussion of the various dimension of inequality in vaccination coverage in the Discussion section on page 21, lines 386-391:

“While in this study we have focused on spatial and socioeconomic inequalities in full immunization coverage, other forms of inequalities have also been found in the literature. There is evidence of large disparities in immunization coverage with respect to ethnicity [65], area of residence [17], female empowerment [66], and overall access to primary healthcare services [67], among other factors [8]. Our study complements these findings and emphasizes the importance of monitoring inequalities across multiple dimensions.”

*24) A minor point, but I would suggest that the authors take some care with language describing the scale of the spatial heterogeneities in their analysis. The authors restrict the analysis to the subnational scales at which each DHS survey is representative using traditional survey statistics, i.e. the first administrative level (states, etc.) for some countries, the second administrative level (districts, etc.) for others. (As an aside, the different spatial scales are probably a limitation that is worth mentioning, as it does limit the comparability of some of the clustering and other analyses presented here). In some places, however, the authors refer to their results as exploring "fine-scale spatial heterogeneities". I might suggest that state-level spatial heterogeneities are not truly "fine-scale"; countries routinely use district-level administrative data to guide decision-making, and as the authors note there are a number of research groups (including DHS themselves,*

[*https://urldefense.com/v3/__https://spatialdata.dhsprogram.com/modeled-surfaces/__;!!Mih3wA!CT9eQuwO0kAK1CqnQCx3ANXHxpENZWk98cSiRgLi5whYav-x1WyN1PiCKF1k7_vD-4SaknXaxXZJ2_8WRQ$*](https://urldefense.com/v3/__https:/spatialdata.dhsprogram.com/modeled-surfaces/__;!!Mih3wA!CT9eQuwO0kAK1CqnQCx3ANXHxpENZWk98cSiRgLi5whYav-x1WyN1PiCKF1k7_vD-4SaknXaxXZJ2_8WRQ$) *)*

*who are using geostatistical modeling approaches to estimate gridded or lower-level administrative unit vaccine coverage indicators. The authors might consider ensuring that language describing the spatial scale of the analyses is clear throughout.*

**Response:** We revised the text accordingly and ensured appropriate usage of language. We also included the differences in spatial scales across countries in the limitations section on page 21, lines 372-374:

“At the subnational level, vaccination coverage is estimated at different administrative levels (e.g., states, provinces, or district), depending on the spatial scales at which the DHS surveys are representative, which limits the detection and comparison of spatial clusters.”

Reviewer 3:

*25) This paper uses an established set of surveys to examine childhood immunisation in low- and middle-income countries. I was asked for a statistical report and I interpret that to include all aspects of the design and conduct of the study.*

**Response:** We would like to thank the reviewer for the valuable comments and suggestions, which have helped us greatly to improve the manuscript.

*26) Page 6 Typo,* *principal not principle.*

**Response:** We corrected the typo.

*27) Page 7 I think the authors are assuming rather too much background knowledge about their chosen measure of inequality. There are of course many indexes of concentration and I suspect most readers of this journal will immediately think of the Gini index. The authors' choice seems to be a bivariate index of health status and wealth so I think we need more explanation here or in supplementary material.*

**Response:** We thank the reviewer for pointing this out. In the revised manuscript, we provided more detailed information about the concentration index and the adjustments procedures applied, on pages 8-9, lines 150-175. We also included a graphical presentation of the concentration index in Supplementary Figure S2. The concentration index is indeed a special case of the Gini index.

*28) Page 7 Providing a proper citation for the package used would be good practice* *https://cran.r-project.org/package=UpSetR should work or do citation("UpSetR"). It is the only reward authors get for providing packages.*

**Response:** As suggested, we provided proper citations of all packages used in the analysis.

*29) Page 8 I imagine queen here means rook+bishop but again I feel the authors assume rather more knowledge of the technology they are using than many readers will have. This section could be expanded or re-expressed.*

**Response:** We improved the section on “Spatial autocorrelation analysis”, explaining in more detail the methods.

*30) Page 9 Typo `low very' = `very low'?*

**Response:** We corrected the typo.

*31) Page 14 Caption could explain what the dots mean. Do they mean that immunisation was missed or completed? There are only 15 sets in the figure not the 2^4^ = 16 one might expect. If including the missing one distorts the scale too much then at least tell us in the caption which it is and its relative frequency.*

**Response:** Following the reviewer’s suggestion, we included explanation of the new Figure 4 in the caption. Concerning the number of combinations (15 instead of 16), we have omitted the combination where no vaccine was missed (children that were fully immunized), since we wanted to focus on unvaccinated children specifically.

*32) Supplementary Figure S3 The vaccines are not listed in the same order for each country which makes comparison between them extremely hard.*

**Response:** Unfortunately, we were not able to change the ordering of vaccines in the new Supplementary Figure S6 due to the algorithm imbedded in the packaged we use for the visualization. In the figure, the intersecting sets of vaccines are ordered from the highest to the lowest number in each country. For easier comparison across countries, in Figure 4b in the manuscript we indicate the vaccines or vaccine combination that are most often missed in each country.

*33) The maps: The authors have for some of the maps (Figures 1 and 3) decided to provided an inset of Central America and the Caribbean. This seems to me a good idea but I would suggest the impact of the maps would be improved by providing an inset for that part of Europe and Asia which contains Albania and Armenia. At the moment they are too insignificant. Space could be made by removing all the Americas apart from the inset. I leave it to the authors whether to split Africa and the rest of Asia and Oceania into separate panels. I would make all the maps consistent, I am not sure why the inset disappears from some of them.*

**Response:** We thank the reviewer for the suggestions on improving the maps. In the updated figures, we provided separate panels for small countries.

*34) What exactly do the clusters mean? More specifically what inference am I expected to draw from them?*

**Response:** In the revised manuscript, the provided more information about the cluster analysis. The spatial clusters indicate the presence of systematic spatial variation across regions, for example, neighboring regions where vaccination coverage is consistently low or high. We tried to improve the language describing the cluster analysis, for example on page 5, lines 72-74:

“Clusters of administrative regions characterized by low vaccine coverage or high degree of socioeconomic inequality are identified using a spatial association technique.”

*35) What influence does it have that the units mentioned in the Supplementary Table S2 seem to be of varying size for different countries? For instance Ethiopia and Gambia have similar numbers of units (11 v 8) but Ethiopia is about 100 times bigger than The Gambia and has about 50 times the population.*

*I have very limited experience of Demographic Surveillance Sites (DSS) but the only one I have visited in Ethiopia (Butajira) is quite small and if that is supposed to represent the whole of the Southern Nations, Nationalities, and Peoples' Region then I am skeptical. If there is only one DSS in Oromia then I find that even more implausible. I suspect this issue means that finding clusters in a country like the Gambia one will be artefactually easier.*

**Response:** DHS surveys are usually representative at administrative level 1 or 2, depending on the country. In the analysis, we followed the DHS survey design and used the administrative divisions at which DHS samples are representative. These can be provinces, districts, states, or other divisions. The number of units in the new Supplementary Table S2 (old Table S2) refers to the number of administrative divisions within each country used in the analysis. We have provided more information on the survey design on page 6, lines 96-106:

“The survey design is based on a two-step sampling procedure [23]. In the first stage, probability samples are drawn from an existing sample frame, usually the most recent census. The sampling frame is divided into subgroups (strata), which are typically geographic regions and urban/rural areas within each region. Within each stratum, primary sampling units (PSUs) are selected with probability proportional to the size within each stratum. In the second stage, a complete household listing is conducted in each PSU and a fixed number of households is selected via systematic sampling. Sampling weights are provided to adjust for differences in the probability of selection and interview. Supplementary Fig S1 shows the location of PSUs in each country included in the analysis. The sampling procedure ensures that the samples are representative both at national and subnational levels (usually administrative level 1 or 2, e.g. region or province) and by urban/rural area.”

Additionally, in Supplementary Figure S1, we plotted the location of PSUs for countries included in the analysis (except for Afghanistan, Maldives, Mauritania, and Indonesia due to lack of geocoordinate information). Across the 1,366 administrative divisions, we have a total of 53,439 PSUs, and within each PSU, 20 to 30 households were interviewed. On average, each administrative division included 39 PSUs, with the smallest number being 8 PSUs in Zabul, Afghanistan, and the highest number being 188 PSUs in West Java, Indonesia. The DHS survey design ensures that data are representative at the specified administrative level.

The lack of finer-scale spatial data and the small sample sizes for some administrative regions is a major limitation, which we mentioned in the limitations section on page 21, lines 372-377:

“At the subnational level, vaccination coverage is estimated at different administrative levels (e.g., states, provinces, or district), depending on the spatial scales at which the DHS surveys are representative, which limits the detection and comparison of spatial clusters. Another limitation concerns the small samples sizes for some administrative regions, which may result in imprecise estimates. We have provided 95% confidence intervals for the inequality indices to account for that.”

*36) Summary: Putting the data on missing vaccinations in front of the readership is clearly a good idea but I need convincing about the merits of the spatial analysis.*

**Response:** Through the spatial analysis we aim to draw attention to the wide variation in vaccination coverage within countries. We demonstrate that the national-level statistics obscure important heterogeneities across administrative regions. We also show that low vaccination rates often coincide with high degree of socioeconomic inequality at the subnational level.

Concerning the spatial autocorrelation analysis, we aim to identify neighboring regions that are similar to one another (e.g., with consistently low levels of immunization coverage). This information can be useful for the design of vaccination campaigns by indicating regions where populations are consistently underserved and where resources can be most effective directed.

# Reviewer 4:

*37) Overall this is a well-written manuscript, with clear descriptions of methodology and of the implications of their findings. The graphical presentation of results in particular is well-done and readily accessible to most readers. The demand for comprehensive sub-national data has grown through the era of COVID-19, and this manuscript has a role in ensuring that low and middle income countries (LMIC) have a place in such future data planning. As noted below, there are some methodological questions the authors need to resolve, such as whether the WHO schedule applied by the authors is actually congruent with individual national immunization schedules. Other questions include whether the selected age range (12 to 35 months) is nationally appropriate as compared to an older (24 to 35 month) age range. Also the authors need to address whether they have assessed on-time immunizations only, as opposed to immunizations given at any age prior to the 12-35 month individual end-point.*

**Response:** We thank the reviewer for the constructive comments, which have assisted greatly us in improving this manuscript. Concerning the immunization schedule, we cross-checked the national immunization plans. All four vaccinations were included in the national plans. However, we found some variations in the measles vaccination schedules for Albania, Armenia, Egypt, Guatemala, India and Jordan. In these countries, measles vaccines were administered up to 12 or 13 months of age. We accounted for these differences by changing the reference population to children between 15 and 35 months of age (allowing for some catch up vaccination). As a robustness check, we calculated vaccination rates and inequality indices for the age group 24-35 months and found comparable results.

Additionally, we clarified in the text that we focus on immunizations given at any time rather than on-time vaccinations. We provide more details in our response to individual comments bellow.

*38) Abstract*

*The claim that this is the first global study looking at sub-national gradients of immunization is somewhat off-target. The DHS survey covers a range of low to middle income countries, rather than the entire globe. There have been previous studies using DHS or MICS to map immunizations across multi-country regions, as the authors note.*

**Response:** We removed from the abstract and the rest of the manuscript the statement that this is a global analysis and refrained from claiming that this is the first analysis of subnational inequalities.

*39) Introduction*

*Pg3Para2: The authors should spell-check their work here.*

**Response:** We have removed existing typos.

*40) Pg3Para2: An extensive body of work exists identifying sub-national areas of low immunization. Such work however is usually on a single country basis, with various methodologies. The authors' text here should be modified to recognize that such work has and is proceeding- and what is needed is a coordinated approach across multiple countries as the authors present.*

**Response:** We revised the text accordingly, highlighting the contribution of this study on page 5, lines 82-84:

“We add to the literature by presenting harmonized and spatially disaggregated estimates of full immunization coverage and wealth-related inequalities in such coverage across multiple LMICs.”

*41) Pg3Para3: As in the introduction, the authors are over-selling their work here as the first global study. This statement should be more conditional, for participating LMICs, and within the context of the DHS.*

**Response:** We revised the text accordingly, removing statements implying that this is a global study.

*42) Pg3Para3:  The subclause "(with the finest spatial resolution…)" should be removed, as this belongs in the methodology discussion about mapping techniques.*

**Response:** We revised the text accordingly.

*43) Pg4Para3:  This paragraph would be better placed in the Discussion section. It should also have a sentence regarding the rapid evolution of detailed global immunization databases around COVID, (Guidotti, 2022), and who LMIC are being left out of this evolution- definitely support for a niche program as the authors propose.*

**Response:** We thank the reviewer for the suggested literature, which we referenced on page 19, lines 323-325:

“Moreover, LMICs are consistently left behind when it comes to the rapid evolution of global immunization databases, which were developed in response to the COVID-19 pandemic and provide up-to-date information at a fine temporal and spatial resolution [52]. Subnational heterogeneities in essential immunization remain understudied in LMICs, which presents a major barrier to the design and implementation of context-specific interventions there [53].”

As suggested, we moved the last paragraphs of the Introduction section to the Discussion section.

*44) Study measures*

*Pg6Para2: The analysis is per the EPI schedule- however what proportion of LMICs exactly mimic this in their national schedules? MCV is listed at 9 months of age, but many LMIC list a minimum age of 12 months as is common in higher income nations. Similarly some national schedules call for BCG at birth, without provision for later catchup.*

*Pg6Para2: An issue with the current study is the inclusion of children from ages 12 to 35 month without assessing the relation between age and full immunization. Would the results appear different if limited to children age 24 to 35 months, as this would accommodate more local variation in immunization practice and some catchup opportunity?*

**Response:** We thank the reviewer for this comment. We cross-checked the immunization schedules for each country, and indeed we identified a few countries where the schedule differs from the recommended EPI schedule. In particular, in Albania, Armenia, Egypt, Guatemala, India and Jordan measles vaccines are administered up to 12 or 13 months of age. For comparability and to allow for some catchup, we decided to focus on children between 15 and 35 months of age. As a robustness analysis, we also calculated immunization rates and inequality indices for the 24 to 25 months age group at the national level and the results were comparable. At the subnational level, we wanted to ensure that the samples are large enough to be representative and only presented results for 15- to 35-month-olds.

*45) Pg6Para2: The text suggests that immunizations were only counted if received by the EPI schedule due date- the authors should confirm if this is true, and if so then rerun results accounting for a longer period. Otherwise the language describing the study should be changed to reflect that this is an analysis of on-time immunization.*

**Response:** We clarified in the text that we focus on immunizations given at any time, page 7, lines 139-140:

“We generated a series of binary variables indicating whether the child had received each of the vaccines at any age.”

*46) Pg6para3: The use of the Concentration Indexes is interesting here- I would suggest that the authors also present charts showing the relation between the probability of immunization against the underlying Wealth Index to provide more context to readers.*

**Response:** We provided a graphical presentation of the concentration curve in Supplementary Figure S2 to demonstrate the relationship between the wealth rank and the probability of immunization for one country.

*47) Spatial cluster analysis*

*Pg8Para2: The authors adjust for FDR (false discovery rate) using an approach from Castro et al (2006). However more recent work by Sun et al (2015) should be considered.*

***Response:*** We thank the reviewer for the suggested literature and the alternative procedure developed by Sun et al (2015). We have not been able to update the analysis with the proposed approach due to the limited time available for revising the manuscript. Still, we relied on an approach which is widely used in the literature. For the future, we will consider the more innovative solution proposed.

*48) Discussion*

*Pg15Para4: The authors should discuss and cite to prior studies examining wealth or income and likelihood of childhood immunization. In higher income nations it is well established that sub-populations with high levels of income are associated with lower immunization rates. An open question here (for DHS data) is whether the determination of wealth is able to distinguish such high income subpopulations in each locale that would be more likely to avoid immunization. This is a caveat on statements regarding wealth and immunization.*

***Response:*** *We thank the reviewer for this suggestion. We added a discussion of pro-poo inequalities, which were identified in the study for certain countries and administrative regions, and we discussed our findings in relation to earlier research on vaccine hesitancy. Page 20, lines 355-363:*

*“Interestingly, in a few countries we found that children from wealthier households were less likely to be fully immunized. This was particularly the case in Armenia, Tajikistan, and Mauritania. Patterns of increasing pro-poor inequality in child immunization across some LMICs have been reported in the literature before [15,17]. The emergence of vaccine hesitancy, which is usually observed in more economically developed countries [57,58], could explain this phenomenon [59]. Various factors may influence vaccine hesitancy, such as parent’s perception of the risks and benefits of child immunization. However, knowledge about vaccine hesitancy and its impact on vaccine uptake in low-income countries is still limited [59,60]. Better understanding of these trends in LMICs is needed to ensure the success of future vaccination campaigns.”*
