# Supplementary material for: Essential childhood immunization in 43 low- and middle-income countries: Analysis of spatial trends and socioeconomic inequalities in vaccine coverage
Source: PLoS Med. 2023 Jan 17;20(1):e1004166. doi: 10.1371/journal.pmed.1004166 (PMC9888726; doi:10.1371/journal.pmed.1004166)
Supplement: Attachment — Submitted filename: Reponse.docx [file pmed.1004166.s015.docx]

Academic Editor:

*Author summary
Line 39: “…children from pooper…” suggest  “poorer”?
Please check spelling and grammar throughout for minor errors*

**Response:** We thank the editor for identifying the grammar mistake. We checked the whole manuscript for grammar and spelling mistakes.

*Introduction
Suggest moving paragraph starting at lines 67-76, to the final paragraph of the introduction.  The introduction should conclude with a final paragraph clearly stating the study aim.
If there has been a systematic review of the evidence related to your study (or you have conducted one), please refer to and reference that review and indicate whether it supports the need for your study.*

**Response:** Following the editor’s suggestion, we moved the indicated paragraph to the end of the introduction section.

Additionally

*Tables and Figures
In figure 2 and 3 captions you write “inex” do you mean index? Please revise*

**Response:** Thank you for identifying this spelling mistake. We checked the manuscript for grammar and spelling mistakes.

*Figure S6: it would be helpful to explicitly state in the caption that the dark dots and lines represent missed vaccines – its not easy at first glance to understand which (light Vs dark dots) represent missed vaccine doses*

**Response:** We improved the caption of Figure S6. The new caption reads:

“Fig S6. Intersecting sets of missed vaccinations for children aged 15 to 35 months for 43 countries. The black dots represent vaccine combinations and the bars represent the number of missed vaccinations for each vaccine combination. Note that multiple doses of DTP and OPV are needed to reach full immunization, therefore, the presented estimates do not refer to the number of missed doses but complete vaccinations. If a child is missing two or more doses of a specific vaccine, the child will be counted only once.”

Reviewer 1:

*The authors have satisfactorily addressed all my comments on the previous version of the paper.

I only have a minor comment that can be addressed in the limitation section of the paper. As the survey year for the countries includes the time period before and during COVID-19 pandemic (2014-2021), the vaccination rates of childhood vaccination may have impacted following the pandemic for all countries but the changes in vaccination only captured in countries with the survey years of 2020 and 2021.*

**Response:** We thank the reviewer for raising this issue. We included a discussion of this issue in the limitations section, page 21, lines 548-552:

“Moreover, the period of data collection ranges from 2014 and 2021, which may affect the comparability of results across countries. In a few countries included in the analysis, survey data were collected after the onset of the COVID-19 pandemic in early 2020. In the majority of countries, however, data were collected before 2020, which implies that the disruption of vaccination efforts due to the COVID-19 pandemic will not be captured there.”

Reviewer 2:

*I appreciate the authors' thoughtful revisions and attention to these comments. I have a few minor follow-up comments regarding the limitations, but otherwise most of my comments have been thoroughly addressed.

Major comments:
1.      Previous major comment #1 (regarding uncertainty): I thank the authors for providing uncertainty estimates for the concentration indexes. As expected, some of the confidence intervals are somewhat broad, but their inclusion in the tables and figures is a substantial improvement to the paper. In particular the supplementary figure S3 - combined with the previous figures - is quite helpful. No further comments.*

**Response:** We thank the reviewer for the suggestion to include uncertainty estimates for the concentration indices, which has greatly improved the analysis.

*2.      Previous major comment #2 (regarding the need to add text describing the limitations of the analysis). The added text for the limitations is much appreciated and thoughtful. I have two follow-up questions from this added text:

2a.      The authors write: "The vaccination information was verified via vaccination cards for 67% of children in the sample. For the rest, this information was based on the mother's recall, which is subject to recall bias. However, the validity of relying on parental reporting of immunization has been verified in the literature [63]"

The cited article is from a convenience sample of 108 children in a high income setting in the early 2000s; the literature on the reliability of parental recall is much more mixed than this statement would suggest. See for instance Dansereau et al 2020 (*[*https://urldefense.com/v3/__https://pubmed.ncbi.nlm.nih.gov/32270134/__;!!Mih3wA!Hx1GAL6hrj3EYkCdc7ilhidbe9amqtyybFG7-vnipunkJSlJtkKuA94rDnRhG_nnD9Xy_RK0aAJ0JXJ75w$*](https://urldefense.com/v3/__https:/pubmed.ncbi.nlm.nih.gov/32270134/__;!!Mih3wA!Hx1GAL6hrj3EYkCdc7ilhidbe9amqtyybFG7-vnipunkJSlJtkKuA94rDnRhG_nnD9Xy_RK0aAJ0JXJ75w$) *) which suggests that the validity of recall compared to home-based and facility-based methods varies broadly in low- and middle-income countries. I would suggest that the authors might want to capture some of this nuance in their phrasing to more completely illustrate the challenges inherent in relying on maternal recall data.*

**Response:** We thank the reviewer for the suggested literature. We revised the statement accordingly.  *2b. The authors write: "Another limitation is that we have not been able to distinguish between vaccines administered as part of routine immunization services versus immunization campaigns, which may be less effective in the long term [64], due to lack of such data."

Similar to my comment above, the literature on immunization campaigns is perhaps more nuanced than suggested here. For instance, Portnoy et al 2020 examined survey data, finding that supplemental immunization activities (campaigns) tended to have a more pro-equity distributional impact across wealth quintiles than routine immunization (*[*https://urldefense.com/v3/__https://www.ncbi.nlm.nih.gov/pmc/articles/PMC7519803/__;!!Mih3wA!Hx1GAL6hrj3EYkCdc7ilhidbe9amqtyybFG7-vnipunkJSlJtkKuA94rDnRhG_nnD9Xy_RK0aAKtOX3VWw$*](https://urldefense.com/v3/__https:/www.ncbi.nlm.nih.gov/pmc/articles/PMC7519803/__;!!Mih3wA!Hx1GAL6hrj3EYkCdc7ilhidbe9amqtyybFG7-vnipunkJSlJtkKuA94rDnRhG_nnD9Xy_RK0aAKtOX3VWw$) *). The authors might consider rephrasing this section to account for this sort of nuance.*

**Response:** We are very thankful to the reviewer for the suggested literature. We have revised the text accordingly, including a more nuanced discussion of the literature.  *Minor comments:*

*1.      Minor comment #1 (regarding MCV1 age of administration and the ages of analysis). I appreciate the change to the 15-35 month reference age range to deal with this limitation. As the authors note, this will improve comparability across countries although does result (likely) in more catch-up vaccination being captured, where relevant. While there is no perfect solution to these questions, though, the switch (and sensitivity analysis among 24-35 month olds in tables S3/S4) is well-reasoned and explained in the text and strengthens the paper. No further comments.*

*2.      Minor comment #2 (regarding RI vs campaign doses). As above, I appreciate the addition of this to the limitations section; see my comment above regarding the validity of parental recall but otherwise I have no additional comments in this regard.*

*3.      Minor comment #3 (regarding other types of analyses of inequality). The added text describing other dimensions of inequality in vaccination coverage is much appreciated, as is the broader appeal to available literature. I thank the authors for these additions, which increase the richness of the discussion, and have no further comments in this area.*

*4.      Minor comment #4 (regarding language describing the scale of spatial heterogeneities assessed here). I appreciate that the authors have taken care to more accurately describe these subnational levels of analysis throughout the text, and have added the differing spatial scales across countries to the limitations of the analysis. No further comments.*

**Response:** We thank the reviewer for all of the above suggestions, which have helped us greatly in improving the manuscript.

Reviewer 3:

*The authors have addressed my points.*

Reviewer 4:

*The authors have largely addressed my initial concerns with their reviewer responses. What is still needed in this manuscript is for the authors to run a final check for spelling and grammar, as a few instances of both are over the top. For example, line 39 of page 3. Otherwise, given the limitations of DHS data, I believe the authors have done a credible job in their analysis and write-up.*

**Response:** We thank the reviewer for pointing out our spelling and grammar mistakes. We proofread the manuscript, correcting all mistakes.
